# Supplementary material for: Variation at an adhesin locus suggests sociality in natural populations of the yeast Saccharomyces cerevisiae
Source: Proc Biol Sci. 2019 Oct 16;286(1913):20191948. doi: 10.1098/rspb.2019.1948 (PMC6834051; doi:10.1098/rspb.2019.1948)
Supplement: Supplementary Material [file rspb20191948supp1.pdf]

Supplementary Information

Figure S1: Biofilm phenotypes of the 78-Strain panel.
Figure S2: A domain amino acid alignment.
Figure S3: C domain amino acid alignment.
Figure S4: Inferred breakpoints via GARD.
Figure S5: Genetic variation at *FLO11* compared to variation in genes that code for proteins with similar properties.
Figure S6: Upstream regulatory alignment.
Figure S7: Distribution of Tajima's D values.
Figure S8: Distribution of social scores among the regulatory clades
Figure S9: Functional assay of natural *FLO11* alleles.

Table S1: Strain table and data summary
Table S2: Genes for cell wall and GPI-anchored proteins
Table S3: Strains used in the cell wall and GPI-anchored genes analysis
Table S4: Strains for functional analysis
Table S5: Primers
Table S6: PAML model results for the full data set and various trees
Table S7: PAML significant sites associated with Table S2
Table S8: PAML model results separating strains with and without the insert
Table S9: PAML significant sites associated with Table S4
Table S10: GARD results
Table S11: PAML model results for recombination segments
Table S12: PAML significant sites associated with Table S6
Table S13: ANOVA analysis of *prFLO11-GFP* expression

Data files: Fasta files and PAML files will be included.

Figure S1: Biofilm phenotypes of the 78-Strain panel. (A) Scale for scoring biofilm mats and colonies. (B) Distribution of average social scores. (C) Complex colony morphology on a low dextrose 2% agar plate. (D) Mat biofilms formed on low dextrose 0.3% agar plates. In both C and D, samples 1-78 (as described in Table S1) are arrayed in order from A1-A12, B1-B12, etc.

A

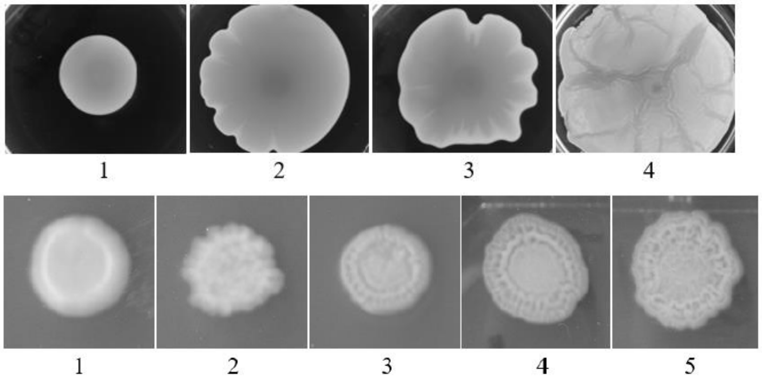

B

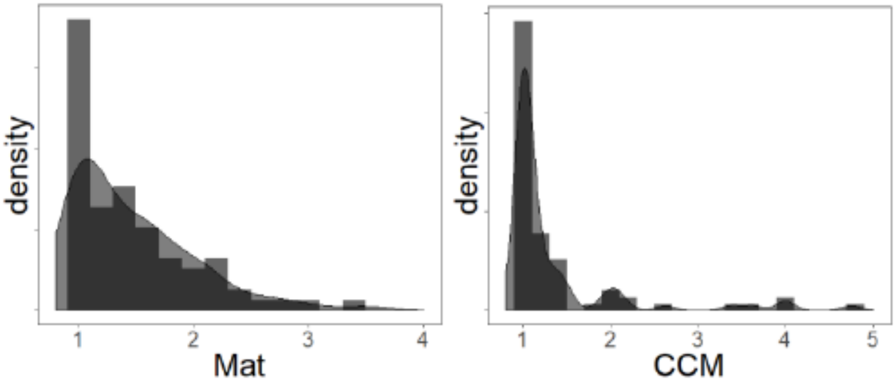

C

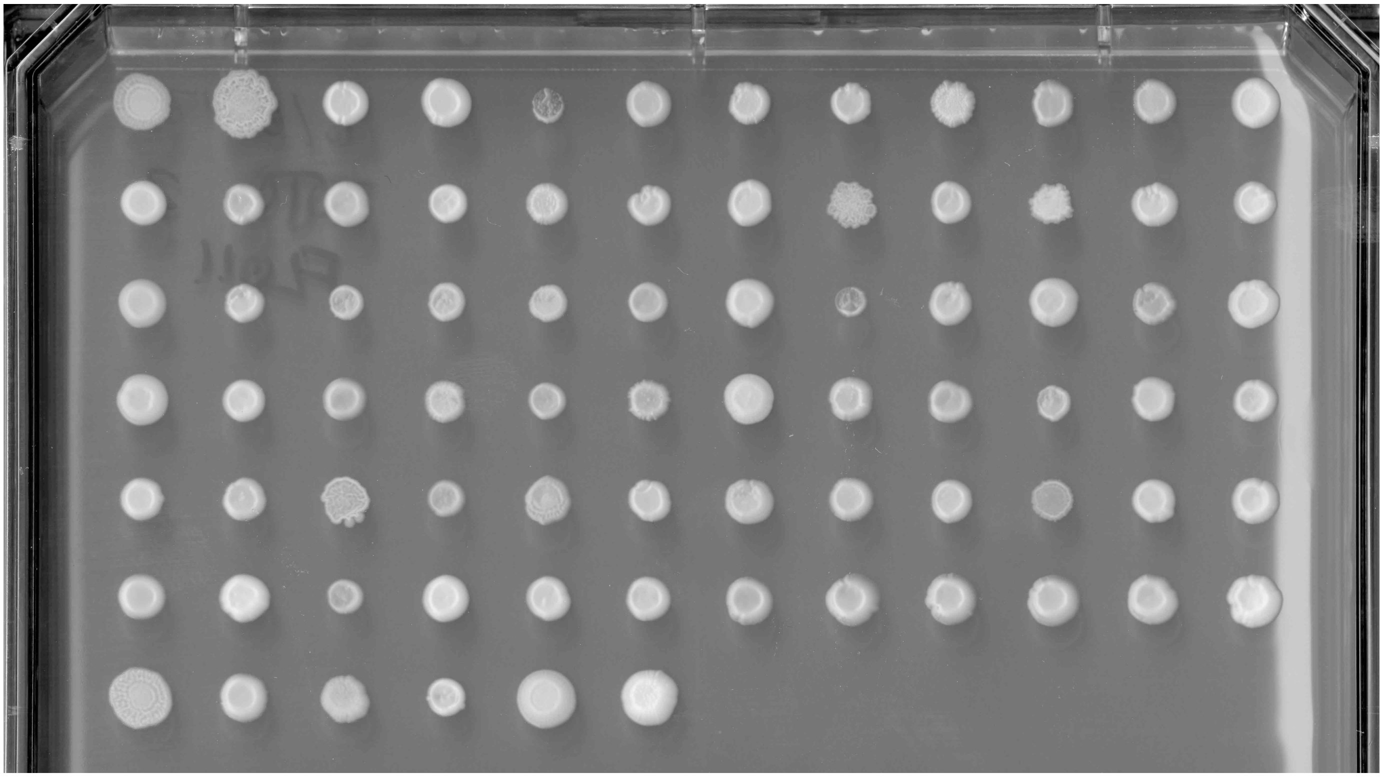

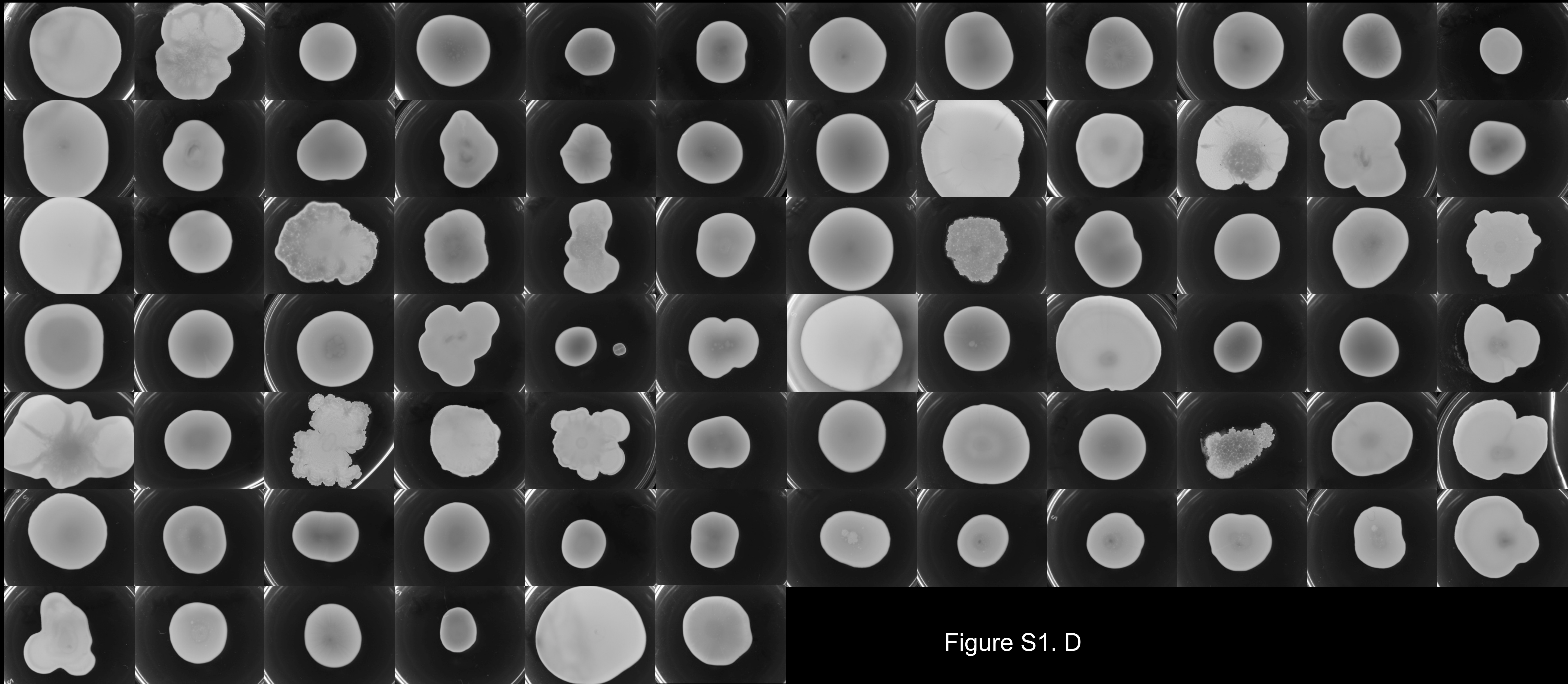

Figure S1. D

Figure S2: A domain amino acid alignment. Gray boxes are beta sheets; red, green and blue are apical regions, as in Figure 1. Black stars indicate aromatic band I in the apical region; gray stars indicate aromatic band II in the neck subdomain.

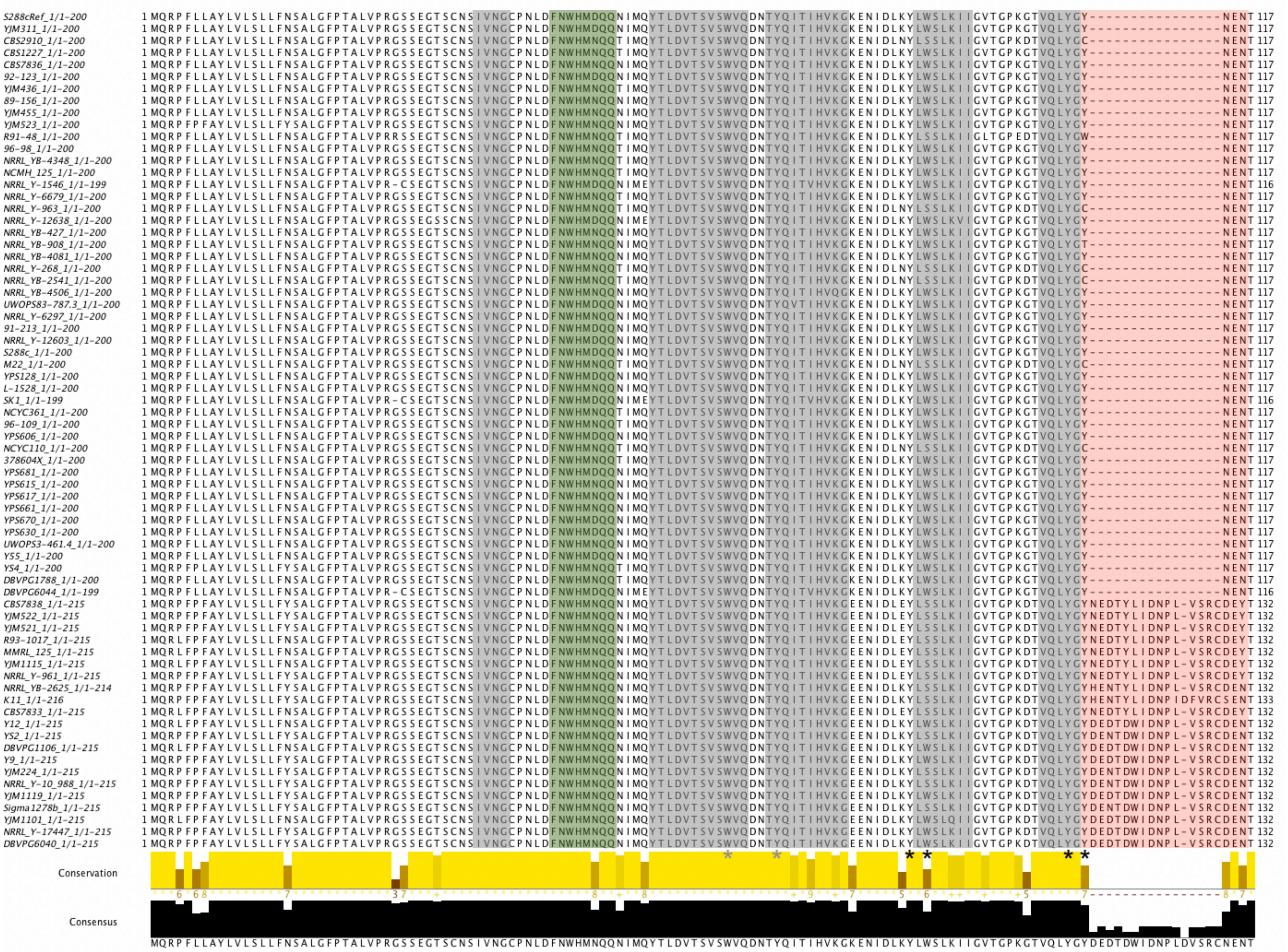

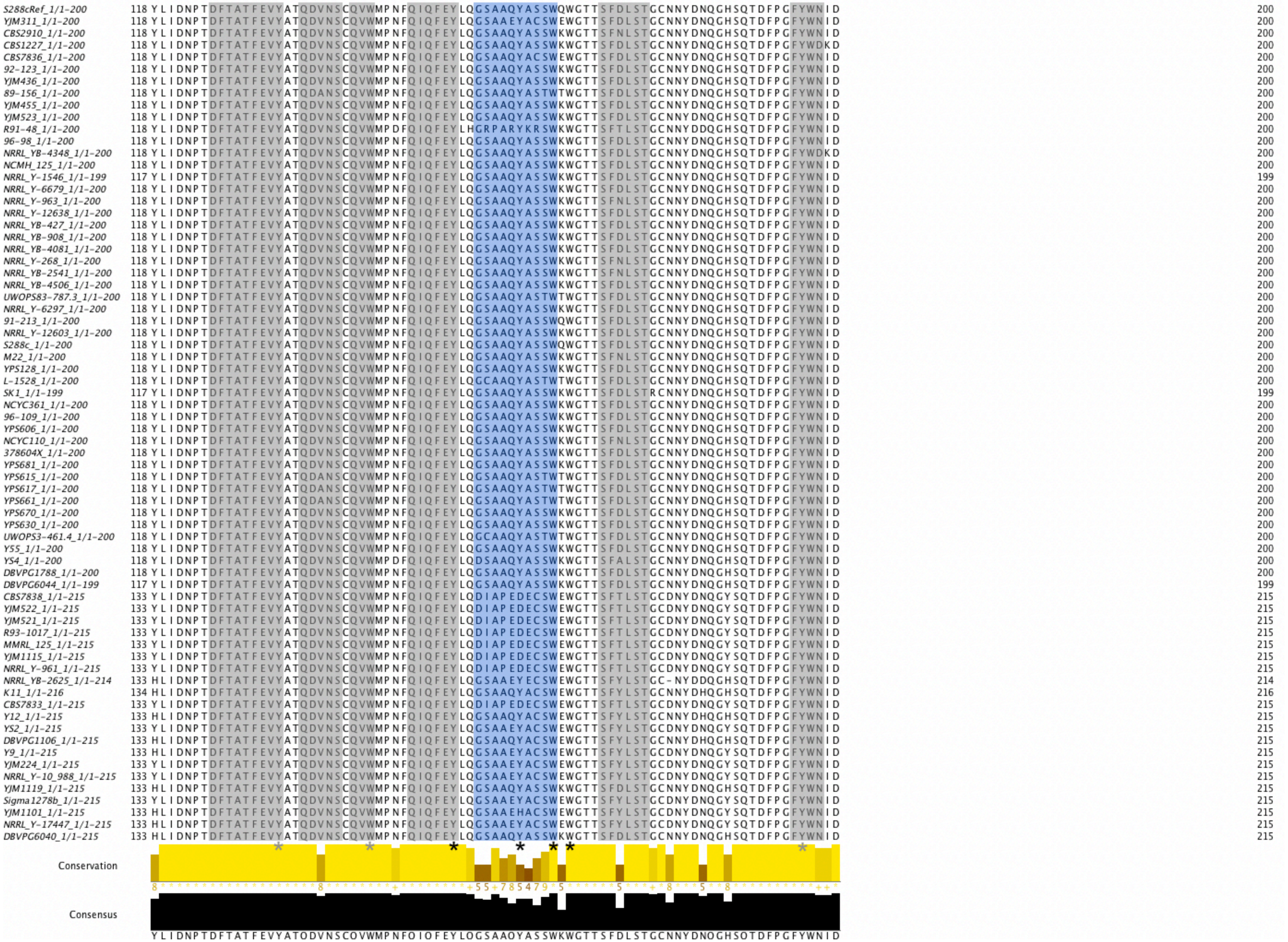
S288cRef_1/1-200 118 YLIDNPTDFTATFEVYATQDVNSCQVWMPNFQIQFEYLQGSAAQYASSWQWGTTSFDLSTGCNNYDNQGHSQTDFPGFYWNID 200
YJM311_1/1-200 118 YLIDNPTDFTATFEVYATQDVNSCQVWMPNFQIQFEYLQGSAAEYACSWEWGTTSFDLSTGCNNYDNQGHSQTDFPGFYWNID 200
CBS2910_1/1-200 118 YLIDNPTDFTATFEVYATQDVNSCQVWMPNFQIQFEYLQGSAAQYASSWKWGTTSFNLSTGCNNYDNQGHSQTDFPGFYWNID 200
CBS1227_1/1-200 118 YLIDNPTDFTATFEVYATQDVNSCQVWMPNFQIQFEYLQGSAAQYASSWKWGTTSFDLSTGCNNYDNQGHSQTDFPGFYWDKD 200
CBS7836_1/1-200 118 YLIDNPTDFTATFEVYATQDVNSCQVWMPNFQIQFEYLQGSAAQYACSWEWGTTSFDLSTGCNNYDNQGHSQTDFPGFYWNID 200
92-123_1/1-200 118 YLIDNPTDFTATFEVYATQDVNSCQVWMPNFQIQFEYLQGSAAQYASSWKWGTTSFDLSTGCNNYDNQGHSQTDFPGFYWNID 200
YJM436_1/1-200 118 YLIDNPTDFTATFEVYATQDVNSCQVWMPNFQIQFEYLQGSAAQYASSWKWGTTSFDLSTGCNNYDNQGHSQTDFPGFYWNID 200
89-156_1/1-200 118 YLIDNPTDFTATFEVYATQDANSCQVWMPNFQIQFEYLQGSAAQYASTWTWGTTSFDLSTGCNNYDNQGHSQTDFPGFYWNID 200
YJM455_1/1-200 118 YLIDNPTDFTATFEVYATQDVNSCQVWMPNFQIQFEYLQGSAAQYASSWKWGTTSFDLSTGCNNYDNQGHSQTDFPGFYWNID 200
YJM523_1/1-200 118 YLIDNPTDFTATFEVYATQDVNSCQVWMPNFQIQFEYLQGSAAQYASSWKWGTTSFDLSTGCNNYDNQGHSQTDFPGFYWNID 200
R91-48_1/1-200 118 YLIDNPTDFTATFEVYATQDVNSCQVWMPDFQIQFEYLHGRPARYKRSWKWGTTSFTLSTGCNNYDDQGHSQTDFPGFYWNID 200
96-98_1/1-200 118 YLIDNPTDFTATFEVYATQDVNSCQVWMPNFQIQFEYLQGSAAQYASSWKWGTTSFDLSTGCNNYDNQGHSQTDFPGFYWNID 200
NRRL_YB-4348_1/1-200 118 YLIDNPTDFTATFEVYATQDVNSCQVWMPNFQIQFEYLQGSAAQYASSWKWGTTSFDLSTGCNNYDNQGHSQTDFPGFYWDKD 200
NCMH_125_1/1-200 118 YLIDNPTDFTATFEVYATQDVNSCQVWMPNFQIQFEYLQGSAAQYASSWKWGTTSFDLSTGCNNYDNQGHSQTDFPGFYWNID 200
NRRL_Y-1546_1/1-199 117 YLIDNPTDFTATFEVYATQDVNSCQVWMPNFQIQFEYLQGSAAQYASSWKWGTTSFDLSTGCNNYDNQGHSQTDFPGFYWNID 199
NRRL_Y-6679_1/1-200 118 YLIDNPTDFTATFEVYATQDVNSCQVWMPNFQIQFEYLQGSAAQYASSWKWGTTSFDLSTGCNNYDNQGHSQTDFPGFYWNID 200
NRRL_Y-963_1/1-200 118 YLIDNPTDFTATFEVYATQDVNSCQVWMPNFQIQFEYLQGSAAQYASSWKWGTTSFNLSTGCNNYDNQGHSQTDFPGFYWNID 200
NRRL_Y-12638_1/1-200 118 YLIDNPTDFTATFEVYATQDVNSCQVWMPNFQIQFEYLQGSAAQYASSWKWGTTSFDLSTGCNNYDNQGHSQTDFPGFYWNID 200
NRRL_YB-427_1/1-200 118 YLIDNPTDFTATFEVYATQDVNSCQVWMPNFQIQFEYLQGSAAQYASSWKWGTTSFDLSTGCNNYDNQGHSQTDFPGFYWNID 200
NRRL_YB-908_1/1-200 118 YLIDNPTDFTATFEVYATQDVNSCQVWMPNFQIQFEYLQGSAAQYASSWKWGTTSFDLSTGCNNYDNQGHSQTDFPGFYWNID 200
NRRL_YB-4081_1/1-200 118 YLIDNPTDFTATFEVYATQDVNSCQVWMPNFQIQFEYLQGSAAQYASSWKWGTTSFDLSTGCNNYDNQGHSQTDFPGFYWNID 200
NRRL_Y-268_1/1-200 118 YLIDNPTDFTATFEVYATQDVNSCQVWMPNFQIQFEYLQGSAAQYASSWKWGTTSFNLSTGCNNYDNQGHSQTDFPGFYWNID 200
NRRL_YB-2541_1/1-200 118 YLIDNPTDFTATFEVYATQDVNSCQVWMPNFQIQFEYLQGSAAQYASSWKWGTTSFNLSTGCNNYDNQGHSQTDFPGFYWNID 200
NRRL_YB-4506_1/1-200 118 YLIDNPTDFTATFEVYATQDVNSCQVWMPNFQIQFEYLQGSAAQYASSWKWGTTSFDLSTGCNNYDNQGHSQTDFPGFYWNID 200
UWOPS83-787.3_1/1-200 118 YLIDNPTDFTATFEVYATQDVNSCQVWMPNFQIQFEYLQGSAAQYASTWTWGTTSFDLSTGCNNYDNQGHSQTDFPGFYWNID 200
NRRL_Y-6297_1/1-200 118 YLIDNPTDFTATFEVYATQDVNSCQVWMPNFQIQFEYLQGSAAQYASSWKWGTTSFDLSTGCNNYDNQGHSQTDFPGFYWNID 200
91-213_1/1-200 118 YLIDNPTDFTATFEVYATQDVNSCQVWMPNFQIQFEYLQGSAAQYASSWQWGTTSFDLSTGCNNYDNQGHSQTDFPGFYWNID 200
NRRL_Y-12603_1/1-200 118 YLIDNPTDFTATFEVYATQDVNSCQVWMPNFQIQFEYLQGSAAQYASSWKWGTTSFDLSTGCNNYDNQGHSQTDFPGFYWNID 200
S288c_1/1-200 118 YLIDNPTDFTATFEVYATQDVNSCQVWMPNFQIQFEYLQGSAAQYASSWQWGTTSFDLSTGCNNYDNQGHSQTDFPGFYWNID 200
M22_1/1-200 118 YLIDNPTDFTATFEVYATQDVNSCQVWMPNFQIQFEYLQGSAAQYASSWKWGTTSFNLSTGCNNYDNQGHSQTDFPGFYWNID 200
YPS128_1/1-200 118 YLIDNPTDFTATFEVYATQDVNSCQVWMPNFQIQFEYLQGSAAQYASSWKWGTTSFDLSTGCNNYDNQGHSQTDFPGFYWNID 200
L-1528_1/1-200 118 YLIDNPTDFTATFEVYATQDVNSCQVWMPNFQIQFEYLQGCAAQYASTWTWGTTSFDLSTGCNNYDNQGHSQTDFPGFYWNID 200
SK1_1/1-199 117 YLIDNPTDFTATFEVYATQDVNSCQVWMPNFQIQFEYLQGSAAQYASSWKWGTTSFDLSTRCNNYDNQGHSQTDFPGFYWNID 199
NCYC361_1/1-200 118 YLIDNPTDFTATFEVYATQDVNSCQVWMPNFQIQFEYLQGSAAQYASSWKWGTTSFDLSTGCNNYDNQGHSQTDFPGFYWNID 200
96-109_1/1-200 118 YLIDNPTDFTATFEVYATQDVNSCQVWMPNFQIQFEYLQGSAAQYASSWKWGTTSFDLSTGCNNYDNQGHSQTDFPGFYWNID 200
YPS606_1/1-200 118 YLIDNPTDFTATFEVYATQDVNSCQVWMPNFQIQFEYLQGSAAQYASSWKWGTTSFDLSTGCNNYDNQGHSQTDFPGFYWNID 200
NCYC110_1/1-200 118 YLIDNPTDFTATFEVYATQDVNSCQVWMPNFQIQFEYLQGSAAQYASSWKWGTTSFNLSTGCNNYDNQGHSQTDFPGFYWNID 200
378604X_1/1-200 118 YLIDNPTDFTATFEVYATQDVNSCQVWMPNFQIQFEYLQGSAAQYASSWKWGTTSFDLSTGCNNYDNQGHSQTDFPGFYWNID 200
YPS681_1/1-200 118 YLIDNPTDFTATFEVYATQDVNSCQVWMPNFQIQFEYLQGSAAQYASSWKWGTTSFDLSTGCNNYDNQGHSQTDFPGFYWNID 200
YPS615_1/1-200 118 YLIDNPTDFTATFEVYATQDANSCQVWMPNFQIQFEYLQGSAAQYASTWTWGTTSFDLSTGCNNYDNQGHSQTDFPGFYWNID 200
YPS617_1/1-200 118 YLIDNPTDFTATFEVYATQDANSCQVWMPNFQIQFEYLQGSAAQYASTWTWGTTSFDLSTGCNNYDNQGHSQTDFPGFYWNID 200
YPS661_1/1-200 118 YLIDNPTDFTATFEVYATQDANSCQVWMPNFQIQFEYLQGSAAQYASTWTWGTTSFDLSTGCNNYDNQGHSQTDFPGFYWNID 200
YPS670_1/1-200 118 YLIDNPTDFTATFEVYATQDVNSCQVWMPNFQIQFEYLQGSAAQYASSWKWGTTSFDLSTGCNNYDNQGHSQTDFPGFYWNID 200
YPS630_1/1-200 118 YLIDNPTDFTATFEVYATQDVNSCQVWMPNFQIQFEYLQGSAAQYASSWKWGTTSFDLSTGCNNYDNQGHSQTDFPGFYWNID 200
UWOPS3-461.4_1/1-200 118 YLIDNPTDFTATFEVYATQDVNSCQVWMPNFQIQFEYLQGCAAQYASTWTWGTTSFDLSTGCNNYDNQGHSQTDFPGFYWNID 200
Y55_1/1-200 118 YLIDNPTDFTATFEVYATQDVNSCQVWMPNFQIQFEYLQGSAAQYASSWKWGTTSFDLSTGCNNYDNQGHSQTDFPGFYWNID 200
YS4_1/1-200 118 YLIDNPTDFTATFEVYATQDVNSCQVWMPDFQIQFEYLQDSAAQYASSWKWGTTSFALSTGCNNYDNQGHSQTDFPGFYWNID 200
DBVPG1788_1/1-200 118 YLIDNPTDFTATFEVYATQDVNSCQVWMPNFQIQFEYLQGSAAQYASSWKWGTTSFDLSTGCNNYDNQGHSQTDFPGFYWNID 200
DBVPG6044_1/1-199 117 YLIDNPTDFTATFEVYATQDVNSCQVWMPNFQIQFEYLQGSAAQYASSWKWGTTSFDLSTGCNNYDNQGHSQTDFPGFYWNID 199
CBS7838_1/1-215 133 YLIDNPTDFTATFEVYATQDVNSCQVWMPNFQIQFEYLQDIAPEDECSWEWGTTSFTLSTGCDNYDNQGYSQTDFPGFYWNID 215
YJM522_1/1-215 133 YLIDNPTDFTATFEVYATQDVNSCQVWMPNFQIQFEYLQDIAPEDECSWEWGTTSFTLSTGCDNYDNQGYSQTDFPGFYWNID 215
YJM521_1/1-215 133 YLIDNPTDFTATFEVYATQDVNSCQVWMPNFQIQFEYLQDIAPEDECSWEWGTTSFTLSTGCDNYDNQGYSQTDFPGFYWNID 215
R93-1017_1/1-215 133 YLIDNPTDFTATFEVYATQDVNSCQVWMPNFQIQFEYLQDIAPEDECSWEWGTTSFTLSTGCDNYDNQGYSQTDFPGFYWNID 215
MMRL_125_1/1-215 133 YLIDNPTDFTATFEVYATQDVNSCQVWMPNFQIQFEYLQDIAPEDECSWEWGTTSFTLSTGCDNYDNQGYSQTDFPGFYWNID 215
YJM1115_1/1-215 133 YLIDNPTDFTATFEVYATQDVNSCQVWMPNFQIQFEYLQDIAPEDECSWEWGTTSFTLSTGCDNYDNQGYSQTDFPGFYWNID 215
NRRL_Y-961_1/1-215 133 YLIDNPTDFTATFEVYATQDVNSCQVWMPNFQIQFEYLQDIAPEDECSWEWGTTSFTLSTGCDNYDNQGYSQTDFPGFYWNID 215
NRRL_YB-2625_1/1-214 133 HLIDNPTDFTATFEVYATQDVNSCQVWMPNFQIQFEYLQGSAAEYECSWEWGTTSFYLSTGC-NYDDQGHSQTDFPGFYWNID 214
K11_1/1-216 134 HLIDNPTDFTATFEVYATQDVNSCQVWMPNFQIQFEYLQGSAAQYACSWEWGTTSFYLSTGCNNYDHQGHSQTDFPGFYWNID 216
CBS7833_1/1-215 133 YLIDNPTDFTATFEVYATQDVNSCQVWMPNFQIQFEYLQDIAPEDECSWEWGTTSFTLSTGCDNYDNQGYSQTDFPGFYWNID 215
Y12_1/1-215 133 HLIDNPTDFTATFEVYATQDVNSCQVWMPNFQIQFEYLQGSAAQYACSWEWGTTSFYLSTGCNNYDHQGHSQTDFPGFYWNID 215
YS2_1/1-215 133 YLIDNPTDFTATFEVYATQDVNSCQVWMPNFQIQFEYLQGSAAEYACSWEWGTTSFYLSTGCDNYDNQGYSQTDFPGFYWNID 215
DBVPG1106_1/1-215 133 HLIDNPTDFTATFEVYATQDVNSCQVWMPNFQIQFEYLQGSAAQYACSWEWGTTSFYLSTGCNNYDHQGHSQTDFPGFYWNID 215
Y9_1/1-215 133 HLIDNPTDFTATFEVYATQDVNSCQVWMPNFQIQFEYLQGSAAEYACSWEWGTTSFYLSTGCDNYDNQGYSQTDFPGFYWNID 215
YJM224_1/1-215 133 YLIDNPTDFTATFEVYATQDVNSCQVWMPNFQIQFEYLQGSAAEYACSWEWGTTSFYLSTGCDNYDNQGYSQTDFPGFYWNID 215
NRRL_Y-10_988_1/1-215 133 YLIDNPTDFTATFEVYATQDVNSCQVWMPNFQIQFEYLQGSAAEYACSWEWGTTSFYLSTGCDNYDNQGYSQTDFPGFYWNID 215
YJM1119_1/1-215 133 HLIDNPTDFTATFEVYATQDVNSCQVWMPNFQIQFEYLQGSAAQYASSWKWGTTSFDLSTGCNNYDNQGHSQTDFPGFYWNID 215
Sigma1278b_1/1-215 133 YLIDNPTDFTATFEVYATQDVNSCQVWMPNFQIQFEYLQGSAAEYACSWEWGTTSFYLSTGCDNYDNQGYSQTDFPGFYWNID 215
YJM1101_1/1-215 133 HLIDNPTDFTATFEVYATQDVNSCQVWMPNFQIQFEYLQGSAAEHACSWEWGTTSFYLSTGCDNYDNQGYSQTDFPGFYWNID 215
NRRL_Y-17447_1/1-215 133 HLIDNPTDFTATFEVYATQDVNSCQVWMPNFQIQFEYLQGSAAEYACSWEWGTTSFYLSTGCDNYDNQGYSQTDFPGFYWNID 215
DBVPG6040_1/1-215 133 HLIDNPTDFTATFEVYATQDVNSCQVWMPNFQIQFEYLQGSAAQYASSWKWGTTSFDLSTGCNNYDNQGHSQTDFPGFYWNID 215
Conservation
Consensus
YLIDNPTDFTATFEVYATODVNSCOVWMPNFOIOFEYLOGSAAOYASSWKWGTTSFDLSTGCNNYDNOGHSOTDFPGFYWNID

Figure S3: C domain amino acid alignment. Black line indicates location that defined C-domain in our analysis. Gray domain indicates GPI-anchored area.

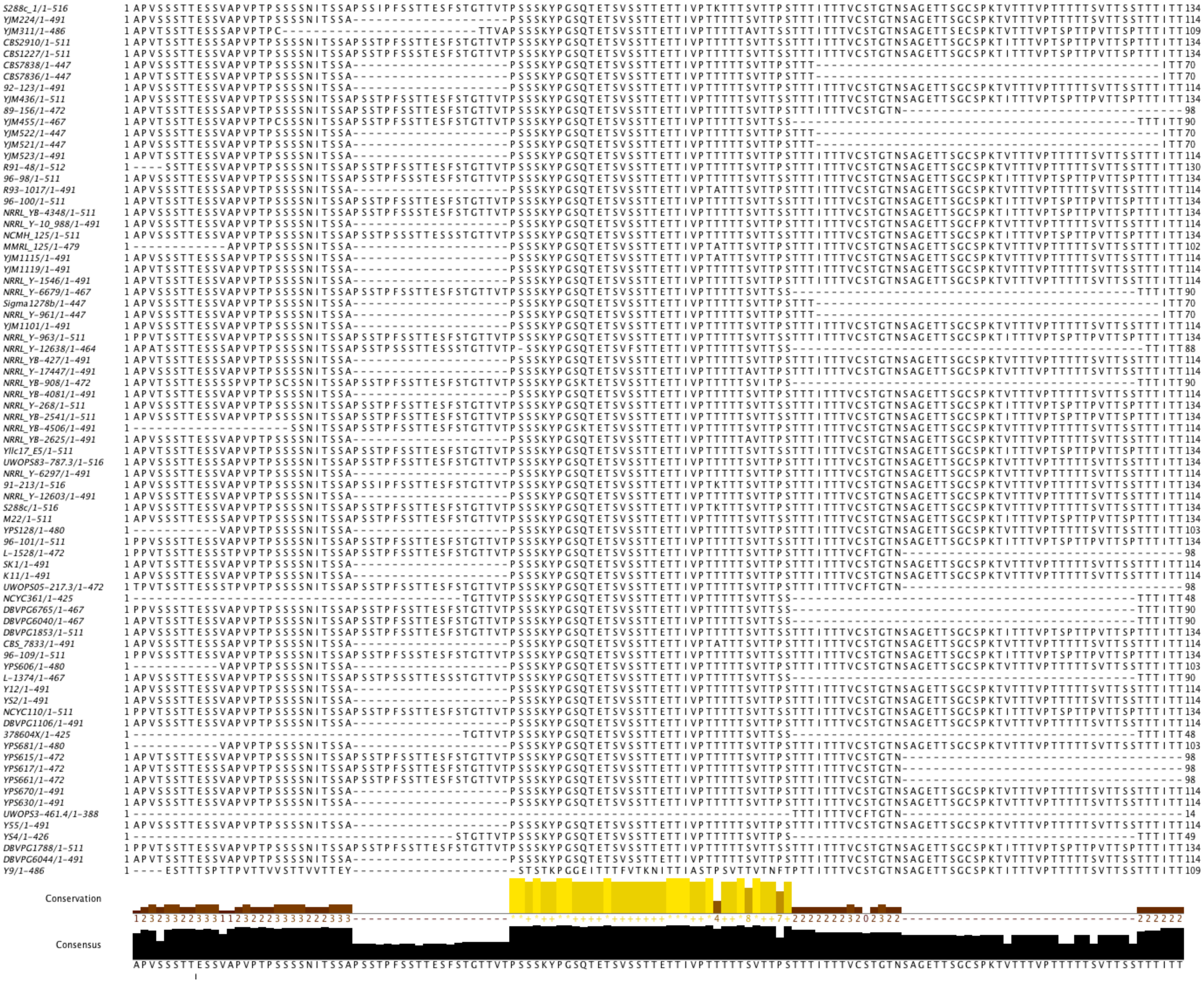

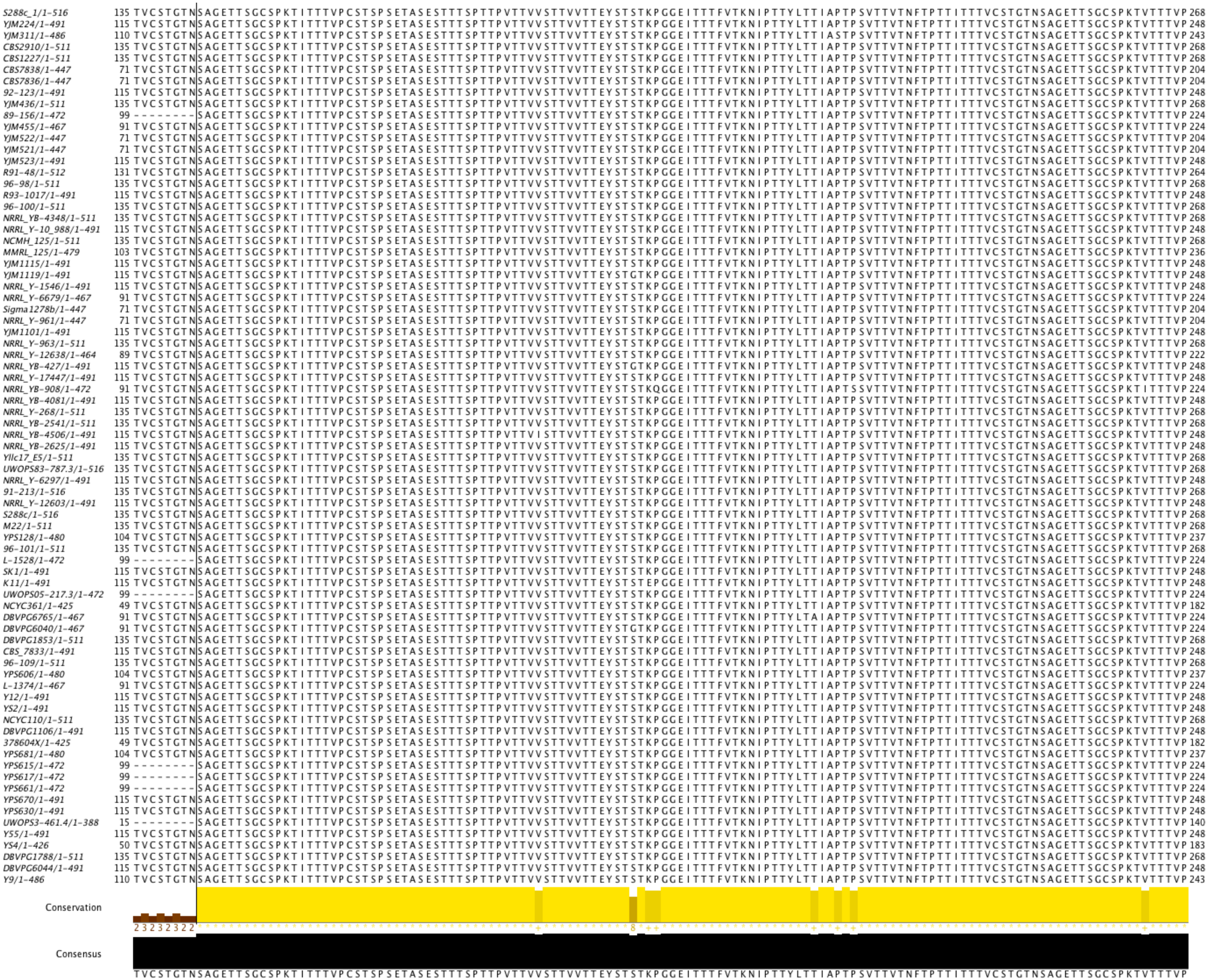
S288c_1/1-516 135 TVCSTGTNSAGETTSGCSPKTITTTVPCSTSPSETASESTTTSPTTPVTTVVSTTVVTTEYSTSTKPGGEITTTFVTKNIPTTYLTTIAPTPSVTTVTNFTPTTITTTVCSTGTNSAGETTSGCSPKTVTTTVP 268
YJM224/1-491 115 TVCSTGTNSAGETTSGCSPKTITTTVPCSTSPSETASESTTTSPTTPVTTVVSTTVVTTEYSTSTKPGGEITTTFVTKNIPTTYLTTIAPTPSVTTVTNFTPTTITTTVCSTGTNSAGETTSGCSPKTVTTTVP 248
YJM311/1-486 110 TVCSTGTNSAGETTSGCSPKTITTTVPCSTSPSETASESTTTSPTTPVTTVVSTTVVTTEYSTSTKPGGEITTTFVTKNIPTTYLTTIASTPSVTTVTNFTPTTITTTVCSTGTNSAGETTSGCSPKTVTTTVP 243
CBS2910/1-511 135 TVCSTGTNSAGETTSGCSPKTITTTVPCSTSPSETASESTTTSPTTPVTTVVSTTVVTTEYSTSTKPGGEITTTFVTKNIPTTYLTTIAPTPSVTTVTNFTPTTITTTVCSTGTNSAGETTSGCSPKTVTTTVP 268
CBS1227/1-511 135 TVCSTGTNSAGETTSGCSPKTITTTVPCSTSPSETASESTTTSPTTPVTTVVSTTVVTTEYSTSTKPGGEITTTFVTKNIPTTYLTTIAPTPSVTTVTNFTPTTITTTVCSTGTNSAGETTSGCSPKTVTTTVP 268
CBS7838/1-447 71 TVCSTGTNSAGETTSGCSPKTITTTVPCSTSPSETASESTTTSPTTPVTTVVSTTVVTTEYSTSTKPGGEITTTFVTKNIPTTYLTTIAPTPSVTTVTNFTPTTITTTVCSTGTNSAGETTSGCSPKTVTTTVP 204
CBS7836/1-447 71 TVCSTGTNSAGETTSGCSPKTITTTVPCSTSPSETASESTTTSPTTPVTTVVSTTVVTTEYSTSTKPGGEITTTFVTKNIPTTYLTTIAPTPSVTTVTNFTPTTITTTVCSTGTNSAGETTSGCSPKTVTTTVP 204
92-123/1-491 115 TVCSTGTNSAGETTSGCSPKTITTTVPCSTSPSETASESTTTSPTTPVTTVVSTTVVTTEYSTSTKPGGEITTTFVTKNIPTTYLTTIAPTPSVTTVTNFTPTTITTTVCSTGTNSAGETTSGCSPKTVTTTVP 248
YJM436/1-511 135 TVCSTGTNSAGETTSGCSPKTITTTVPCSTSPSETASESTTTSPTTPVTTVVSTTVVTTEYSTSTKPGGEITTTFVTKNIPTTYLTTIAPTPSVTTVTNFTPTTITTTVCSTGTNSAGETTSGCSPKTVTTTVP 268
89-156/1-472 99 --------SAGETTSGCSPKTITTTVPCSTSPSETASESTTTSPTTPVTTVVSTTVVTTEYSTSTKPGGEITTTFVTKNIPTTYLTTIAPTPSVTTVTNFTPTTITTTVCSTGTNSAGETTSGCSPKTVTTTVP 224
YJM455/1-467 91 TVCSTGTNSAGETTSGCSPKTITTTVPCSTSPSETASESTTTSPTTPVTTVVSTTVVTTEYSTSTKPGGEITTTFVTKNIPTTYLTTIAPTPSVTTVTNFTPTTITTTVCSTGTNSAGETTSGCSPKTVTTTVP 224
YJM522/1-447 71 TVCSTGTNSAGETTSGCSPKTITTTVPCSTSPSETASESTTTSPTTPVTTVVSTTVVTTEYSTSTKPGGEITTTFVTKNIPTTYLTTIAPTPSVTTVTNFTPTTITTTVCSTGTNSAGETTSGCSPKTVTTTVP 204
YJM521/1-447 71 TVCSTGTNSAGETTSGCSPKTITTTVPCSTSPSETASESTTTSPTTPVTTVVSTTVVTTEYSTSTKPGGEITTTFVTKNIPTTYLTTIAPTPSVTTVTNFTPTTITTTVCSTGTNSAGETTSGCSPKTVTTTVP 204
YJM523/1-491 115 TVCSTGTNSAGETTSGCSPKTITTTVPCSTSPSETASESTTTSPTTPVTTVVSTTVVTTEYSTSTKPGGEITTTFVTKNIPTTYLTTIAPTPSVTTVTNFTPTTITTTVCSTGTNSAGETTSGCSPKTVTTTVP 248
R91-48/1-512 131 TVCSTGTNSAGETTSGCSPKTITTTVPCSTSPSETASESTTTSPTTPVTTVVSTTVVTTEYSTSTKPGGEITTTFVTKNIPTTYLTTIAPTPSVTTVTNFTPTTITTTVCSTGTNSAGETTSGCSPKTVTTTVP 264
96-98/1-511 135 TVCSTGTNSAGETTSGCSPKTITTTVPCSTSPSETASESTTTSPTTPVTTVVSTTVVTTEYSTSTKPGGEITTTFVTKNIPTTYLTTIAPTPSVTTVTNFTPTTITTTVCSTGTNSAGETTSGCSPKTVTTTVP 268
R93-1017/1-491 115 TVCSTGTNSAGETTSGCSPKTITTTVPCSTSPSETASESTTTSPTTPVTTVVSTTVVTTEYSTSTKPGGEITTTFVTKNIPTTYLTTIAPTPSVTTVTNFTPTTITTTVCSTGTNSAGETTSGCSPKTVTTTVP 248
96-100/1-511 135 TVCSTGTNSAGETTSGCSPKTITTTVPCSTSPSETASESTTTSPTTPVTTVVSTTVVTTEYSTSTKPGGEITTTFVTKNIPTTYLTTIAPTPSVTTVTNFTPTTITTTVCSTGTNSAGETTSGCSPKTVTTTVP 268
NRRL_YB-4348/1-511 135 TVCSTGTNSAGETTSGCSPKTITTTVPCSTSPSETASESTTTSPTTPVTTVVSTTVVTTEYSTSTKPGGEITTTFVTKNIPTTYLTTIAPTPSVTTVTNFTPTTITTTVCSTGTNSAGETTSGCSPKTVTTTVP 268
NRRL_Y-10_988/1-491 115 TVCSTGTNSAGETTSGCSPKTITTTVPCSTSPSETASESTTTSPTTPVTTVVSTTVVTTEYSTSTKPGGEITTTFVTKNIPTTYLTTIAPTPSVTTVTNFTPTTITTTVCSTGTNSAGETTSGCSPKTVTTTVP 248
NCMH_125/1-511 135 TVCSTGTNSAGETTSGCSPKTITTTVPCSTSPSETASESTTTSPTTPVTTVVSTTVVTTEYSTSTKPGGEITTTFVTKNIPTTYLTTIAPTPSVTTVTNFTPTTITTTVCSTGTNSAGETTSGCSPKTVTTTVP 268
MMRL_125/1-479 103 TVCSTGTNSAGETTSGCSPKTITTTVPCSTSPSETASESTTTSPTTPVTTVVSTTVVTTEYSTSTKPGGEITTTFVTKNIPTTYLTTIAPTPSVTTVTNFTPTTITTTVCSTGTNSAGETTSGCSPKTVTTTVP 236
YJM1115/1-491 115 TVCSTGTNSAGETTSGCSPKTITTTVPCSTSPSETASESTTTSPTTPVTTVVSTTVVTTEYSTSTKPGGEITTTFVTKNIPTTYLTTIAPTPSVTTVTNFTPTTITTTVCSTGTNSAGETTSGCSPKTVTTTVP 248
YJM1119/1-491 115 TVCSTGTNSAGETTSGCSPKTITTTVPCSTSPSETASESTTTSPTTPVTTVVSTTVVTTEYSTGTKPGGEITTTFVTKNIPTTYLTTIAPTPSVTTVTNFTPTTITTTVCSTGTNSAGETTSGCSPKTVTTTVP 248
NRRL_Y-1546/1-491 115 TVCSTGTNSAGETTSGCSPKTITTTVPCSTSPSETASESTTTSPTTPVTTVVSTTVVTTEYSTSTKPGGEITTTFVTKNIPTTYLTTIAPTPSVTTVTNFTPTTITTTVCSTGTNSAGETTSGCSPKTVTTTVP 248
NRRL_Y-6679/1-467 91 TVCSTGTNSAGETTSGCSPKTITTTVPCSTSPSETASESTTTSPTTPVTTVVSTTVVTTEYSTSTKPGGEITTTFVTKNIPTTYLTTIAPTPSVTTVTNFTPTTITTTVCSTGTNSAGETTSGCSPKTVTTTVP 224
Sigma1278b/1-447 71 TVCSTGTNSAGETTSGCSPKTITTTVPCSTSPSETASESTTTSPTTPVTTVVSTTVVTTEYSTSTKPGGEITTTFVTKNIPTTYLTTIAPTPSVTTVTNFTPTTITTTVCSTGTNSAGETTSGCSPKTVTTTVP 204
NRRL_Y-961/1-447 71 TVCSTGTNSAGETTSGCSPKTITTTVPCSTSPSETASESTTTSPTTPVTTVVSTTVVTTEYSTSTKPGGEITTTFVTKNIPTTYLTTIAPTPSVTTVTNFTPTTITTTVCSTGTNSAGETTSGCSPKTVTTTVP 204
YJM1101/1-491 115 TVCSTGTNSAGETTSGCSPKTITTTVPCSTSPSETASESTTTSPTTPVTTVVSTTVVTTEYSTSTKPGGEITTTFVTKNIPTTYLTTIAPTPSVTTVTNFTPTTITTTVCSTGTNSAGETTSGCSPKTVTTTVP 248
NRRL_Y-963/1-511 135 TVCSTGTNSAGETTSGCSPKTITTTVPCSTSPSETASESTTTSPTTPVTTVVSTTVVTTEYSTSTKPGGEITTTFVTKNIPTTYLTTIAPTPSVTTVTNFTPTTITTTVCSTGTNSAGETTSGCSPKTVTTTVP 268
NRRL_Y-12638/1-464 89 TVCSTGTNSAGETTSGCSPKTITTTVPCSTSPSETASESTTTSPTTPVTTVVSTTVVTTEYSTSTKPGGEITTTFVTKNIPTTYLTTIAPTPSVTTVTNFTPTTITTTVCSTGTNSAGETTSGCSPKTVTTTVP 222
NRRL_YB-427/1-491 115 TVCSTGTNSAGETTSGCSPKTITTTVPCSTSPSETASESTTTSPTTPVTTVVSTTVVTTEYSTGTKPGGEITTTFVTKNIPTTYLTTIAPTPSVTTVTNFTPTTITTTVCSTGTNSAGETTSGCSPKTVTTTVP 248
NRRL_Y-17447/1-491 115 TVCSTGTNSAGETTSGCSPKTITTTVPCSTSPSETASESTTTSPTTPVTTVVSTTVVTTEYSTSTKPGGEITTTFVTKNIPTTYLTTIAPTPSVTTVTNFTPTTITTTVCSTGTNSAGETTSGCSPKTVTTTVP 248
NRRL_YB-908/1-472 91 TVCSTGTNSAGETTSGCSPKTITTTVPCSTSPSETASESTTTSPTTPVTTVVSTTVVTTEYSTSTKQGGEITTTFVTKNIPTTYLTTIAPTSSVTTVTNFTPTTITTTVCSTGTNSAGETTSGCSPKTITTTVP 224
NRRL_YB-4081/1-491 115 TVCSTGTNSAGETTSGCSPKTITTTVPCSTSPSETASESTTTSPTTPVTTVVSTTVVTTEYSTSTKPGGEITTTFVTKNIPTTYLTTIAPTPSVTTVTNFTPTTITTTVCSTGTNSAGETTSGCSPKTVTTTVP 248
NRRL_Y-268/1-511 135 TVCSTGTNSAGETTSGCSPKTITTTVPCSTSPSETASESTTTSPTTPVTTVVSTTVVTTEYSTSTKPGGEITTTFVTKNIPTTYLTTIAPTPSVTTVTNFTPTTITTTVCSTGTNSAGETTSGCSPKTVTTTVP 268
NRRL_YB-2541/1-511 135 TVCSTGTNSAGETTSGCSPKTITTTVPCSTSPSETASESTTTSPTTPVTTVVSTTVVTTEYSTSTKPGGEITTTFVTKNIPTTYLTTIAPTPSVTTVTNFTPTTITTTVCSTGTNSAGETTSGCSPKTVTTTVP 268
NRRL_YB-4506/1-491 115 TVCSTGTNSAGETTSGCSPKTITTTVPCSTSPSETASESTTTSPTTPVTTVISTTVVTTEYSTSTKPGGEITTTFVTKNIPTTYLTTIAPTPSVTTVTNFTPTTITTTVCSTGTNSAGETTSGCSPKTVTTTVP 248
NRRL_YB-2625/1-491 115 TVCSTGTNSAGETTSGCSPKTITTTVPCSTSPSETASESTTTSPTTPVTTVVSTTVVTTEYSTSTKPGGEITTTFVTKNIPTTYLTTIAPTPSVTTVTNFTPTTITTTVCSTGTNSAGETTSGCSPKTVTTTVP 248
Yllc17_E5/1-511 135 TVCSTGTNSAGETTSGCSPKTITTTVPCSTSPSETASESTTTSPTTPVTTVVSTTVVTTEYSTSTKPGGEITTTFVTKNIPTTYLTTIAPTPSVTTVTNFTPTTITTTVCSTGTNSAGETTSGCSPKTVTTTVP 268
UWOPS83-787.3/1-516 135 TVCSTGTNSAGETTSGCSPKTITTTVPCSTSPSETASESTTTSPTTPVTTVVSTTVVTTEYSTSTKPGGEITTTFVTKNIPTTYLTTIAPTPSVTTVTNFTPTTITTTVCSTGTNSAGETTSGCSPKTVTTTVP 268
NRRL_Y-6297/1-491 115 TVCSTGTNSAGETTSGCSPKTITTTVPCSTSPSETASESTTTSPTTPVTTVVSTTVVTTEYSTSTKPGGEITTTFVTKNIPTTYLTTIAPTPSVTTVTNFTPTTITTTVCSTGTNSAGETTSGCSPKTVTTTVP 248
91-213/1-516 135 TVCSTGTNSAGETTSGCSPKTITTTVPCSTSPSETASESTTTSPTTPVTTVVSTTVVTTEYSTSTKPGGEITTTFVTKNIPTTYLTTIAPTPSVTTVTNFTPTTITTTVCSTGTNSAGETTSGCSPKTVTTTVP 268
NRRL_Y-12603/1-491 115 TVCSTGTNSAGETTSGCSPKTITTTVPCSTSPSETASESTTTSPTTPVTTVVSTTVVTTEYSTSTKPGGEITTTFVTKNIPTTYLTTIAPTPSVTTVTNFTPTTITTTVCSTGTNSAGETTSGCSPKTVTTTVP 248
S288c/1-516 135 TVCSTGTNSAGETTSGCSPKTITTTVPCSTSPSETASESTTTSPTTPVTTVVSTTVVTTEYSTSTKPGGEITTTFVTKNIPTTYLTTIAPTPSVTTVTNFTPTTITTTVCSTGTNSAGETTSGCSPKTVTTTVP 268
M22/1-511 135 TVCSTGTNSAGETTSGCSPKTITTTVPCSTSPSETASESTTTSPTTPVTTVVSTTVVTTEYSTSTKPGGEITTTFVTKNIPTTYLTTIAPTPSVTTVTNFTPTTITTTVCSTGTNSAGETTSGCSPKTVTTTVP 268
YPS128/1-480 104 TVCSTGTNSAGETTSGCSPKTITTTVPCSTSPSETASESTTTSPTTPVTTVVSTTVVTTEYSTSTKPGGEITTTFVTKNIPTTYLTTIAPTPSVTTVTNFTPTTITTTVCSTGTNSAGETTSGCSPKTVTTTVP 237
96-101/1-511 135 TVCSTGTNSAGETTSGCSPKTITTTVPCSTSPSETASESTTTSPTTPVTTVVSTTVVTTEYSTSTKPGGEITTTFVTKNIPTTYLTTIAPTPSVTTVTNFTPTTITTTVCSTGTNSAGETTSGCSPKTVTTTVP 268
L-1528/1-472 99 --------SAGETTSGCSPKTITTTVPCSTSPSETASESTTTSPTTPVTTVVSTTVVTTEYSTSTKPGGEITTTFVTKNIPTTYLTTIAPTPSVTTVTNFTPTTITTTVCSTGTNSAGETTSGCSPKTVTTTVP 224
SK1/1-491 115 TVCSTGTNSAGETTSGCSPKTITTTVPCSTSPSETASESTTTSPTTPVTTVVSTTVVTTEYSTSTKPGGEITTTFVTKNIPTTYLTTIAPTPSVTTVTNFTPTTITTTVCSTGTNSAGETTSGCSPKTVTTTVP 248
K11/1-491 115 TVCSTGTNSAGETTSGCSPKTITTTVPCSTSPSETASESTTTSPTTPVTTVVSTTVVTTEYSTSTEPGGEITTTFVTKNIPTTYLTTIAPTPSVTTVTNFTPTTITTTVCSTGTNSAGETTSGCSPKTVTTTVP 248
UWOPS05-217.3/1-472 99 --------SAGETTSGCSPKTITTTVPCSTSPSETASESTTTSPTTPVTTVVSTTVVTTEYSTSTKPGGEITTTFVTKNIPTTYLTTIAPTPSVTTVTNFTPTTITTTVCSTGTNSAGETTSGCSPKTVTTTVP 224
NCYC361/1-425 49 TVCSTGTNSAGETTSGCSPKTITTTVPCSTSPSETASESTTTSPTTPVTTVVSTTVVTTEYSTSTKPGGEITTTFVTKNIPTTYLTTIAPTPSVTTVTNFTPTTITTTVCSTGTNSAGETTSGCSPKTVTTTVP 182
DBVPG6765/1-467 91 TVCSTGTNSAGETTSGCSPKTITTTVPCSTSPSETASESTTTSPTTPVTTVVSTTVVTTEYSTSTKPGGEITTTFVTKNIPTTYLTAIAPTPSVTTVTNFTPTTITTTVCSTGTNSAGETTSGCSPKTVTTTVP 224
DBVPG6040/1-467 91 TVCSTGTNSAGETTSGCSPKTITTTVPCSTSPSETASESTTTSPTTPVTTVVSTTVVTTEYSTGTKPGGEITTTFVTKNIPTTYLTTIAPTPSVTTVTNFTPTTITTTVCSTGTNSAGETTSGCSPKTVTTTVP 224
DBVPG1853/1-511 135 TVCSTGTNSAGETTSGCSPKTITTTVPCSTSPSETASESTTTSPTTPVTTVVSTTVVTTEYSTSTKPGGEITTTFVTKNIPTTYLTTIAPTPSVTTVTNFTPTTITTTVCSTGTNSAGETTSGCSPKTVTTTVP 268
CBS_7833/1-491 115 TVCSTGTNSAGETTSGCSPKTITTTVPCSTSPSETASESTTTSPTTPVTTVVSTTVVTTEYSTSTKPGGEITTTFVTKNIPTTYLTTIAPTPSVTTVTNFTPTTITTTVCSTGTNSAGETTSGCSPKTVTTTVP 248
96-109/1-511 135 TVCSTGTNSAGETTSGCSPKTITTTVPCSTSPSETASESTTTSPTTPVTTVVSTTVVTTEYSTSTKPGGEITTTFVTKNIPTTYLTTIAPTPSVTTVTNFTPTTITTTVCSTGTNSAGETTSGCSPKTVTTTVP 268
YPS606/1-480 104 TVCSTGTNSAGETTSGCSPKTITTTVPCSTSPSETASESTTTSPTTPVTTVVSTTVVTTEYSTSTKPGGEITTTFVTKNIPTTYLTTIAPTPSVTTVTNFTPTTITTTVCSTGTNSAGETTSGCSPKTVTTTVP 237
L-1374/1-467 91 TVCSTGTNSAGETTSGCSPKTITTTVPCSTSPSETASESTTTSPTTPVTTVVSTTVVTTEYSTSTKPGGEITTTFVTKNIPTTYLTTIAPTPSVTTVTNFTPTTITTTVCSTGTNSAGETTSGCSPKTVTTTVP 224
Y12/1-491 115 TVCSTGTNSAGETTSGCSPKTITTTVPCSTSPSETASESTTTSPTTPVTTVVSTTVVTTEYSTSTKPGGEITTTFVTKNIPTTYLTTIAPTPSVTTVTNFTPTTITTTVCSTGTNSAGETTSGCSPKTVTTTVP 248
YS2/1-491 115 TVCSTGTNSAGETTSGCSPKTITTTVPCSTSPSETASESTTTSPTTPVTTVVSTTVVTTEYSTSTKPGGEITTTFVTKNIPTTYLTTIAPTPSVTTVTNFTPTTITTTVCSTGTNSAGETTSGCSPKTVTTTVP 248
NCYC110/1-511 135 TVCSTGTNSAGETTSGCSPKTITTTVPCSTSPSETASESTTTSPTTPVTTVVSTTVVTTEYSTSTKPGGEITTTFVTKNIPTTYLTTIAPTPSVTTVTNFTPTTITTTVCSTGTNSAGETTSGCSPKTVTTTVP 268
DBVPG1106/1-491 115 TVCSTGTNSAGETTSGCSPKTITTTVPCSTSPSETASESTTTSPTTPVTTVVSTTVVTTEYSTSTKPGGEITTTFVTKNIPTTYLTTIAPTPSVTTVTNFTPTTITTTVCSTGTNSAGETTSGCSPKTVTTTVP 248
378604X/1-425 49 TVCSTGTNSAGETTSGCSPKTITTTVPCSTSPSETASESTTTSPTTPVTTVVSTTVVTTEYSTSTKPGGEITTTFVTKNIPTTYLTTIAPTPSVTTVTNFTPTTITTTVCSTGTNSAGETTSGCSPKTVTTTVP 182
YPS681/1-480 104 TVCSTGTNSAGETTSGCSPKTITTTVPCSTSPSETASESTTTSPTTPVTTVVSTTVVTTEYSTSTKPGGEITTTFVTKNIPTTYLTTIAPTPSVTTVTNFTPTTITTTVCSTGTNSAGETTSGCSPKTVTTTVP 237
YPS615/1-472 99 --------SAGETTSGCSPKTITTTVPCSTSPSETASESTTTSPTTPVTTVVSTTVVTTEYSTSTKPGGEITTTFVTKNIPTTYLTTIAPTPSVTTVTNFTPTTITTTVCSTGTNSAGETTSGCSPKTVTTTVP 224
YPS617/1-472 99 --------SAGETTSGCSPKTITTTVPCSTSPSETASESTTTSPTTPVTTVVSTTVVTTEYSTSTKPGGEITTTFVTKNIPTTYLTTIAPTPSVTTVTNFTPTTITTTVCSTGTNSAGETTSGCSPKTVTTTVP 224
YPS661/1-472 99 --------SAGETTSGCSPKTITTTVPCSTSPSETASESTTTSPTTPVTTVVSTTVVTTEYSTSTKPGGEITTTFVTKNIPTTYLTTIAPTPSVTTVTNFTPTTITTTVCSTGTNSAGETTSGCSPKTVTTTVP 224
YPS670/1-491 115 TVCSTGTNSAGETTSGCSPKTITTTVPCSTSPSETASESTTTSPTTPVTTVVSTTVVTTEYSTSTKPGGEITTTFVTKNIPTTYLTTIAPTPSVTTVTNFTPTTITTTVCSTGTNSAGETTSGCSPKTVTTTVP 248
YPS630/1-491 115 TVCSTGTNSAGETTSGCSPKTITTTVPCSTSPSETASESTTTSPTTPVTTVVSTTVVTTEYSTSTKPGGEITTTFVTKNIPTTYLTTIAPTPSVTTVTNFTPTTITTTVCSTGTNSAGETTSGCSPKTVTTTVP 248
UWOPS3-461.4/1-388 15 --------SAGETTSGCSPKTITTTVPCSTSPSETASESTTTSPTTPVTTVVSTTVVTTEYSTSTKPGGEITTTFVTKNIPTTYLTTIAPTPSVTTVTNFTPTTITTTVCSTGTNSAGETTSGCSPKTVTTTVP 140
Y55/1-491 115 TVCSTGTNSAGETTSGCSPKTITTTVPCSTSPSETASESTTTSPTTPVTTVVSTTVVTTEYSTSTKPGGEITTTFVTKNIPTTYLTTIAPTPSVTTVTNFTPTTITTTVCSTGTNSAGETTSGCSPKTVTTTVP 248
YS4/1-426 50 TVCSTGTNSAGETTSGCSPKTITTTVPCSTSPSETASESTTTSPTTPVTTVVSTTVVTTEYSTSTKPGGEITTTFVTKNIPTTYLTTIAPTPSVTTVTNFTPTTITTTVCSTGTNSAGETTSGCSPKTVTTTVP 183
DBVPG1788/1-511 135 TVCSTGTNSAGETTSGCSPKTITTTVPCSTSPSETASESTTTSPTTPVTTVVSTTVVTTEYSTSTKPGGEITTTFVTKNIPTTYLTTIAPTPSVTTVTNFTPTTITTTVCSTGTNSAGETTSGCSPKTVTTTVP 268
DBVPG6044/1-491 115 TVCSTGTNSAGETTSGCSPKTITTTVPCSTSPSETASESTTTSPTTPVTTVVSTTVVTTEYSTSTKPGGEITTTFVTKNIPTTYLTTIAPTPSVTTVTNFTPTTITTTVCSTGTNSAGETTSGCSPKTVTTTVP 248
Y9/1-486 110 TVCSTGTNSAGETTSGCSPKTITTTVPCSTSPSETASESTTTSPTTPVTTVVSTTVVTTEYSTSTKPGGEITTTFVTKNIPTTYLTTIAPTPSVTTVTNFTPTTITTTVCSTGTNSAGETTSGCSPKTVTTTVP 243
Conservation
2 3 2 3 2 3 2 2
8
Consensus
TVCSTGTNSAGETTSGCSPKTITTTVPCSTSPSETASESTTTSPTTPVTTVVSTTVVTTEYSTSTKPGGEITTTFVTKNIPTTYLTTIAPTPSVTTVTNFTPTTITTTVCSTGTNSAGETTSGCSPKTVTTTVP

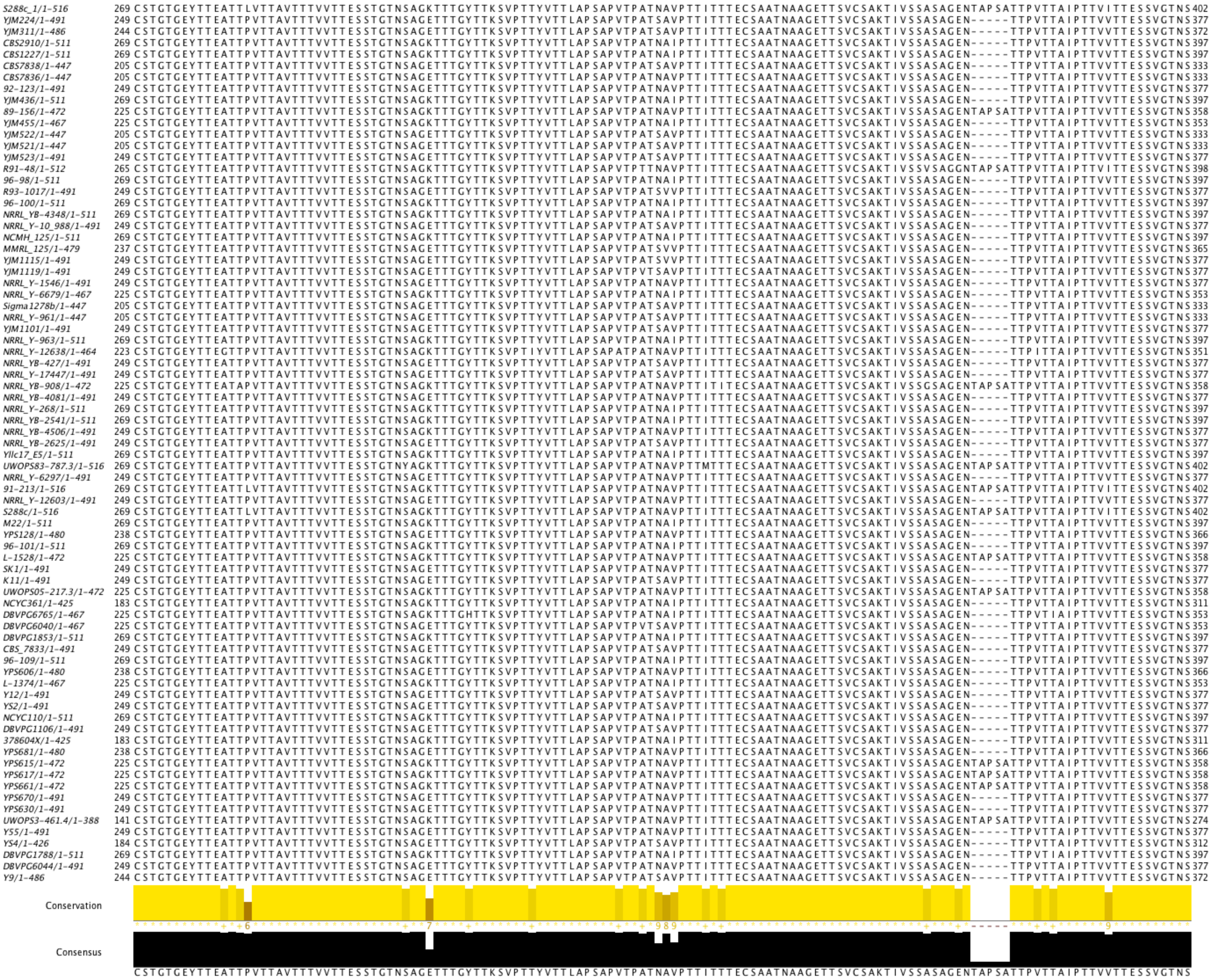
Conservation
Consensus
CSTGTGEYTTEATTPVTTAVTTTVVTTESSTGTNSAGETTTGYTTKSVPTTYVTTLAPSAPVTPATNAVPTTITTTECSAATNAAGETTSVCSAKTIVSSASAGENTAPSATTPVTTAIPTTVVTTESSVGTNS

S288c_1/1-516	403	AGETTTGYTTKSIPTTYITTLIPGSNGAKNYETVATATNPISIKTTSQLATTASASSVAPVVTSPSLTGPLQSASGSAVATYSVPSISSTYQGAANIKVLGNFMWLLLALPVVF	516
YJM224/1-491	378	AGETTTGYTTKSIPTTYITTLIPGSNGAKNYETVATATNPISIKTTSQLATTASASSMAPVVTSPSLTGPLQSASGSAVATYSVPSISSTYQGAANIKVLGNFMWLLLALPVVF	491
YJM311/1-486	373	AGETTTGYTTKSIPTTYITTLIPGSNGAKNYETVATATNPISIKTTSQLATTASASSMAPVVTSPSLTGPLQSASGSAVATYSVPSISSTYQGAANIKVLGNFMWLLLALPVVF	486
CBS2910/1-511	398	AGETTTGYTTKSIPTTYITTLIPGSNGAKNYETVATATNPISIKTTSQLATTASASSMAPVVTSPSLTGPLQSASGSAVATYSVPSISSTYQGAANIKVLGNFMWLLLALPVVF	511
CBS1227/1-511	398	AGETTTGYTTKSIPTTYITTLIPGSNGAKNYETVATATNPISIKTTSQLATTASASSMAPVVTSPSLTGPLQSASGSAVATYSVPSISSTYQGAANIKVLGNFMWLLLALPVVF	511
CBS7838/1-447	334	AGETTTGYTTKSIPTTYITTLIPGSNGAKNYETVATATNPISIKTTSQLATTASASSMAPVVTSPSLTGPLQSASGSAVATYSVPSISSTYQGAANIKVLGNFMWLLLALPVVF	447
CBS7836/1-447	334	AGETTTGYTTKSIPTTYITTLIPGSNGAKNYETVATATNPISIKTTSQLATTASASSMAPVVTSPSQTGPLQSASGSAVATYSVPSISSTYQGAANIKVLGNFMWLLLALPVVF	447
92-123/1-491	378	AGETTTGYTTKSIPTTYITTLIPGSNGAKNYETVATATNPISIKTTSQLATTASASSMAPVVTSPSLTGPLQSASGSAVATYSVPSISSTYQGAANIKVLGNFMWLLLALPVVF	491
YJM436/1-511	398	AGETTTGYTTKSIPTTYITTLIPGSNGAKNYETVATATNPISIKTTSQLATTASASSMAPVVTSPSLTGPLQSASGSAVATYSVPSISSTYQGAANIKVLGNFMWLLLALPVVF	511
89-156/1-472	359	AGETTTGYTTKSIPTTYITTLIPGSNGAKNYETVATATNPISIKTTSQLATTASASSMAPVVTSPSLTGPLQSASGSAVATYSVPSISSTYQGAANIKVLGNFMWLLLALPVVV	472
YJM455/1-467	354	AGETTTGYTTKSIPTTYITTLIPGSNGAKNYETVATATNPISIKTTSQLATTASASSMAPVVTSPSLTGPLQSASGSAVATYSVPSISSTYQGAANIKVLGNFMWLLLALPVVF	467
YJM522/1-447	334	AGETTTGYTTKSIPTTYITTLIPGSNGAKNYETVATATNPISIKTTSQLATTASASSMAPVVTSPSLTGPLQSASGSAVATYSVPSISSTYQGAANIKVLGNFMWLLLALPVVF	447
YJM521/1-447	334	AGETTTGYTTKSIPTTYITTLIPGSNGAKNYETVATATNPISIKTTSQLATTASASSMAPVVTSPSLTGPLQSASGSAVATYSVPSISSTYQGAANIKVLGNFMWLLLALPVVF	447
YJM523/1-491	378	AGETTTGYTTKSIPTTYITTLIPGSNGAKNYETVATATNPISIKTTSQLATTASASSMAPVVTSPSLTGPLQSASGSAVATYSVPSISSTYQGAANIKVLGNFMWLLLALPVVF	491
R91-48/1-512	399	AGETTTGYTTKSIPTTYITTLIPGSNGAKNYETVATATNPISIKTTSQLATTASASSVAPVVTSPSLTGPLQSASGSAVATYSVPSISSTYQGAANIKVLGNFMWLLLALPVVF	512
96-98/1-511	398	AGETTTGYTTKSIPTTYITTLIPGSNGAKNYETVATATNPISIKTTSQLATTASASSMAPVVTSPSLTGPLQSASGSAVATYSVPSISSTYQGAANIKVLGNFMWLLLALPVVF	511
R93-1017/1-491	378	AGETTTGYTTKSIPTTYITTLIPGSNGAKNYETVATATNPISIKTTSQLATTASASSMAPVVTSPSLTGPLQSASGSAVATYSVPSISSTYQGAANIKVLGNFMWLLLALPVVF	491
96-100/1-511	398	AGETTTGYTTKSIPTTYITTLIPGSNGAKNYETVATATNPISIKTTSQLATTASASSMAPVVTSPSLTGPLQSASGSAVATYSVPSISSTYQGAANIKVLGNFMWLLLALPVVF	511
NRRL_YB-4348/1-511	398	AGETTTGYTTKSIPTTYITTLIPGSNGAKNYETVATATNPISIKTTSQLATTASASSMAPVVTSPSLTGPLQSASGSAVATYSVPSISSTYQGAANIKVLGNFMWLLLALPVVF	511
NRRL_Y-10_988/1-491	378	AGETTTGYTTKSIPTTYITTLIPGSNGAKNYETVATATNPISIKTTSQLATTASASSMAPVVTSPSLTGPLQSASGSAVATYSVPSISSTYQGAANIKVLGNFMWLLLALPVVF	491
NCMH_125/1-511	398	AGETTTGYTTKSIPTTYITTLIPGSNGAKNYETVATATNPISIKTTSQLATTASASSMAPVVTSPSLTGPLQSASGSAVATYSVPSISSTYQGAANIKVLGNFMWLLLALPVVF	511
MMRL_125/1-479	366	AGETTTGYTTKSIPTTYITTLIPGSNGAKNYETVATATNPISIKTTSQLATTASASSMAPVVTSPSLTGPLQSASGSAVATYSVPSISSTYQGAANIKVLGNFMWLLLALPVVF	479
YJM1115/1-491	378	AGETTTGYTTKSIPTTYITTLIPGSNGAKNYETVATATNPISIKTTSQLATTASASSMAPVVTSPSLTGPLQSASGSAVATYSVPSISSTYQGAANIKVLGNFMWLLLALPVVF	491
YJM1119/1-491	378	AGETTTGYTTKSIPTTYITTLIPGSNGAKNYETVATATNPISIKTTSQLATTASASSMAPVVTSPSLTGPLQSASGSAVATYSVPSISSTYQGAANIKVLGNFMWLLLALPVVF	491
NRRL_Y-1546/1-491	378	AGETTTGYTTKSIPTTYITTLIPGSNGAKNYETVATATNPISIKTTSQLATTASASSMAPVVTSPSLTGPLQSASGSAVATYSVPSISSTYQGAANIKVLGNFMWLLLALPVVF	491
NRRL_Y-6679/1-467	354	AGETTTGYTTKSIPTTYITTLIPGSNGAKNYETVATATNPISIKTTSQLATTASGSSMAPVVTSPSLTGPVQSASGPAVATYSAPAISSTYQGAANIKVLGNFMWLLVALPIVI	467
Sigma1278b/1-447	334	AGETTTGYTTKSIPTTYITTLIPGSNGAKNYETVATATNPISIKTTSQLATTASASSMAPVVTSPSLTGPLQSASGSAVATYSVPSISSTYQGAANIKVLGNFMWLLLALPVVF	447
NRRL_Y-961/1-447	334	AGETTTGYTTKSIPTTYITTLIPGSNGAKNYETVATATNPISIKTTSQLATTASASSMAPVVTSPSLTGPLQSASGSAVATYSVPSISSTYQGAANIKVLGNFMWLLLALPVVF	447
YJM1101/1-491	378	AGETTTGYTTKSIPTTYITTLIPGSNGAKNYETVATATNPISIKTTSQLATTASASSMAPVVTSPSLTGPLQSASGSAVATYSVPSISSTYQGAANIKVLGNFMWLLLALPVVF	491
NRRL_Y-963/1-511	398	AGETTTGYTTKSIPTTYITTLIPGSNGAKNYETVATATNPISIKTTSQLATTASASSMAPVVTSPSLTGPLQSASGSAVATYSVPSISSTYQGAANIKVLGNFMWLLLALPVVF	511
NRRL_Y-12638/1-464	352	AGETTTGYTTKSIPTTYITTLIPGSNGAKNYETVATATNPISIKTTSQLTTTASASSMAPVVTSPSLTGPLQS-SGSAVATYSVPSISSTYQGAANIKVLGNFMWLLLALPVVF	464
NRRL_YB-427/1-491	378	AGETTTGYTTKSIPTTYITTLIPGSNGAKNYETVATATNPISIKTTSQLATTASASSMAPVVTSPSLTGPLQSASGSAVATYSVPSISSTYQGAANIKVLGNFMWLLLALPVVF	491
NRRL_Y-17447/1-491	378	AGETTTGYTTKSIPTTYITTLIPGSNGAKNYETVATATNPISIKTTSQLATTASASSMAPVVTSPSLTGPLQSASGSAVATYSVPSISSTYQGAANIKVLGNFMWLLLALPVVF	491
NRRL_YB-908/1-472	359	AGETTTGYTTKSIPTTYITTLIPGSNGAKNYETVATATNPISIKTTSQLATTASASSVAPVVTSPSLTGPLQSASGSAVATYSVPSISSTYQGAANIKVLGNFMWLLLALPVVV	472
NRRL_YB-4081/1-491	378	AGETTTGYTTKSIPTTYITTLIPGSNGAKNYETVATATNPISIKTTSQLATTASASSMAPVVTSPSLTGPLQSASGSAVATYSVPSISSTYQGAANIKVLGNFMWLLLALPVVF	491
NRRL_Y-268/1-511	398	AGETTTGYTTKSIPTTYITTLIPGSNGAKNYETVATATNPISIKTTSQLATTASASSMAPVVTSPSLTGPLQSASGSAVATYSVPSISSTYQGAANIKVLGNFMWLLLALPVVF	511
NRRL_YB-2541/1-511	398	AGETTTGYTTKSIPTTYITTLIPGSNGAKNYETVATATNPISIKTTSQLATTASASSMAPVVTSPSLTGPLQSASGSAVATYSVPSISSTYQGAANIKVLGNFMWLLLALPVVF	511
NRRL_YB-4506/1-491	378	AGETTTGYTTKSIPTTYITTLIPGSNGAKNYETVATATNPISIKTTSQLATTASASSMAPVVTSPSLTGPLQSASGSAVATYSVPSISSTYQGAANIKVLGNFMWLLLALPVVF	491
NRRL_YB-2625/1-491	378	AGETTTGYTTKSIPTTYITTLIPGSNGAKNYETVATATNPISIKTTSQLATTASASSMAPVVTSPSLTGPLQSASGSAVATYSVPSISSTYQGAANIKVLGNFMWLLLALPVVF	491
Yllc17_E5/1-511	398	AGETTTGYTTKSIPTTYITTLIPGSNGAKNYETVATATNPISIKTTSQLATTASASSMAPVVTSPSLTGPLQSASGSAVATYSVPSISSTYQGAANIKVLGNFMWLLLALPVVF	511
UWOPS83-787.3/1-516	403	AGETTTGYTTKSIPTTYVTTLIPGSNGAKNYETVATATNPISIKTTSQLATTASASSMAPVVTSPSLTGPLQSASGSAVATYSVPSISSTYQGAANIKVLGNFMWLLLALPVVV	516
NRRL_Y-6297/1-491	378	AGETTTGYTTKSIPTTYITTLIPGSNGAKNYETVATATNPISIKTTSQLATTASASSMAPVVTSPSLTGPLQSASGSAVATYSVPSISSTYQGAANIKVLGNFMWLLLALPVVF	491
91-213/1-516	403	AGETTTGYTTKSIPTTYITTLIPGSNGAKNYETVATATNPISIKTTSQLATTASASSVAPVVTSPSLTGPLQSASGSAVATYSVPSISSTYQGAANIKVLGNFMWLLLALPVVF	516
NRRL_Y-12603/1-491	378	AGETTTGYTTKSIPTTYITTLIPGSNGAKNYETVATATNPISIKTTSQLATTASASSMAPVVTSPSLTGPLQSASGSAVATYSVPSISSTYQGAANIKVLGNFMWLLLALPVVF	491
S288c/1-516	403	AGETTTGYTTKSIPTTYITTLIPGSNGAKNYETVATATNPISIKTTSQLATTASASSVAPVVTSPSLTGPLQSASGSAVATYSVPSISSTYQGAANIKVLGNFMWLLLALPVVF	516
M22/1-511	398	AGETTTGYTTKSIPTTYITTLIPGSNGAKNYETVATATNPISIKTTSQLATTASASSMAPVVTSPSLTGPLQSASGSAVATYSVPSISSTYQGAANIKVLGNFMWLLLALPVVF	511
YPS128/1-480	367	AGETTTGYTTKSIPTTYITTLIPGSNGAKNYETVATATNPISIKTTSQLATTASASSMAPVVTSPSLTGPLQSASGSAVATYSVPSISSTYQGAANIKVLGNFMWLLLALPVVF	480
96-101/1-511	398	AGETTTGYTTKSIPTTYITTLIPGSNGAKNYETVATATNPISIKTTSQLATTASASSMAPVVTSPSLTGPLQSASGSAVATYSVPSISSTYQGAANIKVLGNFMWLLLALPVVF	511
L-1528/1-472	359	AGETTTGYTTKSIPTTYITTLIPGSNGAKNYETVATATNPISIKTTSQLATTASASSMAPVVTSPSLTGPLQSASGSAVATYSVPSISSTYQGAANIKVLGNFMWLLLALPVVV	472
SK1/1-491	378	AGETTTGYTTKSIPTTYITTLIPGSNGAKNYETVATATNPISIKTTSQLATTASASSMAPVVTSPSLTGPLQSASGSAVATYSVPSISSTYQGAANIKVLGNFMWLLLALPVVF	491
K11/1-491	378	AGETTTGYTTKSIPTTYITTLIPGSNGAKNYETVATATNPISIKTTSQLATTASASSMAPVVTSPSLTGPLQSASGSAVATYSVPSISSTYQGAANIKVLGNFMWLLLALPVVF	491
UWOPS05-217.3/1-472	359	AGETTTGYTTKSIPTTYITTLIPGSNGAKNYETVATATNPISIKTTSQLATTASASSMAPVVTSPSLTGPLQSASGSAVATYSVPSISSTYQGAANIKVLGNFMWLLLALPVVV	472
NCYC361/1-425	312	AGETTTGYTTKSIPTTYITTLIPGSNGAKNYETVATATNPISIKTTSQLATTASASSMAPVVTSPSLTGPLQSASGSAVATYSVPSISSTYQGAANIKVLGNFMWLLLALPVVF	425
DBVPG6765/1-467	354	AGETTTGYTTKSIPTTYITTLIPGSNGAKNYETVATATNPISIKTTSQLATTASASSMAPVVTSPSLTGPLQSASGSAVATYSVPSISSTYQGAANIKVLGNFMWLLLALPVVF	467
DBVPG6040/1-467	354	AGETTTGYTTKSIPTTYITTLIPGSNGAKNYETVATATNPISIKTTSQLATTASASSMAPVVTSPSLTGPLQSASGSAVATYSVPSISSTYQGAANIKVLGNFMWLLLALPVVF	467
DBVPG1853/1-511	398	AGETTTGYTTKSIPTTYITTLIPGSNGAKNYETVATATNPISIKTTSQLATTASASSMAPVVTSPSLTGPLQSASGSAVATYSVPSISSTYQGAANIKVLGNFMWLLLALPVVF	511
CBS_7833/1-491	378	AGETTTGYTTKSIPTTYITTLIPGSNGAKNYETVATATNPISIKTTSQLATTASASSMAPVVTSPSLTGPLQSASGSAVATYSVPSISSTYQGAANIKVLGNFMWLLLALPVVF	491
96-109/1-511	398	AGETTTGYTTKSIPTTYITTLIPGSNGAKNYETVATATNPISIKTTSQLATTASASSMAPVVTSPSLTGPLQSASGSAVATYSVPSISSTYQGAANIKVLGNFMWLLLALPVVF	511
YPS606/1-480	367	AGETTTGYTTKSIPTTYITTLIPGSNGAKNYETVATATNPISIKTTSQLATTASASSMAPVVTSPSLTGPLQSASGSAVATYSVPSISSTYQGAANIKVLGNFMWLLLALPVVF	480
L-1374/1-467	354	AGETTTGYTTKSIPTTYITTLIPGSNGAKNYETVATATNPISIKTTSQLATTASASSMAPVVTSPSLTGPLQSASGSAVATYSVPSISSTYQGAANIKVLGNFMWLLLALPVVF	467
Y12/1-491	378	AGETTTGYTTKSIPTTYITTLIPGSNGAKNYETVATATNPISIKTTSQLATTASASSMAPVVTSPSLTGPLQSASGSAVATYSVPSISSTYQGAANIKVLGNFMWLLLALPVVF	491
YS2/1-491	378	AGETTTGYTTKSIPTTYITTLIPGSNGAKNYETVATATNPISIKTTSQLATTASASSMAPVVTSPSLTGPLQSASGSAVATYSVPSISSTYQGAANIKVLGNFMWLLLALPVVF	491
NCYC110/1-511	398	AGETTTGYTTKSIPTTYITTLIPGSNGAKNYETVATATNPISIKTTSQLATTASASSMAPVVTSPSLTGPLQSASGSAVATYSVPSISSTYQGAANIKVLGNFMWLLLALPVVF	511
DBVPG1106/1-491	378	AGETTTGYTTKSIPTTYITTLIPGSNGAKNYETVATATNPISIKTTSQLATTASASSMAPVVTSPSLTGPLQSASGSAVATYSVPSISSTYQGAANIKVLGNFMWLLLALPVVF	491
378604X/1-425	312	AGETTTGYTTKSIPTTYITTLIPGSNGAKNYETVATATNPISIKTTSQLATTASASSMAPVVTSPSLTGPLQSASGSAVATYSVPSISSTYQGAANIKVLGNFMWLLLALPVVF	425
YPS681/1-480	367	AGETTTGYTTKSIPTTYITTLIPGSNGAKNYETVATATNPISIKTTSQLATTASASSMAPVVTSPSLTGPLQSASGSAVATYSVPSISSTYQGAANIKVLGNFMWLLLALPVVF	480
YPS615/1-472	359	AGETTTGYTTKSIPTTYITTLIPGSNGAKNYETVATATNPISIKTTSQLATTASASSMAPVVTSPSLTGPLQSASGSAVATYSVPSISSTYQGAANIKVLGNFMWLLLALPVVV	472
YPS617/1-472	359	AGETTTGYTTKSIPTTYITTLIPGSNGAKNYETVATATNPISIKTTSQLATTASASSMAPVVTSPSLTGPLQSASGSAVATYSVPSISSTYQGAANIKVLGNFMWLLLALPVVV	472
YPS661/1-472	359	AGETTTGYTTKSIPTTYITTLIPGSNGAKNYETVATATNPISIKTTSQLATTASASSMAPVVTSPSLTGPLQSASGSAVATYSVPSISSTYQGAANIKVLGNFMWLLLALPVVV	472
YPS670/1-491	378	AGETTTGYTTKSIPTTYITTLIPGSNGAKNYETVATATNPISIKTTSQLATTASASSMAPVVTSPSLTGPLQSASGSAVATYSVPSISSTYQGAANIKVLGNFMWLLLALPVVF	491
YPS630/1-491	378	AGETTTGYTTKSIPTTYITTLIPGSNGAKNYETVATATNPISIKTTSQLATTASASSMAPVVTSPSLTGPLQSASGSAVATYSVPSISSTYQGAANIKVLGNFMWLLLALPVVF	491
UWOPS3-461.4/1-388	275	AGETTTGYTTKSIPTTYITTLIPGSNGAKNYETVATATNPISIKTTSQLATTASASSMAPVVTSPSLTGPLQSASGSAVATYSVPSISSTYQGAANIKVLGNFMWLLLALPVVV	388
Y55/1-491	378	AGETTTGYTTKSIPTTYITTLIPGSNGAKNYETVATATNPISIKTTSQLATTASASSMAPVVTSPSLTGPLQSASGSAVATYSVPSISSTYQGAANIKVLGNFMWLLLALPVVF	491
YS4/1-426	313	AGETTTGYTTKSIPTTYITTLIPGSNGAKNYETVATATNPISIKTTSQLATTASASSMAPVVTSPSLTGPLQSASGSAVATYSVPSISSTYQGAANIKVLGNFMWLLLALPVVF	426
DBVPG1788/1-511	398	AGETTTGYTTKSIPTTYITTLIPGSNGAKNYETVATATNPISIKTTSQLATTASASSMAPVVTSPSLTGPLQSASGSAVATYSVPSISSTYQGAANIKVLGNFMWLLLALPVVF	511
DBVPG6044/1-491	378	AGETTTGYTTKSIPTTYITTLIPGSNGAKNYETVATATNPISIKTTSQLATTASASSMAPVVTSPSLTGPLQSASGSAVATYSVPSISSTYQGAANIKVLGNFMWLLLALPVVF	491
Y9/1-486	373	AGETTTGYTTKSIPTTYITTLIPGSNGAKNYETVATATNPISIKTTSQLATTASASSMAPVVTSPSLTGPLQSASGSAVATYSVPSISSTYQGAANIKVLGNFMWLLLALPVVF	486

Conservation

Consensus

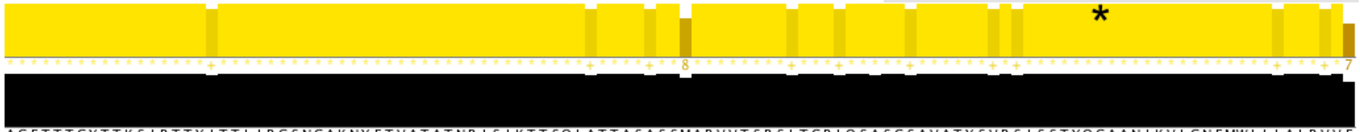

AGETTTGYTTKSIPTTYITTLIPGSNGAKNYETVATATNPISIKTTSQLATTASASSMAPVVTSPSLTGPLQSASGSAVATYSVPSISSTYQGAANIKVLGNFMWLLLALPVVF

Figure S4: Detection of recombination breakpoints within the FLO11 coding region. The data were analyzed with the A domain alone, the A-C domains concatenated, and data that included a part of the B domain that could be resolved and aligned. x-axis is the nucleotide position in the gene and the y-axis is support for a breakpoint, as determined via GARD (note the different scales).

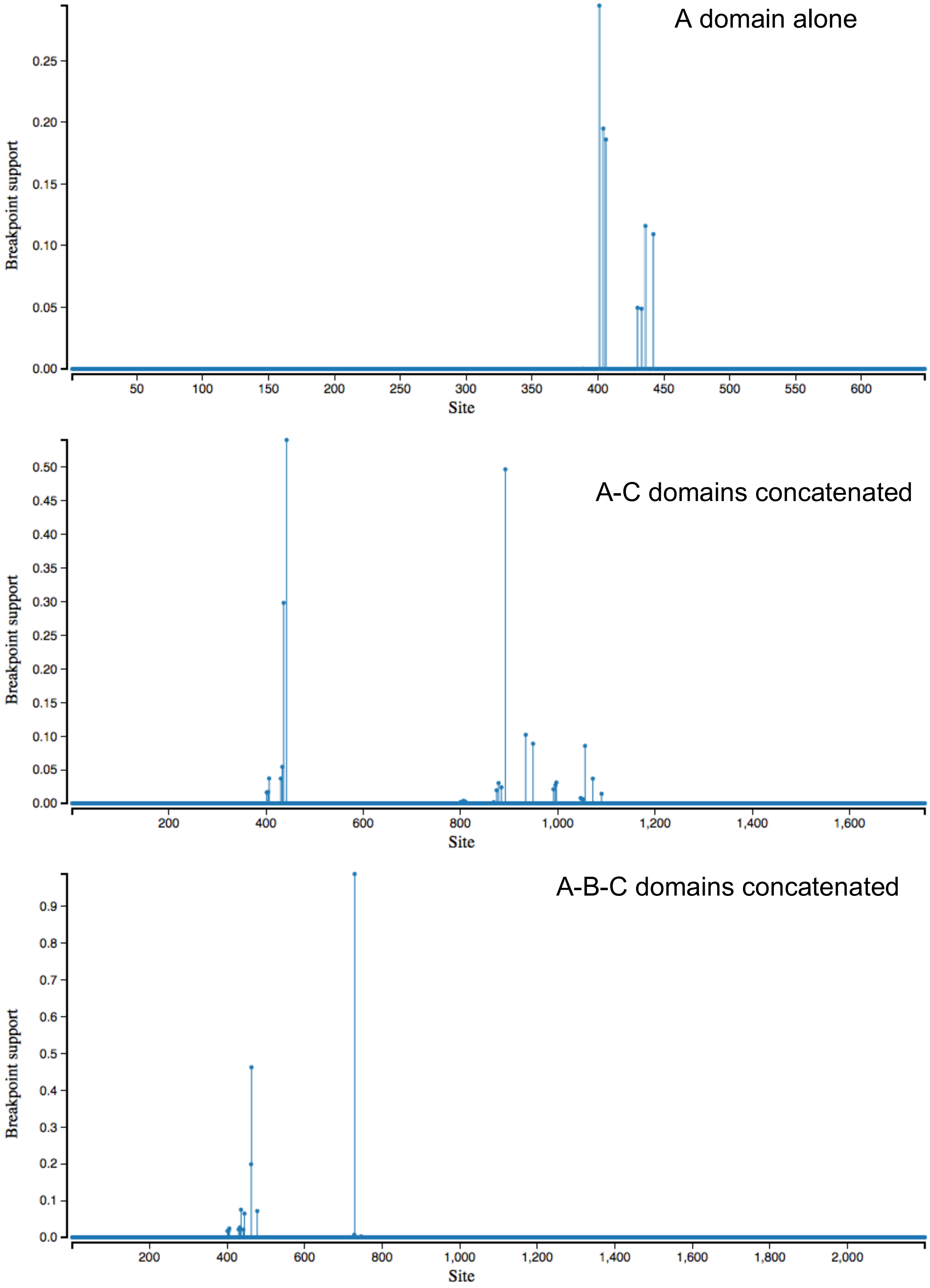

Figure S5: Genetic variation at *FLO11* compared to variation in genes that code for proteins with similar properties. Each data point represents a pairwise comparison between strains, for a given gene, as estimated in PAML using the yn00 function (N=54 strains and 43 genes for a total of ~65,000 data points). Red points represent *FLO11* comparisons.

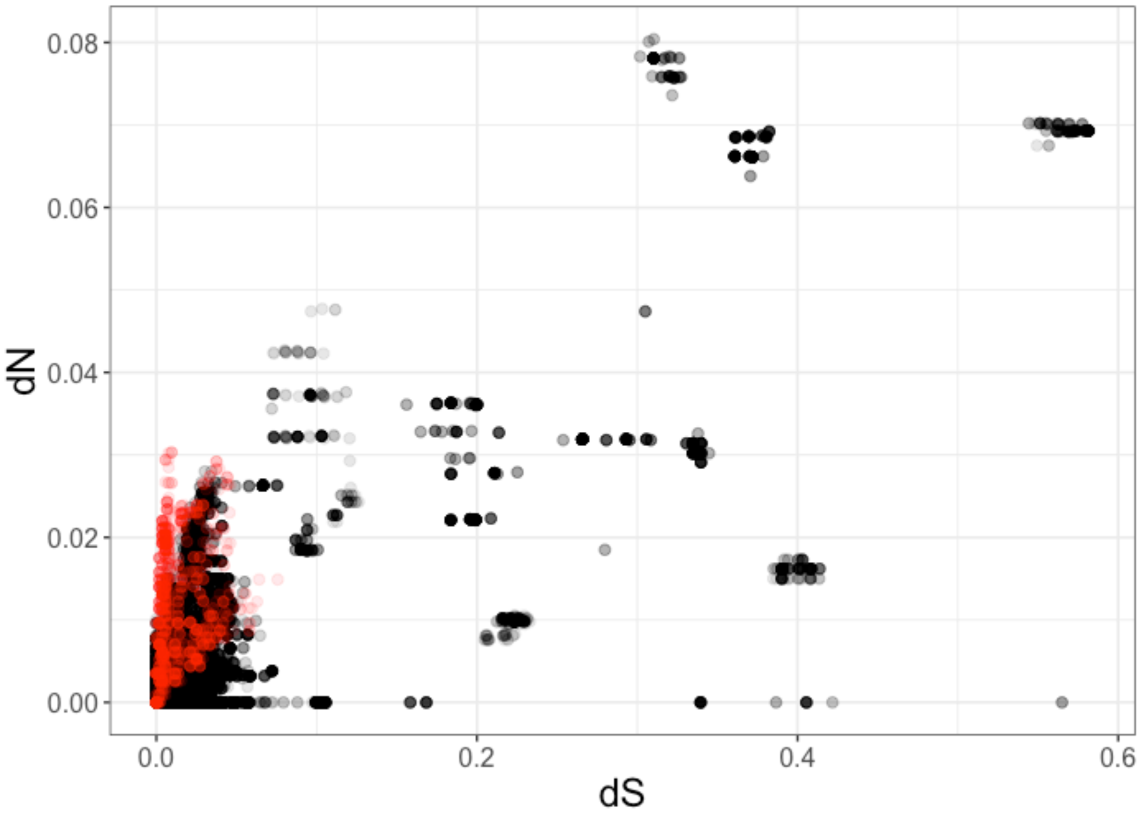

Figure S6: Upstream regulatory alignment. Position 1 is -3544; positions 3545-6 are the start codon.

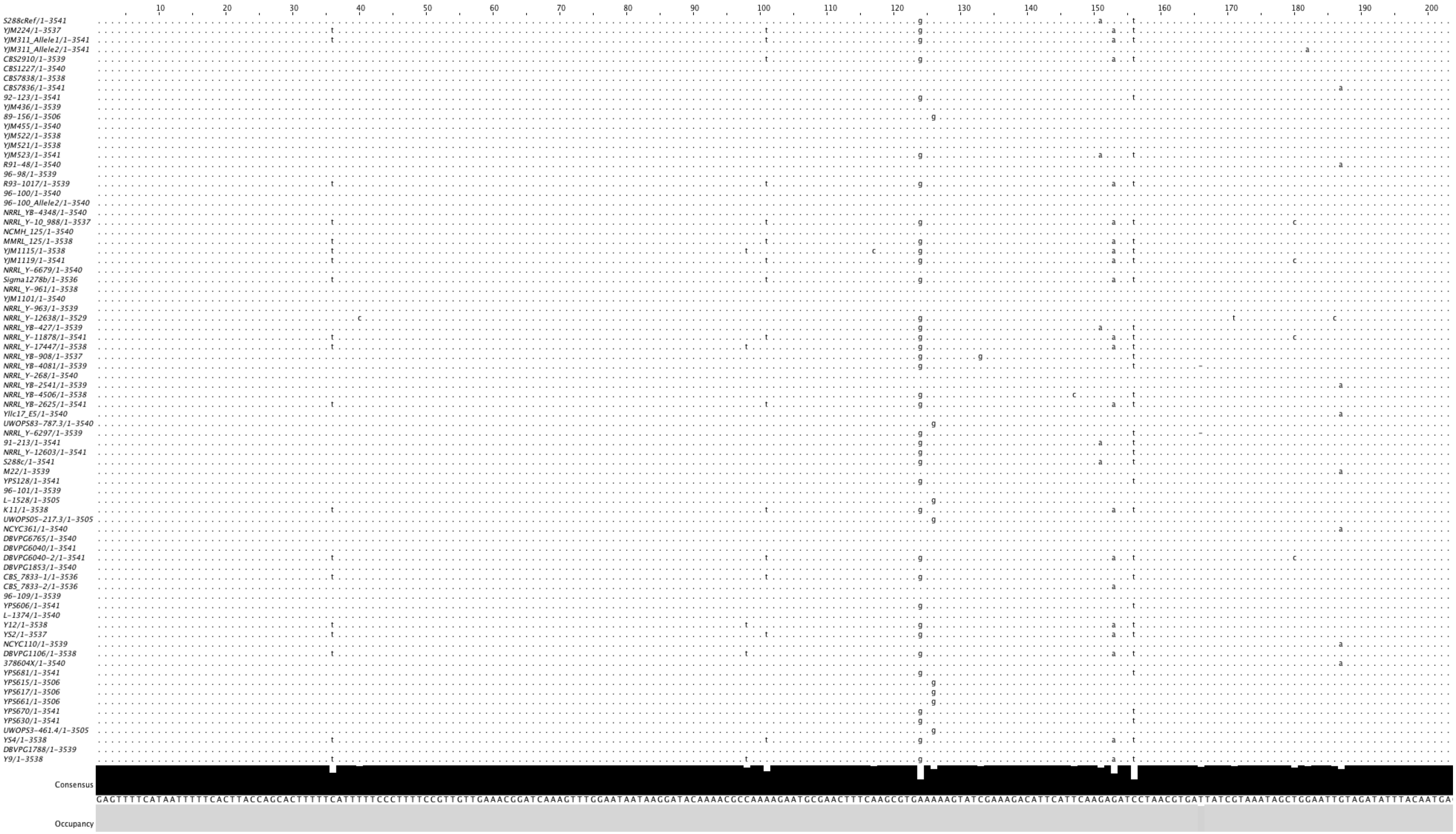

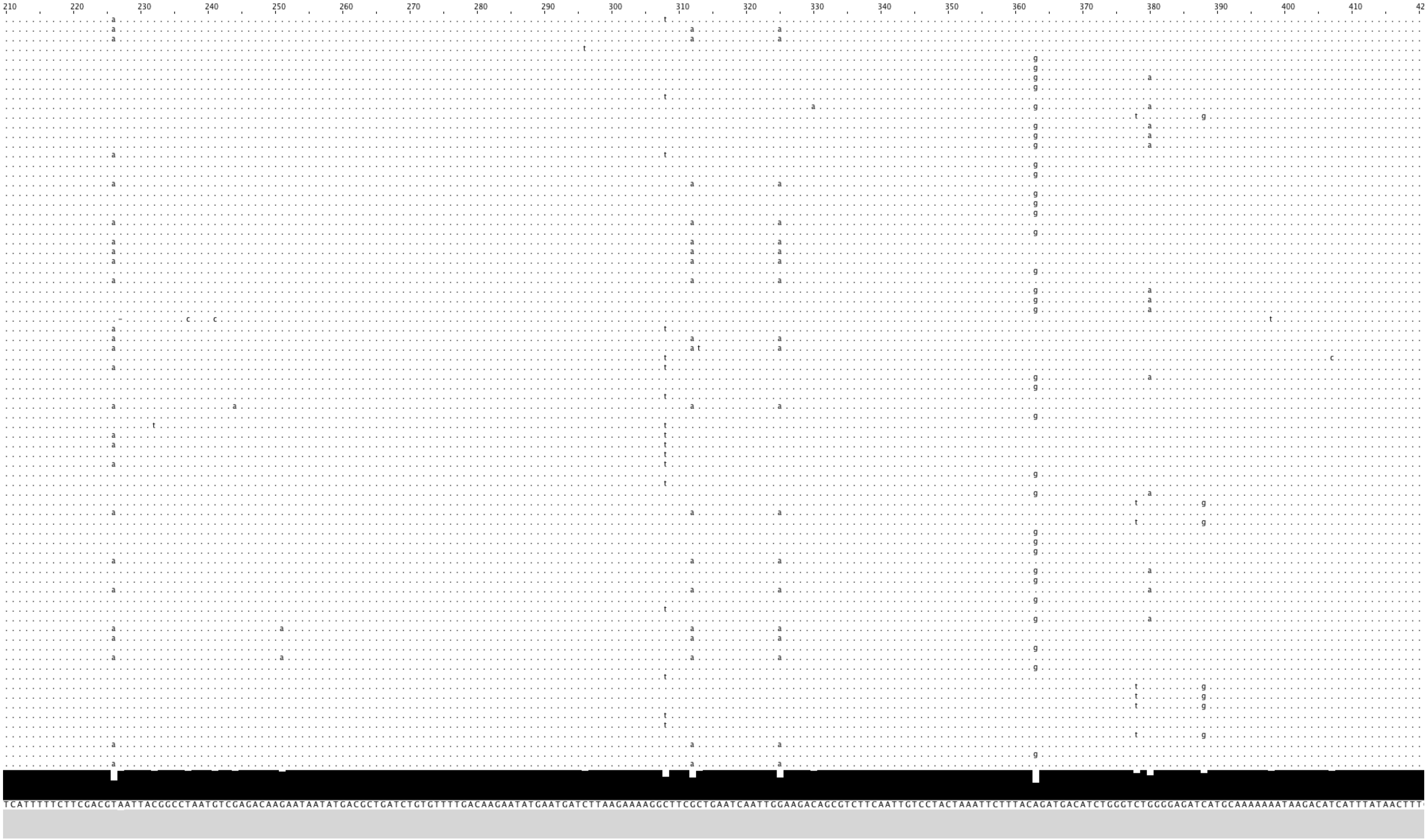
210 220 230 240 250 260 270 280 290 300 310 320 330 340 350 360 370 380 390 400 410 42
TCATTTTTCTTCGACGTAATTACGGCCTAATGTCGAGACAAGAATAATATGACGCTGATCTGTGTTTTGACAAGAATATGAATGATCTTAAGAAAAGGCTTCGCTGAATCAATTGGAAGACAGCGTCTTCAATTGTCCTACTAAATTCTTTACAGATGACATCTGGGTCTGGGGAGATCATGCAAAAAAATAAGACATCATTTATAACTTT

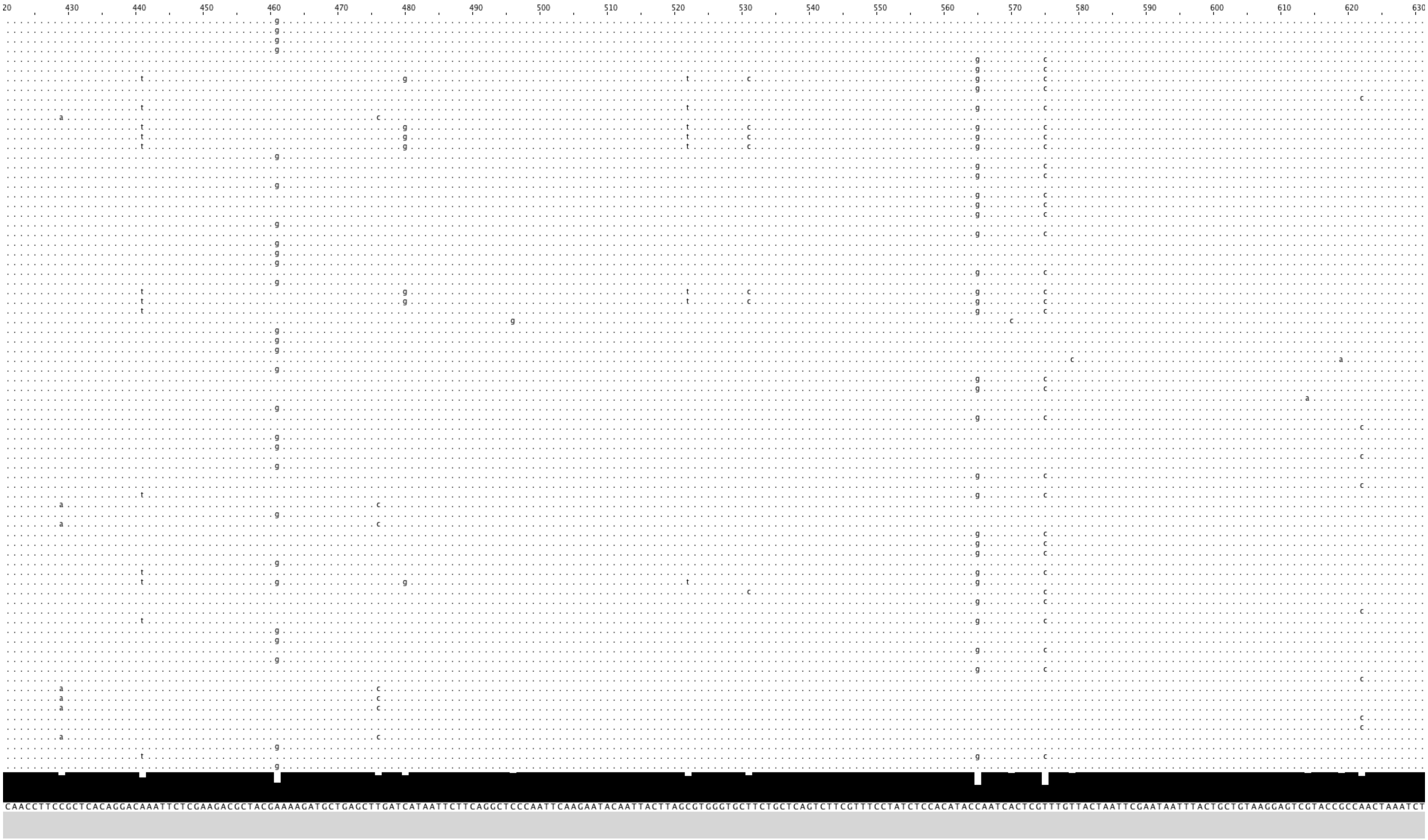
20 430 440 450 460 470 480 490 500 510 520 530 540 550 560 570 580 590 600 610 620 630
CAACCTTCCGCTCACAGGACAAATTCTCGAAGACGCTACGAAAAGATGCTGAGCTTGATCATAATTCTTCAGGCTCCCAATTCAAGAATACAATTACTTAGCGTGGGTGCTTCTGCTCAGTCTTCGTTTCCTATCTCCACATACCAATCACTCGTTTGTTACTAATTCGAATAATTTACTGCTGTAAGGAGTCGTACCGCCAACTAAATCT

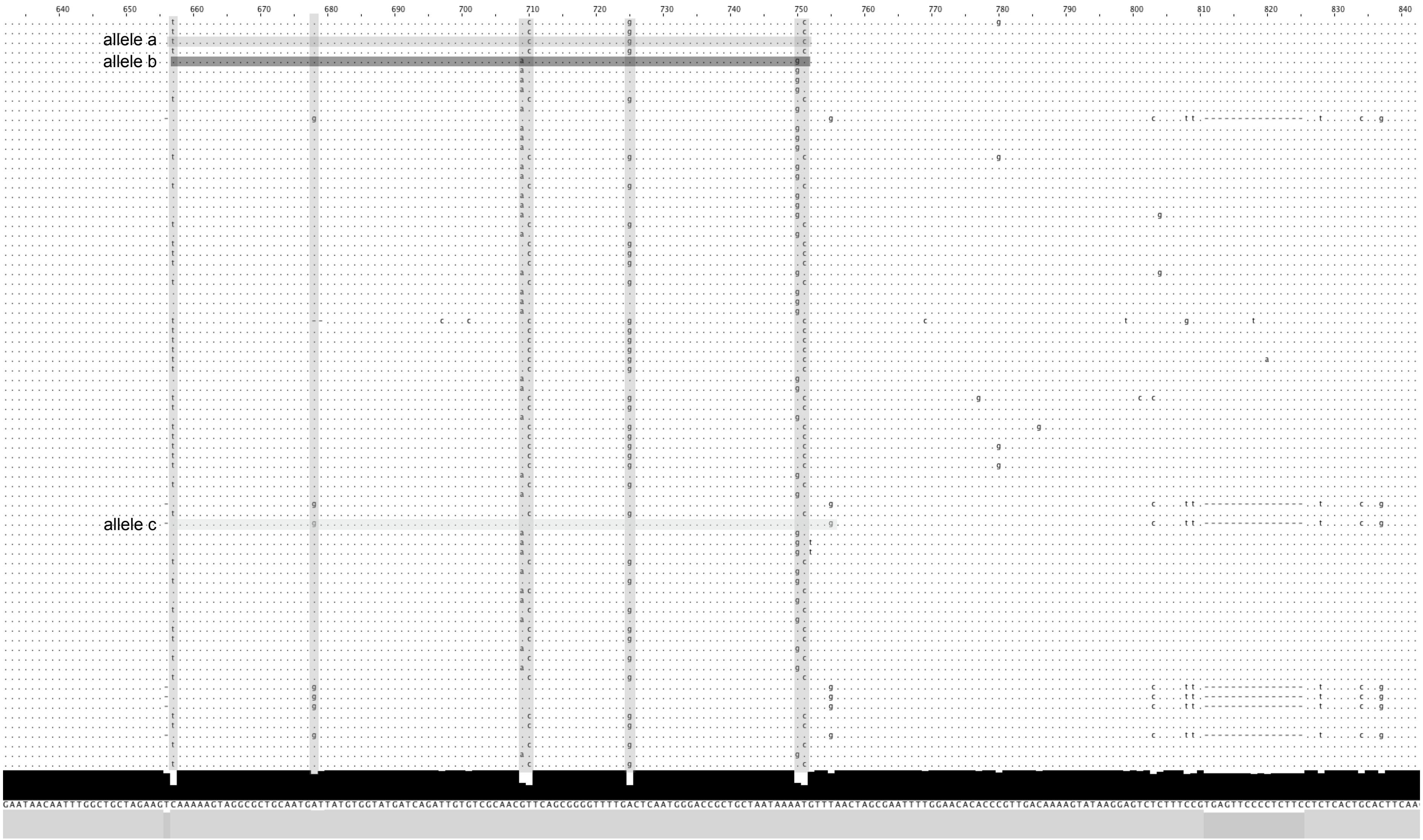
allele a
allele b
allele c

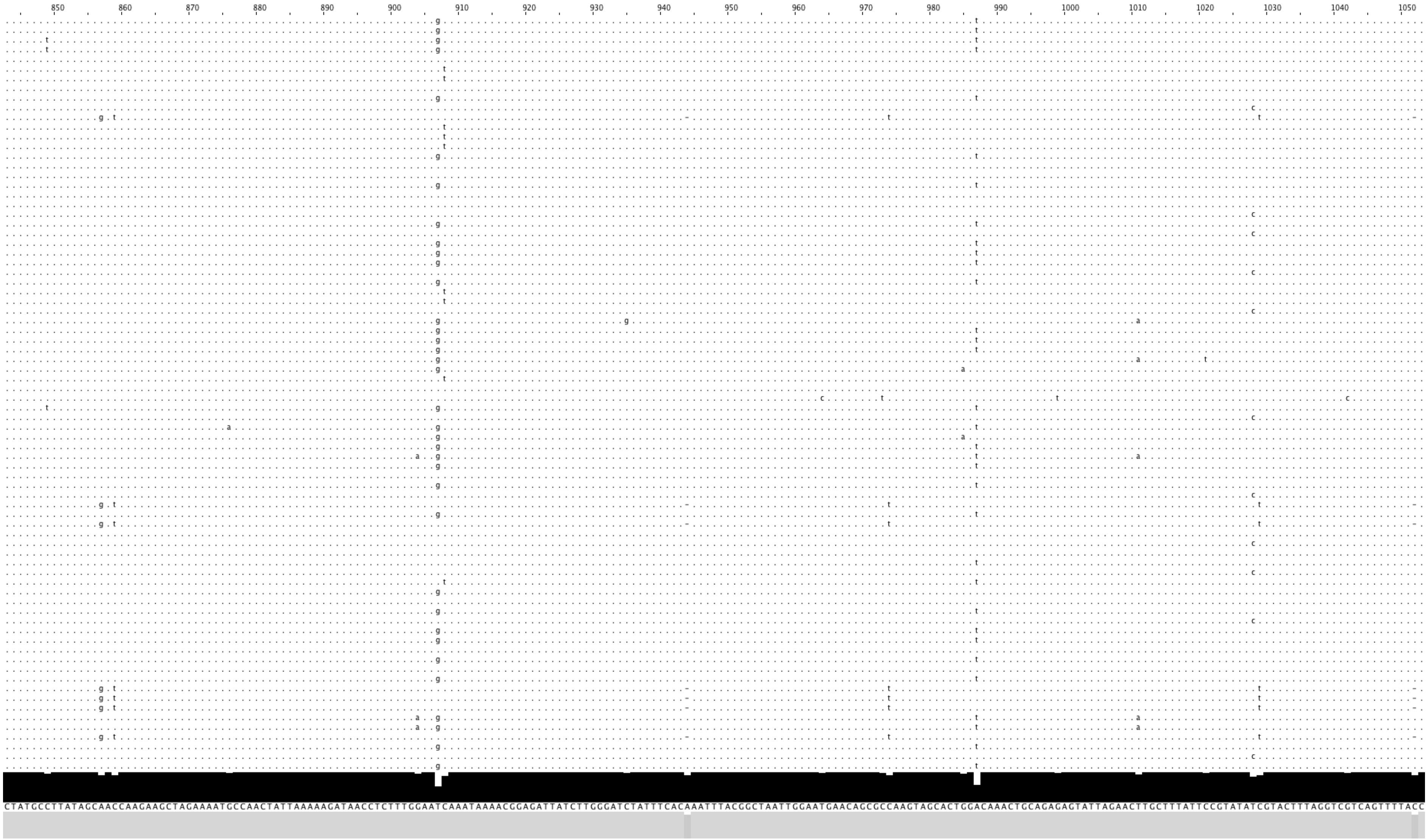

850
860
870
880
890
900
910
920
930
940
950
960
970
980
990
1000
1010
1020
1030
1040
1050

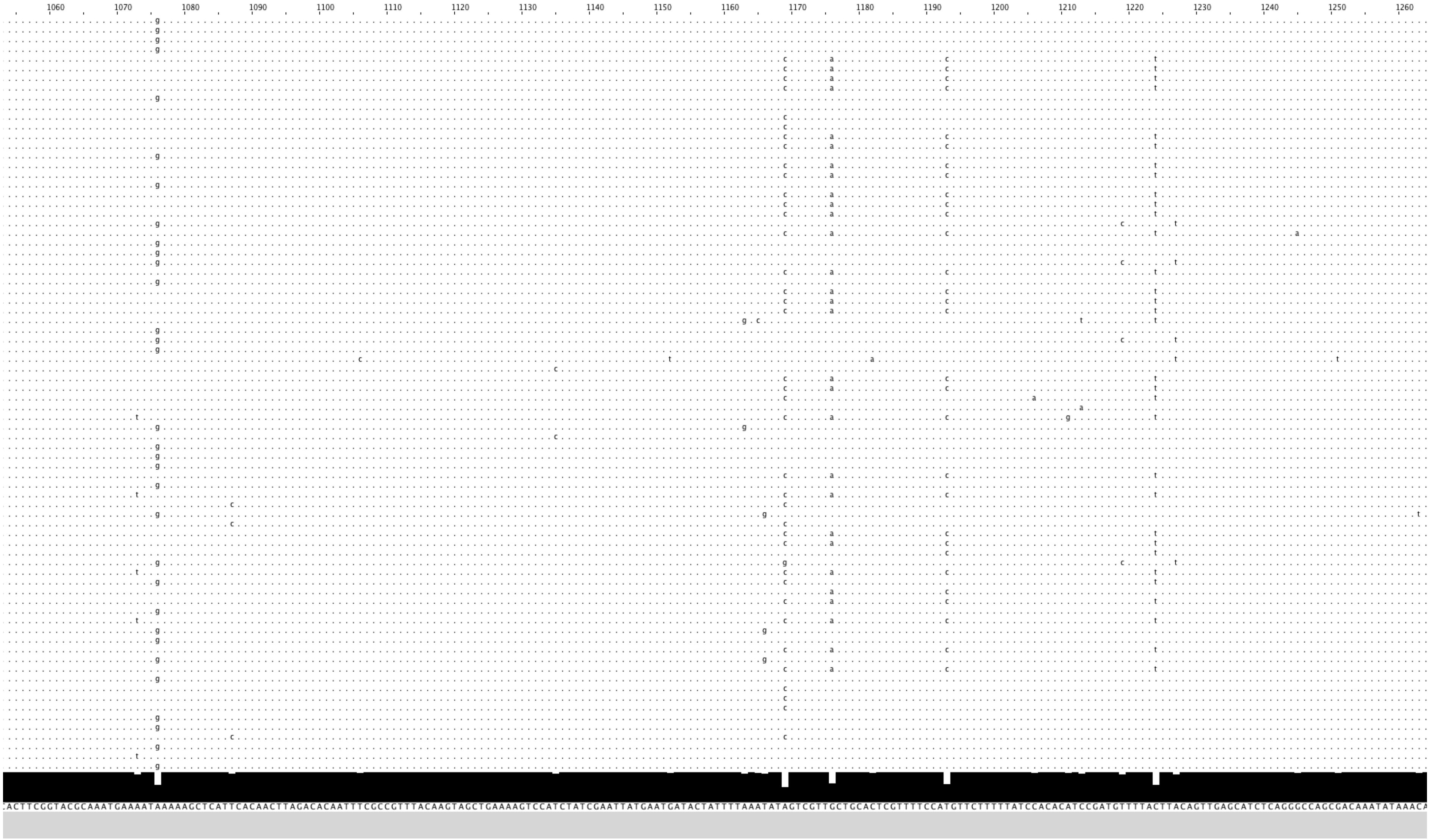

1060 1070 1080 1090 1100 1110 1120 1130 1140 1150 1160 1170 1180 1190 1200 1210 1220 1230 1240 1250 1260
CACTTCGGTACGCAAATGAAAATAAAAAGCTCATTCACAACTTAGACACAATTTCGCCGTTTACAAGTAGCTGAAAAGTCCATCTATCGAATTATGAATGATACTATTTTAAATATAGTCGTTGCTGCACTCGTTTTCCATGTTCTTTTTATCCACACATCCGATGTTTTACTTACAGTTGAGCATCTCAGGGCCAGCGACAAATATAAACA

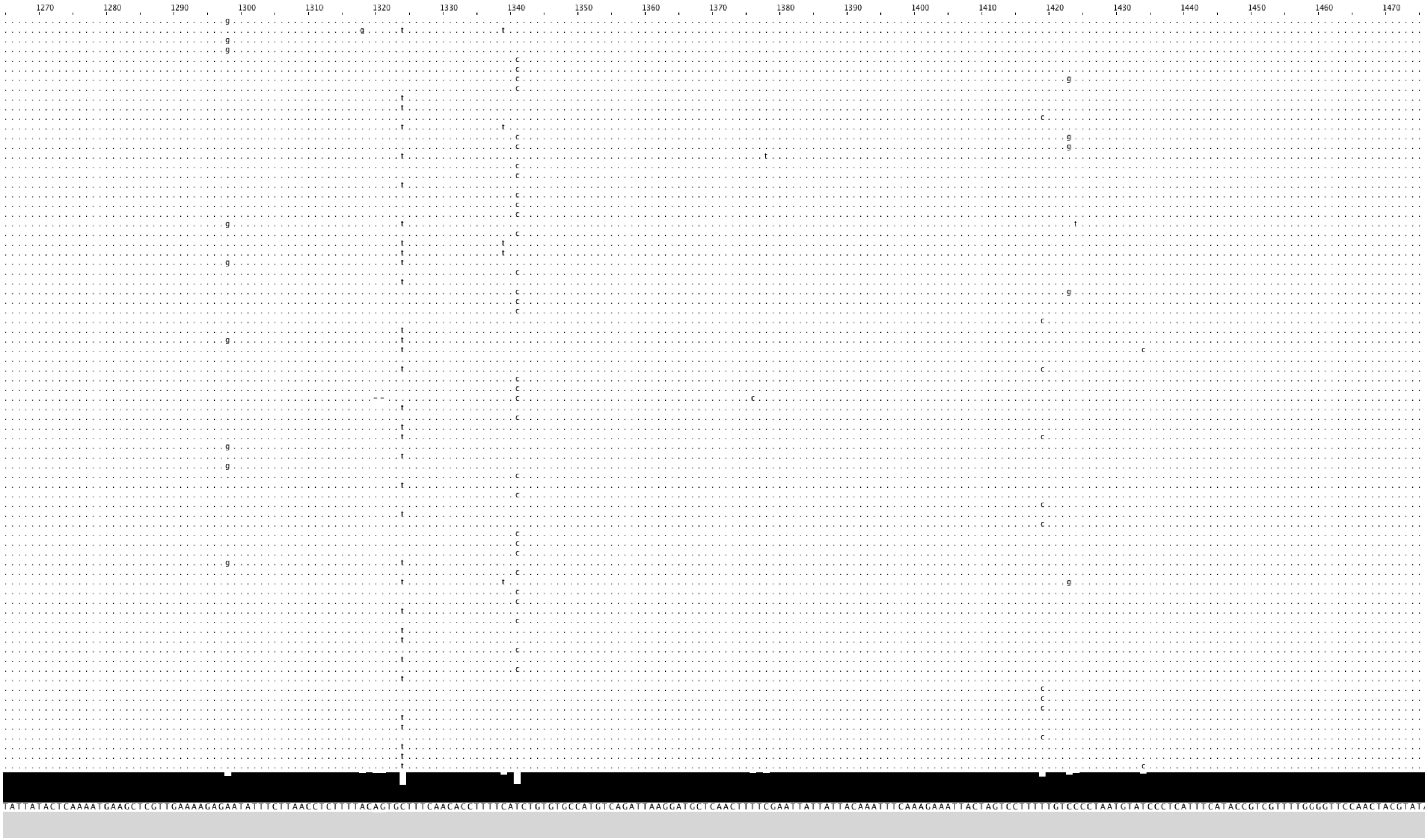
1270 1280 1290 1300 1310 1320 1330 1340 1350 1360 1370 1380 1390 1400 1410 1420 1430 1440 1450 1460 1470
TATTATACTCAAAATGAAGCTCGTTGAAAAGAGAATATTTCTTAACCTCTTTTACAGTGCTTTCAACACCTTTTCATCTGTGTGCCATGTCAGATTAAGGATGCTCAACTTTTCGAATTATTATTACAAATTTCAAAGAAATTACTAGTCCTTTTTGTCCCCTAATGTATCCCTCATTTCATACCGTCGTTTTGGGGTTCCAACTACGTAT

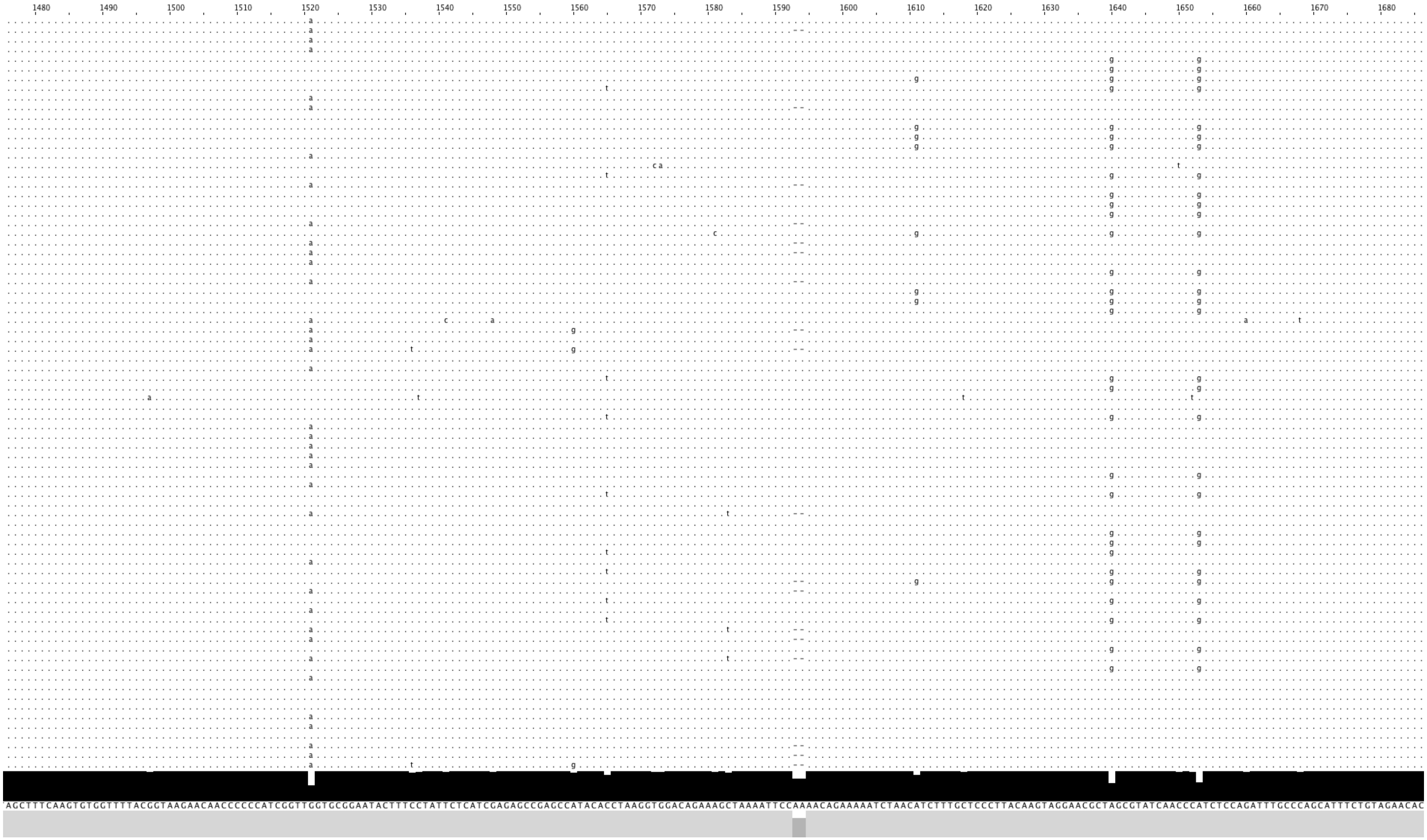
1480 1490 1500 1510 1520 1530 1540 1550 1560 1570 1580 1590 1600 1610 1620 1630 1640 1650 1660 1670 1680
AGCTTTCAAGTGTGGTTTTACGGTAAGAACAACCCCCCATCGGTTGGTGCGGAATACTTTCCTATTCTCATCGAGAGCCGAGCCATACACCTAAGGTGGACAGAAAGCTAAAATTCCAAAACAGAAAAATCTAACATCTTTGCTCCCTTACAAGTAGGAACGCTAGCGTATCAACCCATCTCCAGATTTGCCCAGCATTTCTGTAGAACAC

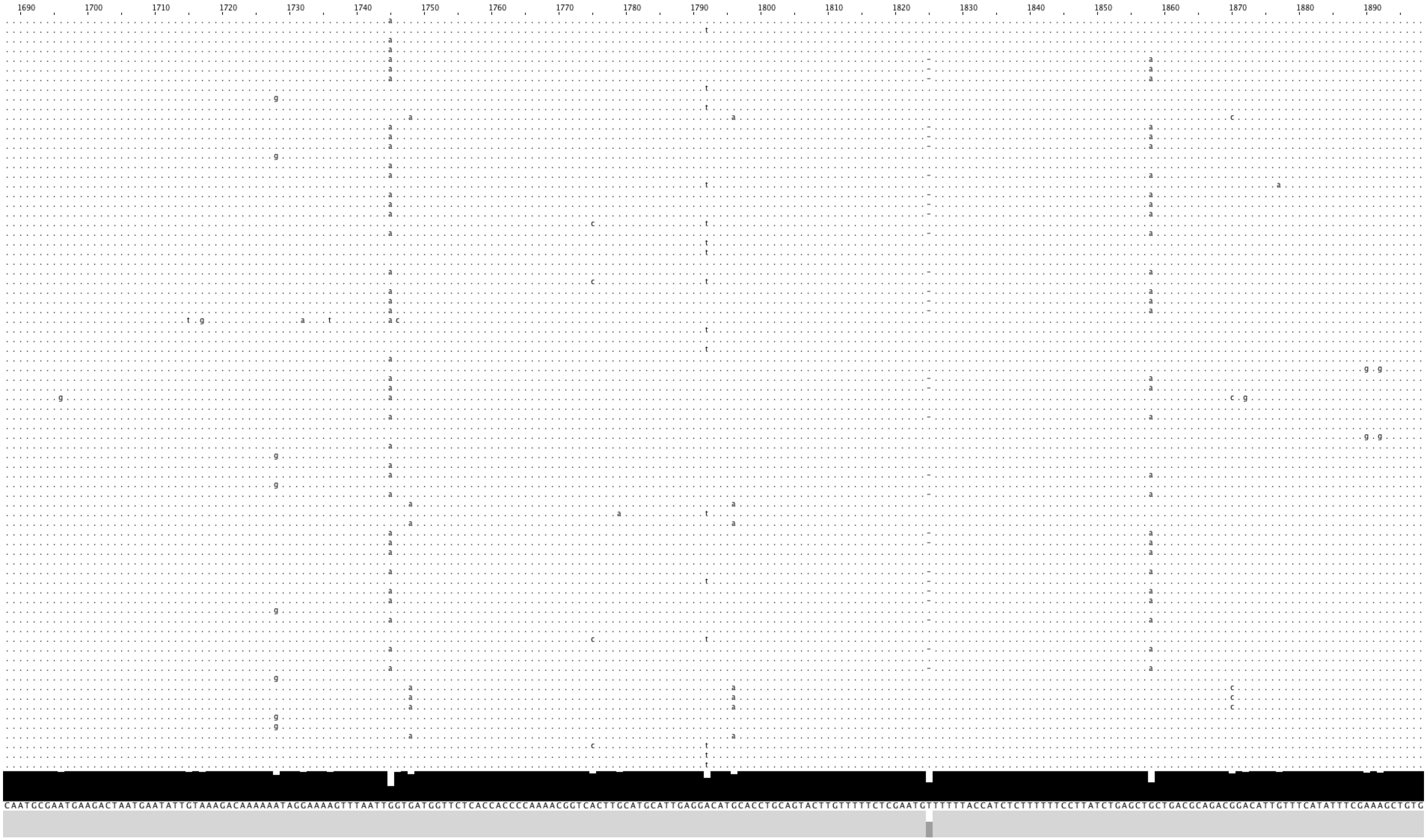
1690 1700 1710 1720 1730 1740 1750 1760 1770 1780 1790 1800 1810 1820 1830 1840 1850 1860 1870 1880 1890
CAATGCGAATGAAGACTAATGAATATTGTAAAGACAAAAAATAGGAAAAGTTTAATTGGTGATGGTTCTCACCACCCCAAAACGGTCACTTGCATGCATTGAGGACATGCACCTGCAGTACTTGTTTTTCTCGAATGTTTTTTACCATCTCTTTTTTCCTTATCTGAGCTGCTGACGCAGACGGACATTGTTTCATATTTCGAAAGCTGTG

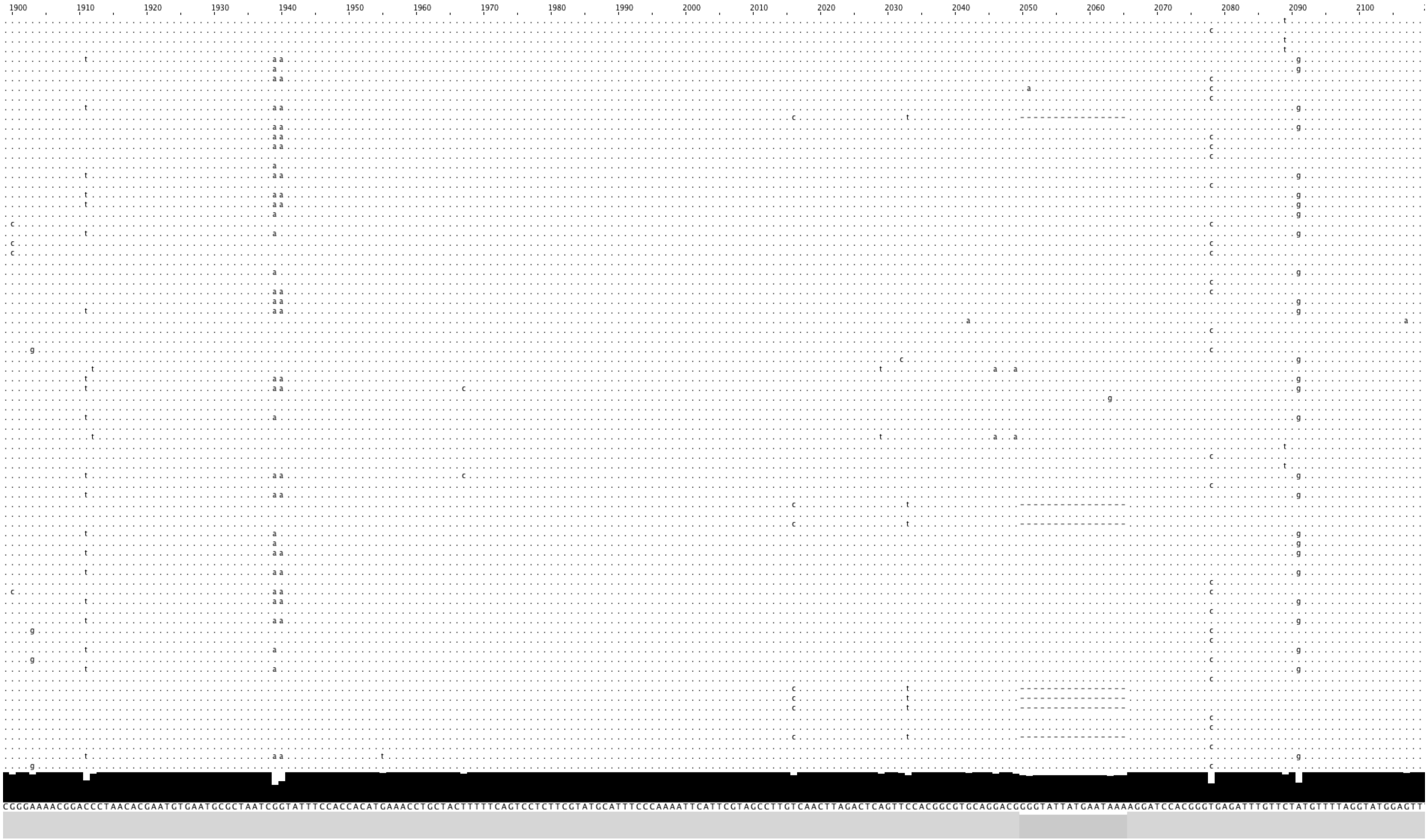

1900 1910 1920 1930 1940 1950 1960 1970 1980 1990 2000 2010 2020 2030 2040 2050 2060 2070 2080 2090 2100
CGGGAAAACGGACCCTAACACGAATGTGAATGCGCTAATCGGTATTTCCACCACATGAAACCTGCTACTTTTTCAGTCCTCTTCGTATGCATTTCCCAAAATTCATTCGTAGCCTTGTCAACTTAGACTCAGTTCCACGGCGTGCAGGACGGGGTATTATGAATAAAAGGATCCACGGGTGAGATTTGTTCTATGTTTTAGGTATGGAGTT

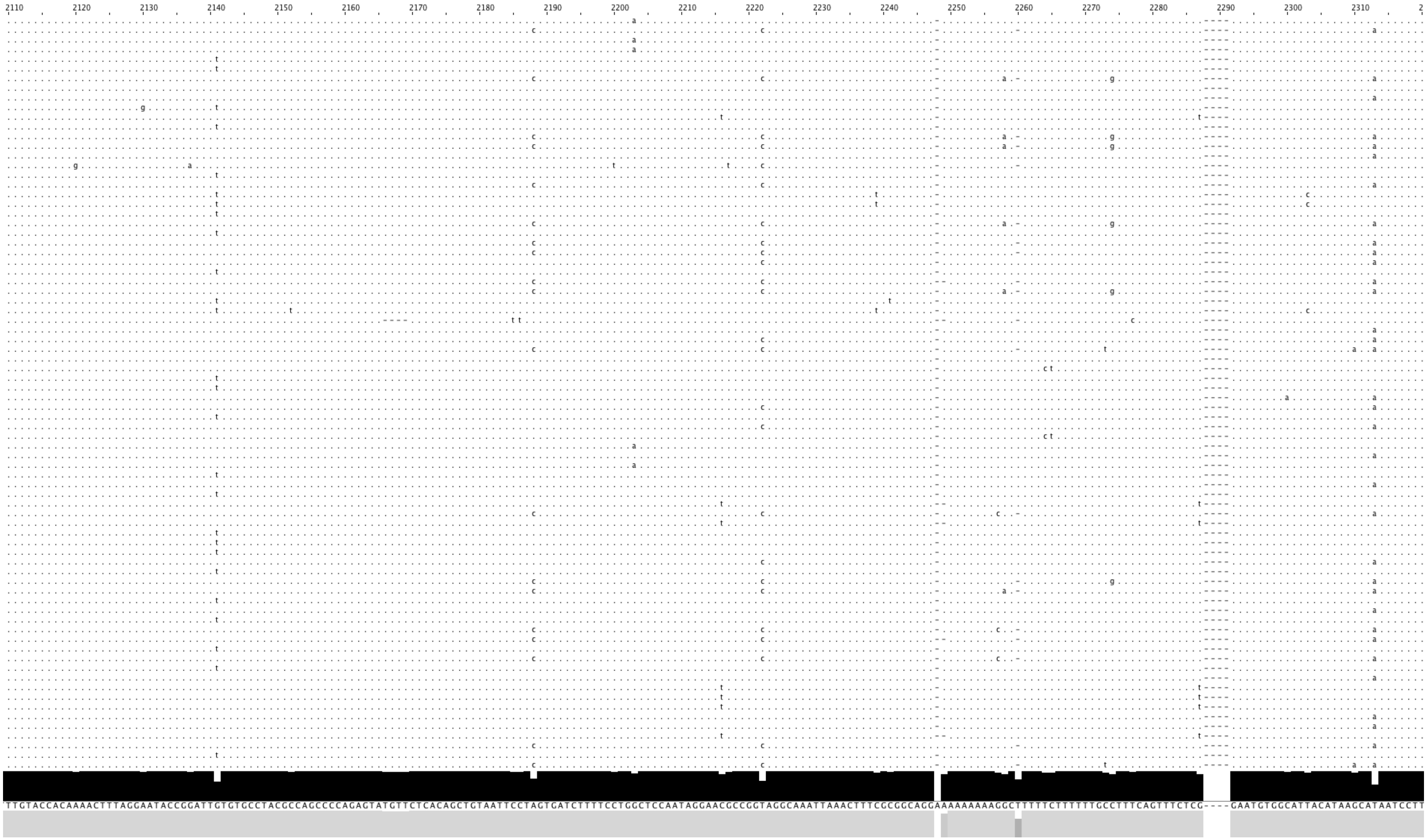

2110 2120 2130 2140 2150 2160 2170 2180 2190 2200 2210 2220 2230 2240 2250 2260 2270 2280 2290 2300 2310
TTGTACCACAAAACTTTAGGAATACCGGATTGTGTGCCTACGCCAGCCCCAGAGTATGTTCTCACAGCTGTAATTCCTAGTGATCTTTTCCTGGCTCCAATAGGAACGCCGGTAGGCAAATTAAACTTTCGCGGCAGGAAAAAAAAAGGCTTTTTCTTTTTTGCCTTTCAGTTTCTCG----GAATGTGGCATTACATAAGCATAATCCTT

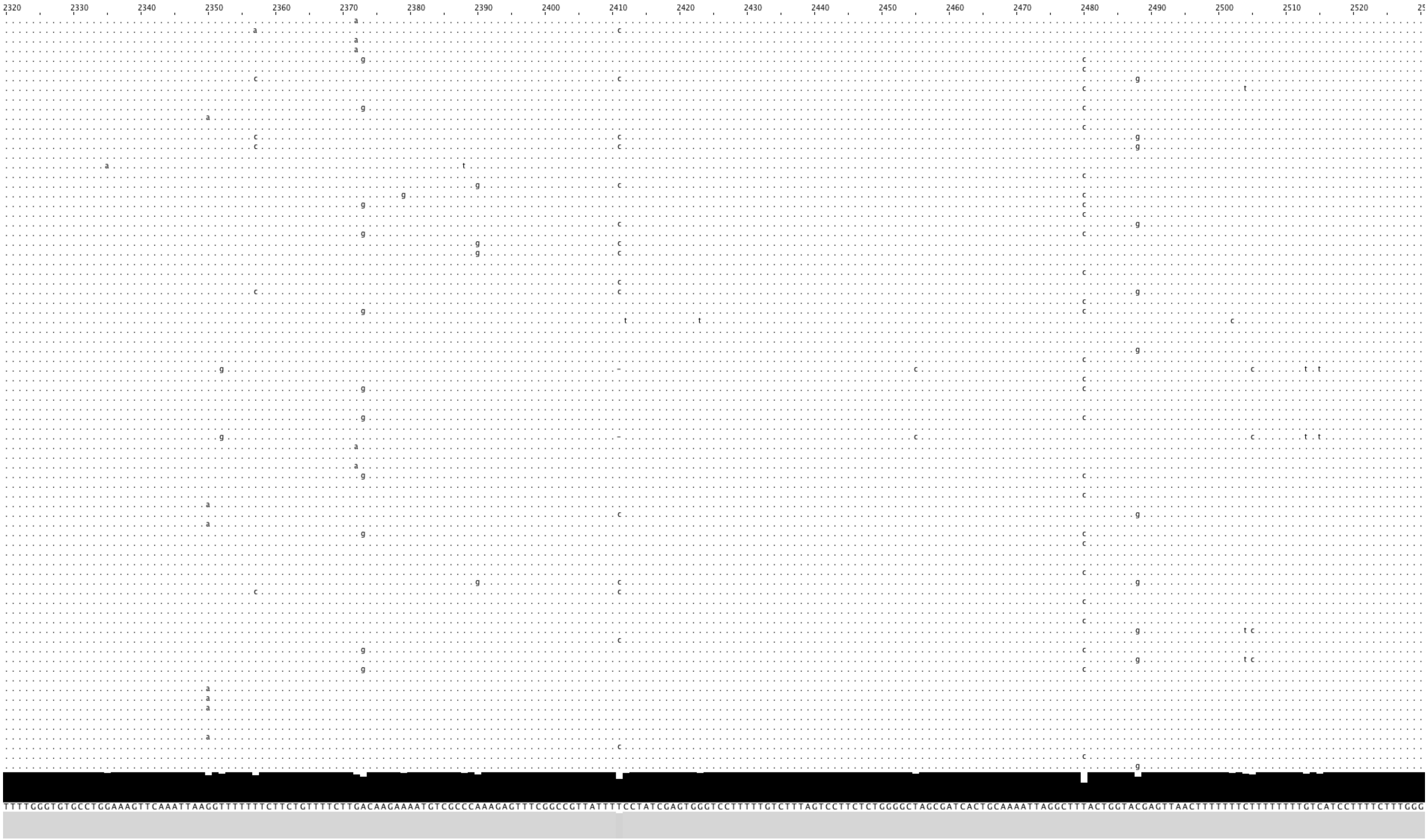

2320 2330 2340 2350 2360 2370 2380 2390 2400 2410 2420 2430 2440 2450 2460 2470 2480 2490 2500 2510 2520
TTTTGGGTGTGCCTGGAAAGTTCAAATTAAGGTTTTTTTCTTCTGTTTTCTTGACAAGAAAATGTCGCCCAAAGAGTTTCGGCCGTTATTTTCCTATCGAGTGGGTCCTTTTTGTCTTTAGTCCTTCTCTGGGGCTAGCGATCACTGCAAAATTAGGCTTTACTGGTACGAGTTAACTTTTTTTCTTTTTTTTGTCATCCTTTTCTTTGGG

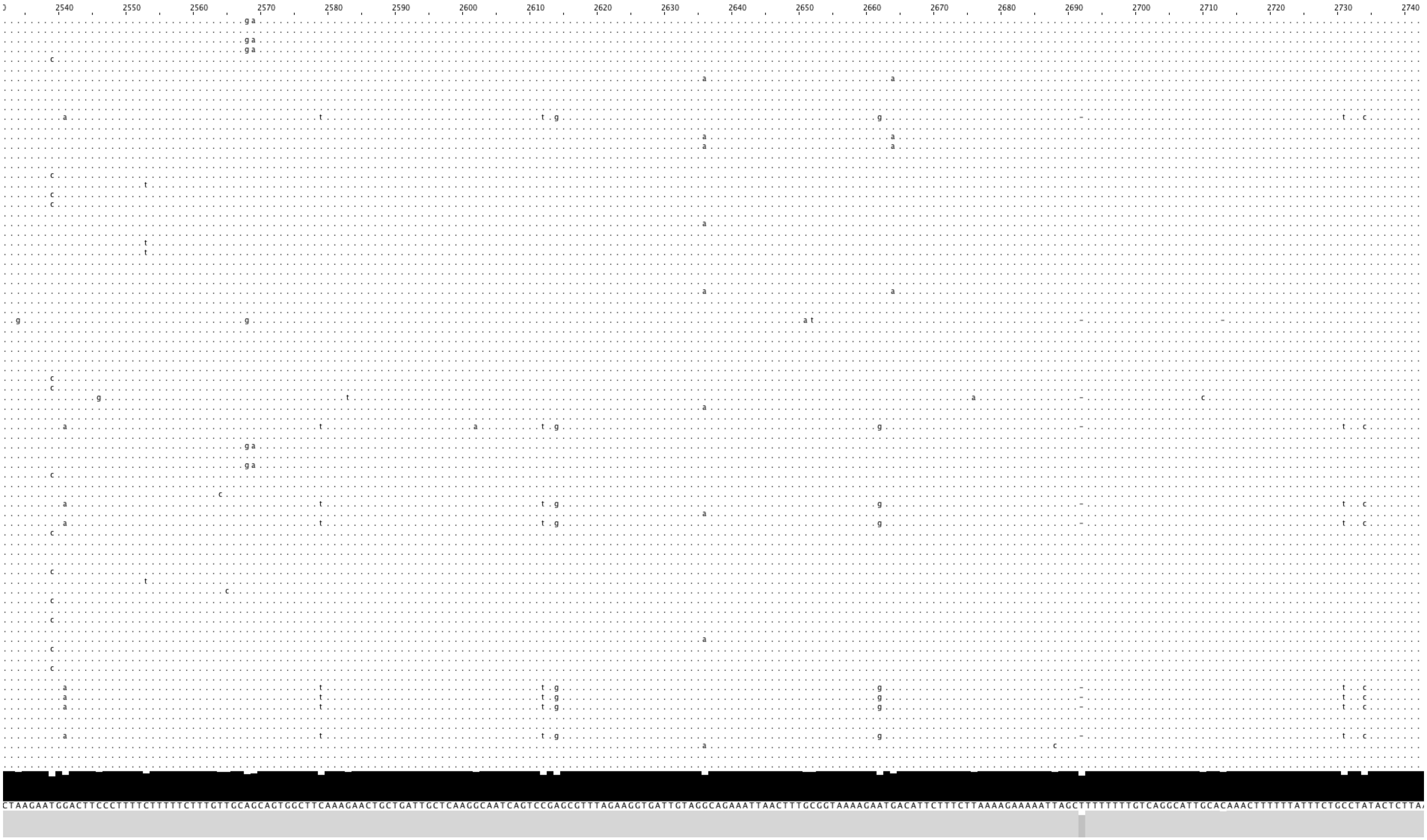

2540 2550 2560 2570 2580 2590 2600 2610 2620 2630 2640 2650 2660 2670 2680 2690 2700 2710 2720 2730 2740
CTAAGAATGGACTTCCCTTTTCTTTTTCTTTGTTGCAGCAGTGGCTTCAAAGAACTGCTGATTGCTCAAGGCAATCAGTCCGAGCGTTTAGAAGGTGATTGTAGGCAGAAATTAACTTTGCGGTAAAAGAATGACATTCTTTCTTAAAAGAAAAATTAGCTTTTTTTTGTCAGGCATTGCACAAACTTTTTTATTTCTGCCTATACTCTTA

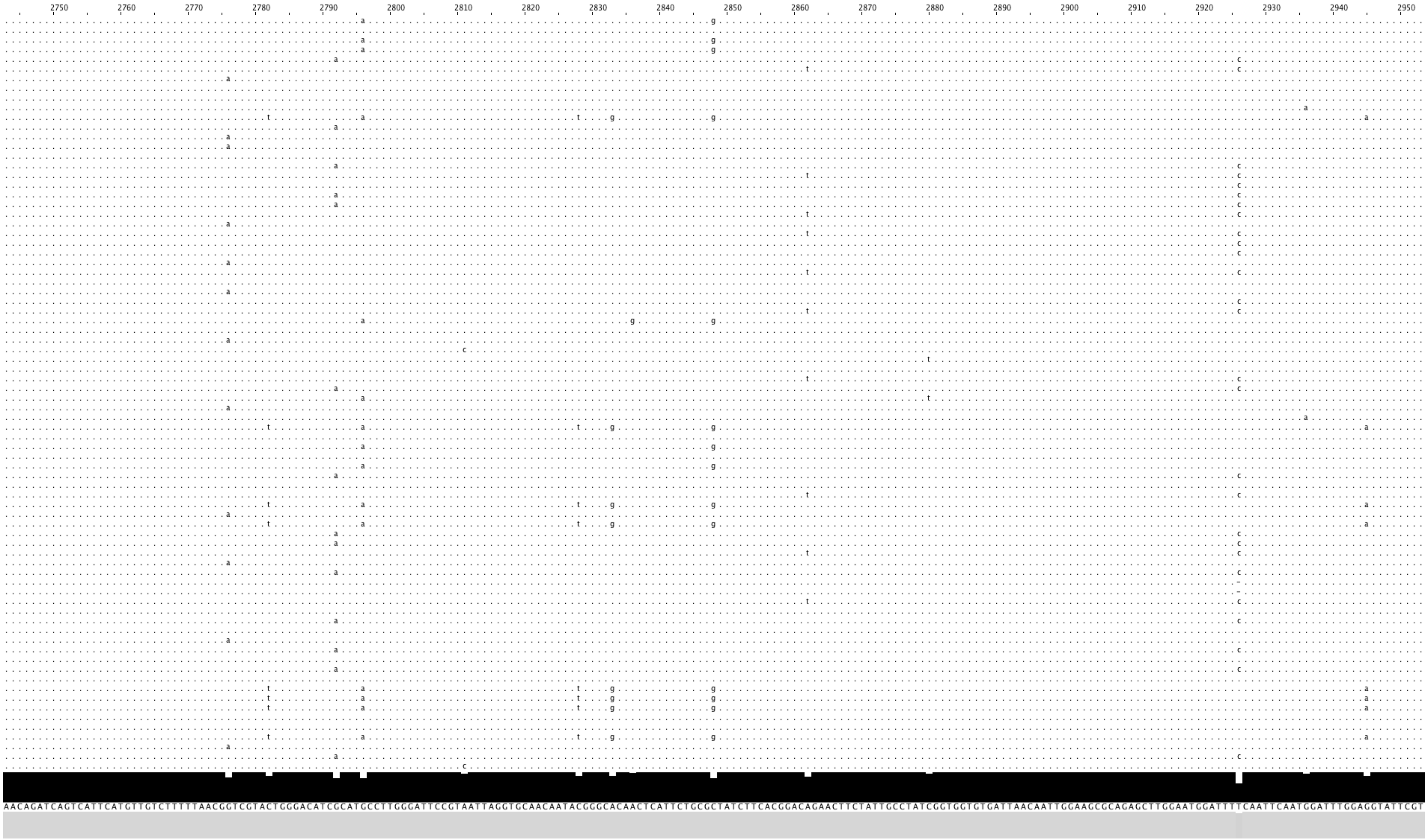
2750 2760 2770 2780 2790 2800 2810 2820 2830 2840 2850 2860 2870 2880 2890 2900 2910 2920 2930 2940 2950
AACAGATCAGTCATTCATGTTGTCTTTTTAACGGTCGTACTGGGACATCGCATGCCTTGGGATTCCGTAATTAGGTGCAACAATACGGGCACAACTCATTCTGCGCTATCTTCACGGACAGAACTTCTATTGCCTATCGGTGGTGTGATTAACAATTGGAAGCGCAGAGCTTGGAATGGATTTTCAATTCAATGGATTTGGAGGTATTCGT

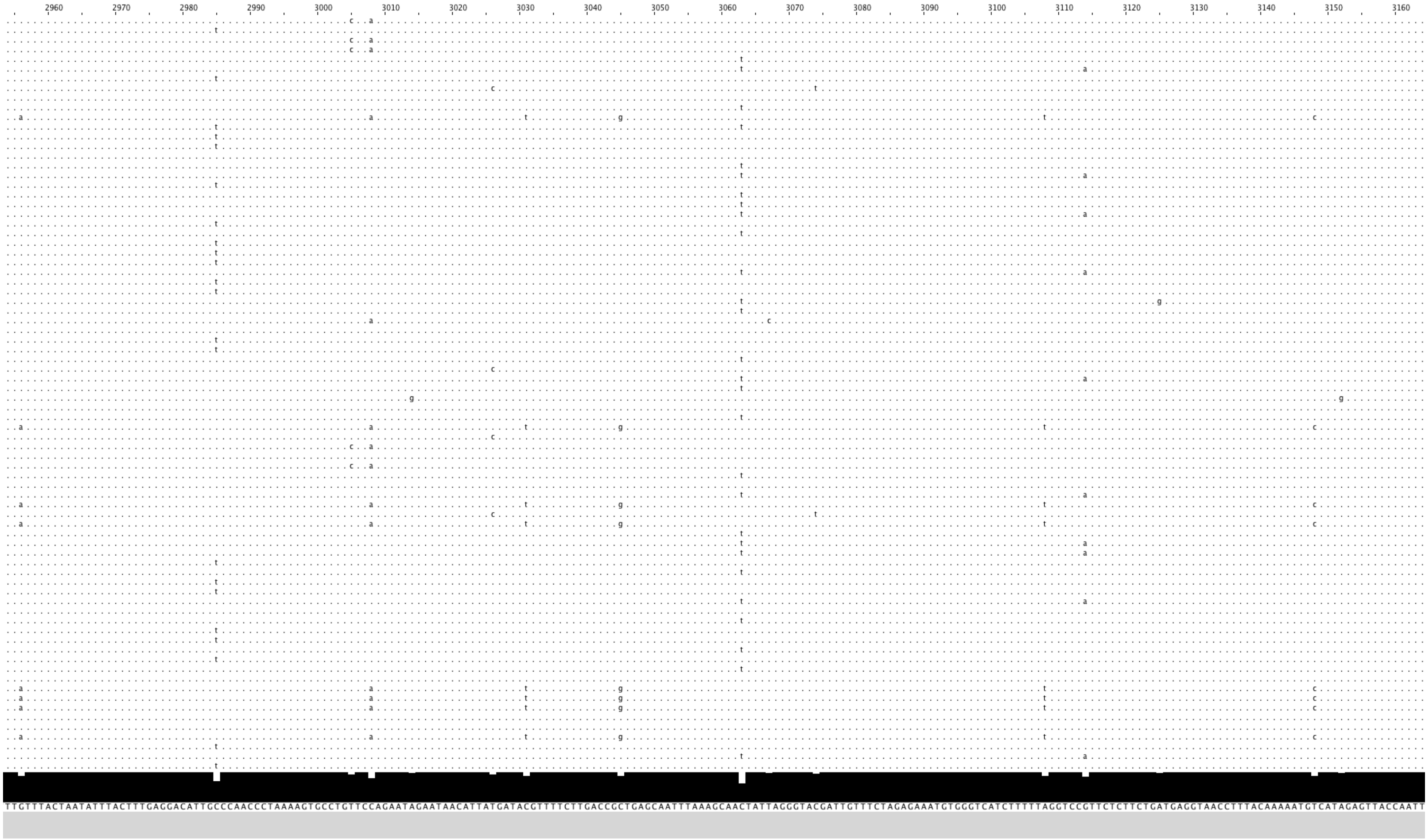
2960
2970
2980
2990
3000
3010
3020
3030
3040
3050
3060
3070
3080
3090
3100
3110
3120
3130
3140
3150
3160
TTGTTTACTAATATTTACTTTGAGGACATTGCCCAACCCTAAAAGTGCCTGTTCCAGAATAGAATAACATTATGATACGTTTTCTTGACCGCTGAGCAATTTAAAGCAACTATTAGGGTACGATTGTTTCTAGAGAAATGTGGGTCATCTTTTTAGGTCCGTTCTCTTCTGATGAGGTAACCTTTACAAAAATGTCATAGAGTTACCAATT

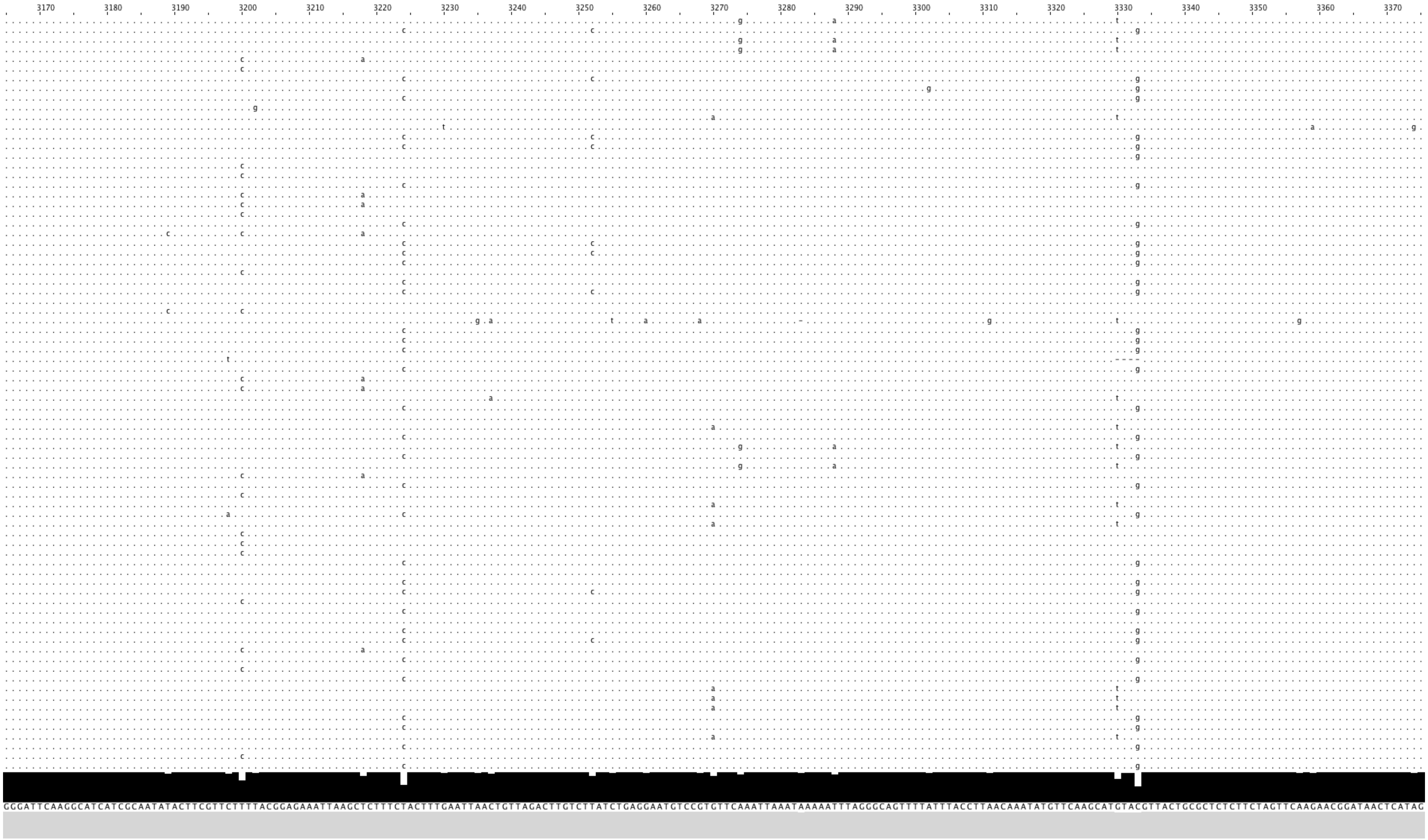
3170 3180 3190 3200 3210 3220 3230 3240 3250 3260 3270 3280 3290 3300 3310 3320 3330 3340 3350 3360 3370
GGGATTCAAGGCATCATCGCAATATACTTCGTTCTTTTACGGAGAAATTAAGCTCTTTCTACTTTGAATTAACTGTTAGACTTGTCTTATCTGAGGAATGTCCGTGTTCAAATTAAATAAAAATTTAGGGCAGTTTTATTTACCTTAACAAATATGTTCAAGCATGTACGTTACTGCGCTCTCTTCTAGTTCAAGAACGGATAACTCATAG

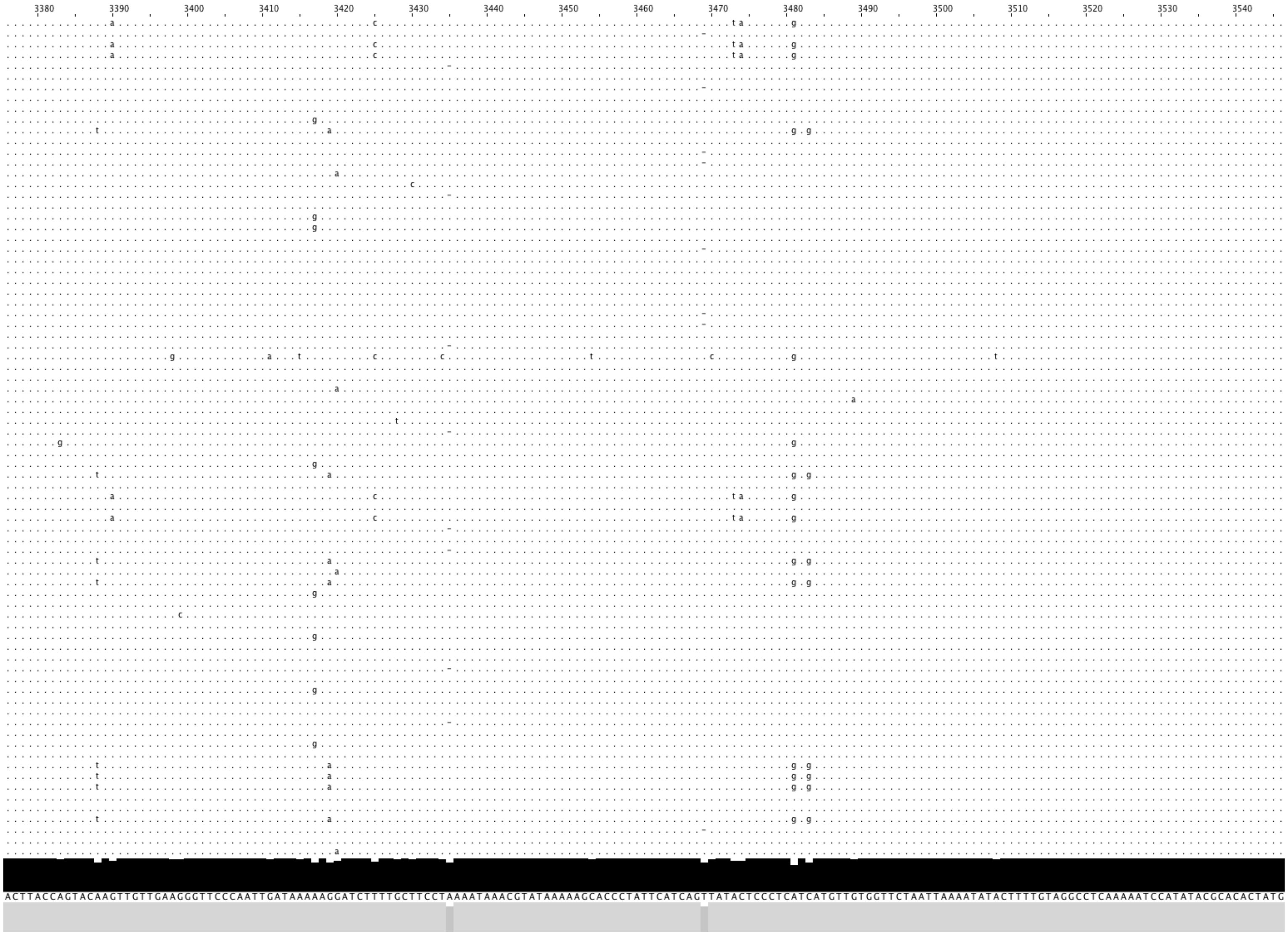
3380 3390 3400 3410 3420 3430 3440 3450 3460 3470 3480 3490 3500 3510 3520 3530 3540
ACTTACCAGTACAAGTTGTTGAAGGGTTCCCAATTGATAAAAAGGATCTTTTGCTTCCTAAAATAAACGTATAAAAAGCACCCTATTCATCAGTTATACTCCCTCATCATGTTGTGGTTCTAATTAAAATATACTTTTGTAGGCCTCAAAAATCCATATACGCACACTATG

Figure S7: Distribution of Tajima's D values in the upstream and downstream regions. Red points represent the region implicated in binding to the histone deacetylase RpdL3.

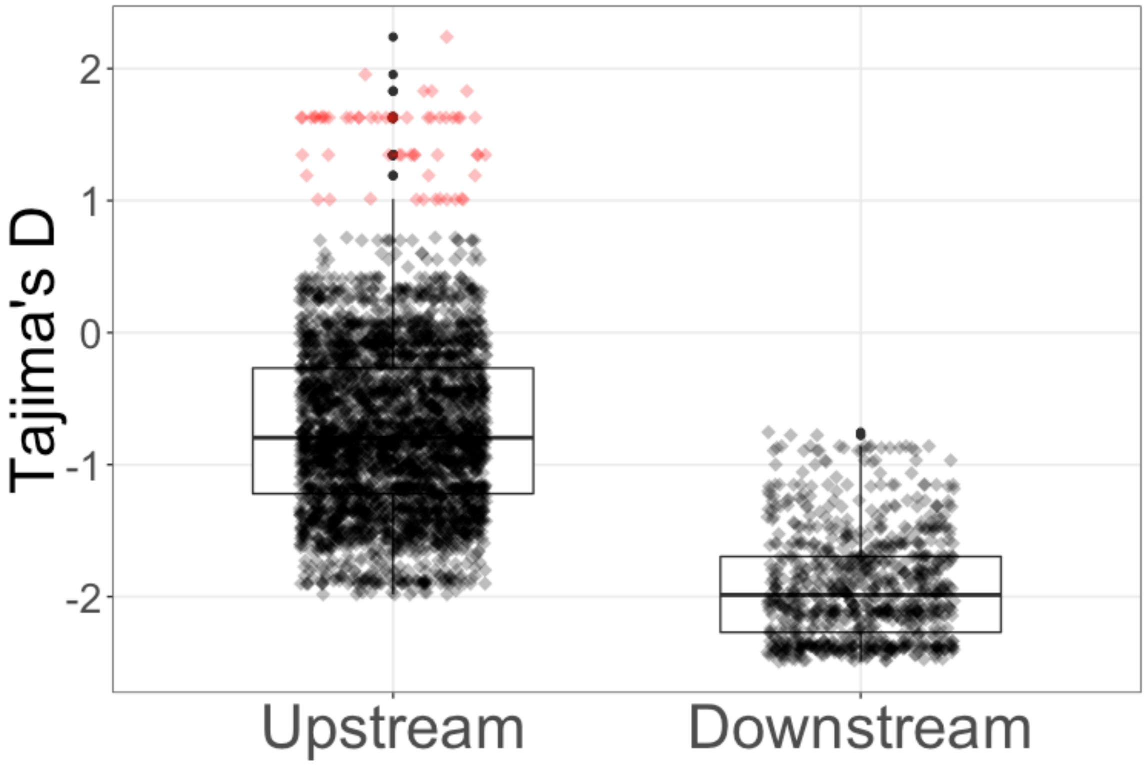

Figure S8: Distribution of social scores among the regulatory clades.
See the phylogenetic tree (Figure 2a) for allele identities and clades.

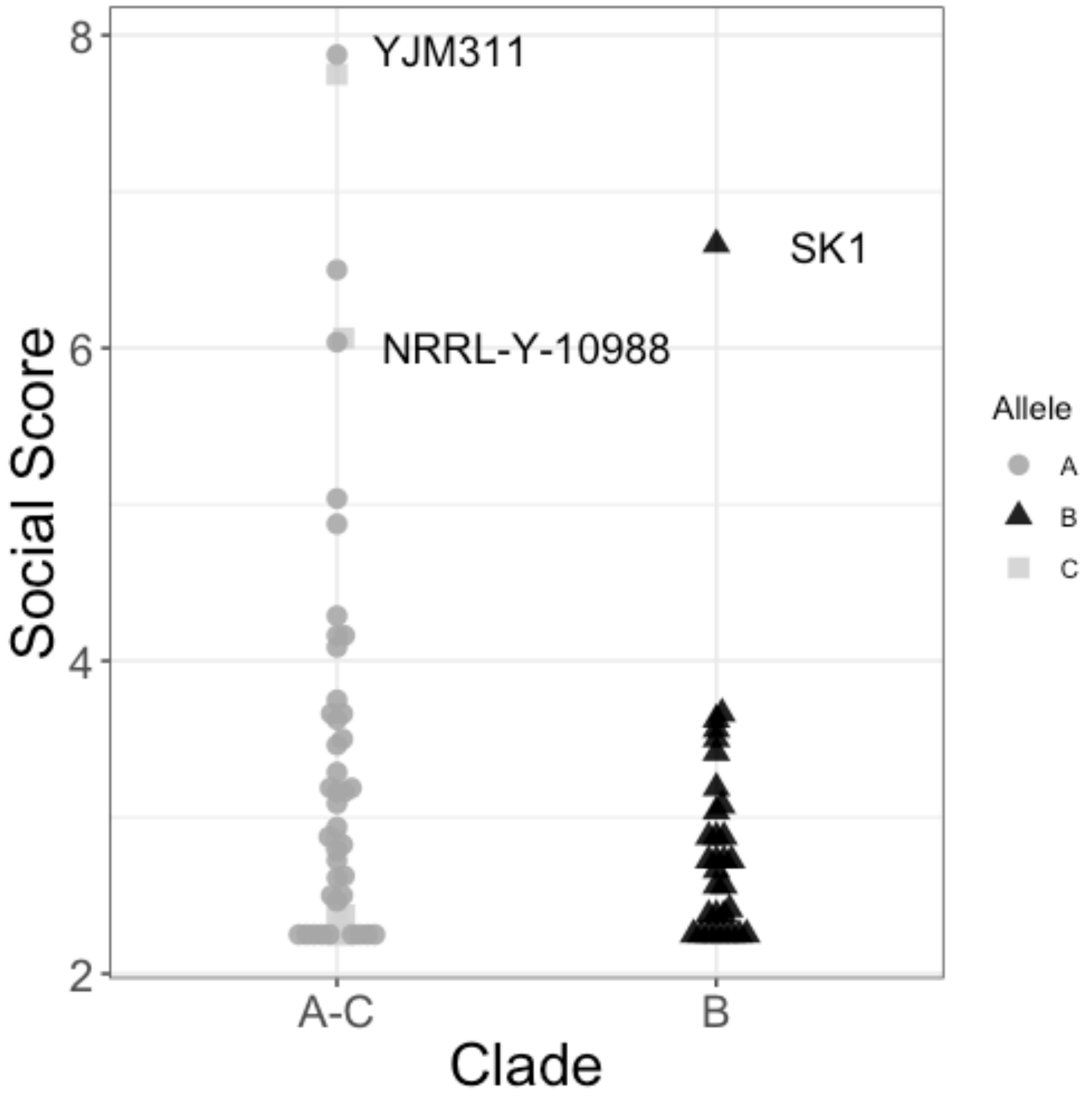

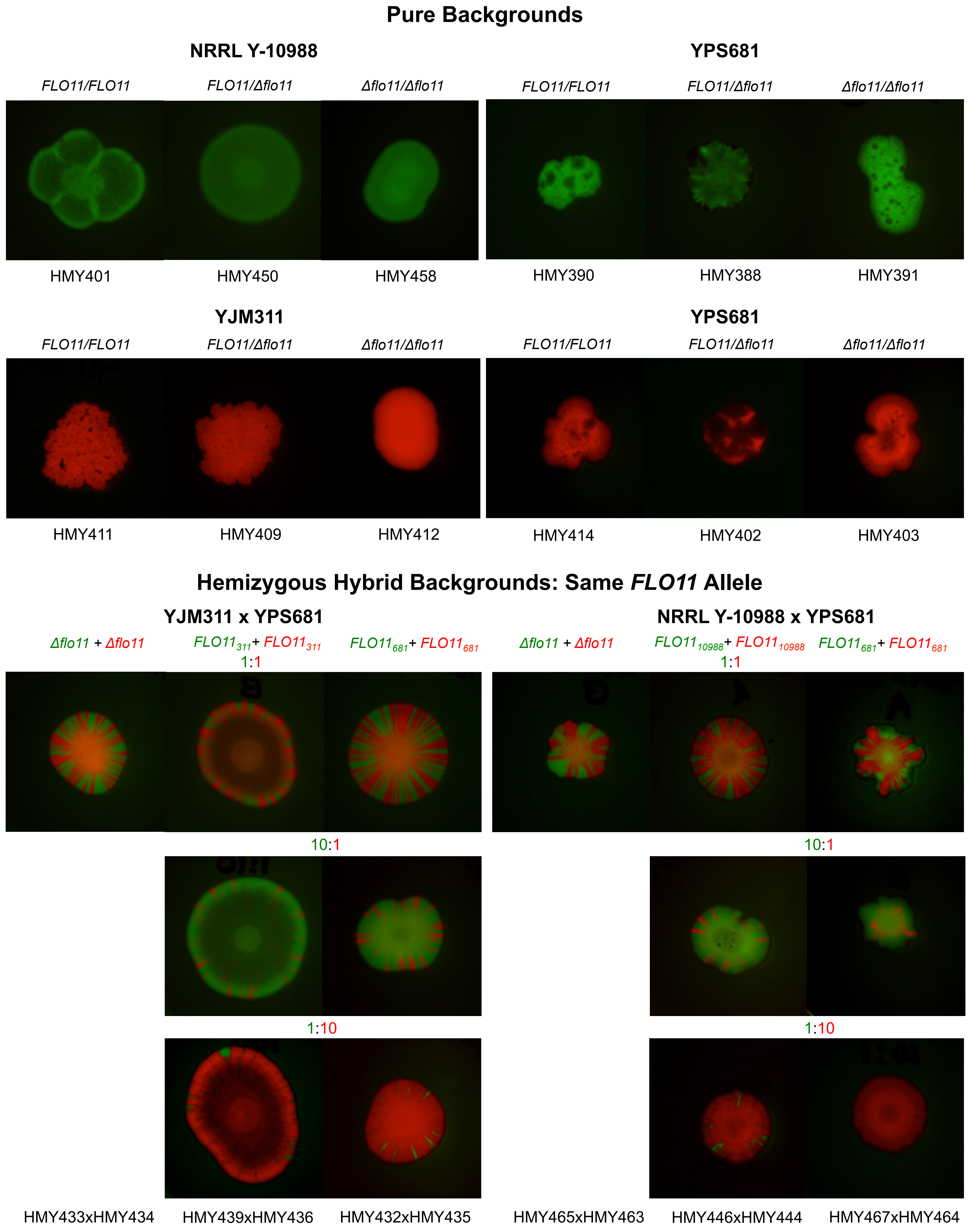
Pure Backgrounds
NRRL Y-10988
FLO11/FLO11
FLO11/Δflo11
Δflo11/Δflo11
HMY401
HMY450
HMY458
YPS681
FLO11/FLO11
FLO11/Δflo11
Δflo11/Δflo11
HMY390
HMY388
HMY391
YJM311
FLO11/FLO11
FLO11/Δflo11
Δflo11/Δflo11
HMY411
HMY409
HMY412
YPS681
FLO11/FLO11
FLO11/Δflo11
Δflo11/Δflo11
HMY414
HMY402
HMY403
Hemizygous Hybrid Backgrounds: Same FLO11 Allele
YJM311 x YPS681
Δflo11 + Δflo11
$FLO11_{311}$ + $FLO11_{311}$
$FLO11_{681}$ + $FLO11_{681}$
1:1
10:1
1:10
HMY433xHMY434
HMY439xHMY436
HMY432xHMY435
NRRL Y-10988 x YPS681
Δflo11 + Δflo11
$FLO11_{10988}$ + $FLO11_{10988}$
$FLO11_{681}$ + $FLO11_{681}$
1:1
10:1
1:10
HMY465xHMY463
HMY446xHMY444
HMY467xHMY464

NRRL Y-10988 x YJM311

Δflo11 + *Δflo11* — $FLO11_{311}$ + $FLO11_{311}$ — $FLO11_{10988}$ + $FLO11_{10988}$

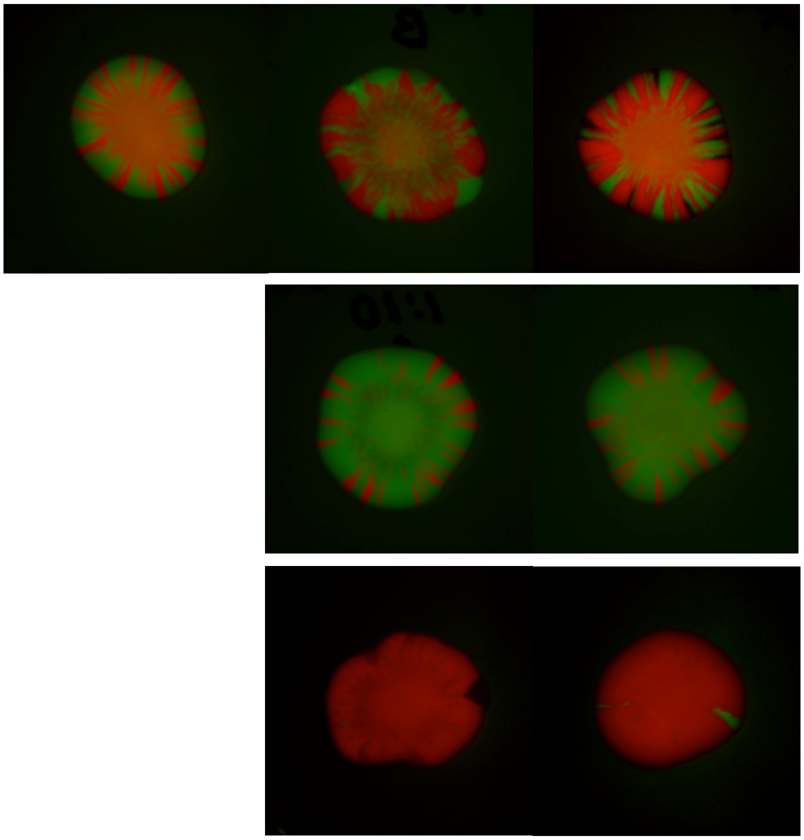

1:1

10:1

1:10

HMY466xHMY475 — HMY462xHMY468 — HMY442xHMY441

Figure S9: Functional assay of natural FLO11 alleles. Ratios indicate the volume of each strain used to create a mixed inoculum. **(A)** Three genetic backgrounds with varying levels of social phenotypes. **(B)** Hybrids of the three strains, hemizygous for the *FLO11* locus. Different alleles generate different mat architectures. Mixed colonies of GFP and mCherry versions of the same strain demonstrate no fitness cost to the fluorescence; numerically disadvantaged strains remain rare in the final mat. **(C)** Mixed colonies of hybrids with different *FLO11* alleles. The *FLO11* allele from NRRL Y-10988 appears to dominate the edge of the biofilm in mixed communities with other alleles, even when it is numerically disadvantaged. In contrast, in mixed communities with YJM311 and YPS681 *FLO11* alleles, neither appears to have a fitness advantage.

Hemizygous Hybrid Backgrounds: Different *FLO11* Alleles

YJM311 x YPS681 — **NRRL Y-10988 x YPS681** — **NRRL Y-10988 x YJM311**

$FLO11_{311}$ + $FLO11_{681}$ — $FLO11_{681}$ + $FLO11_{311}$ — $FLO11_{10988}$ + $FLO11_{681}$ — $FLO11_{681}$ + $FLO11_{10988}$ — $FLO11_{10988}$ + $FLO11_{311}$ — $FLO11_{311}$ + $FLO11_{10988}$

1:1

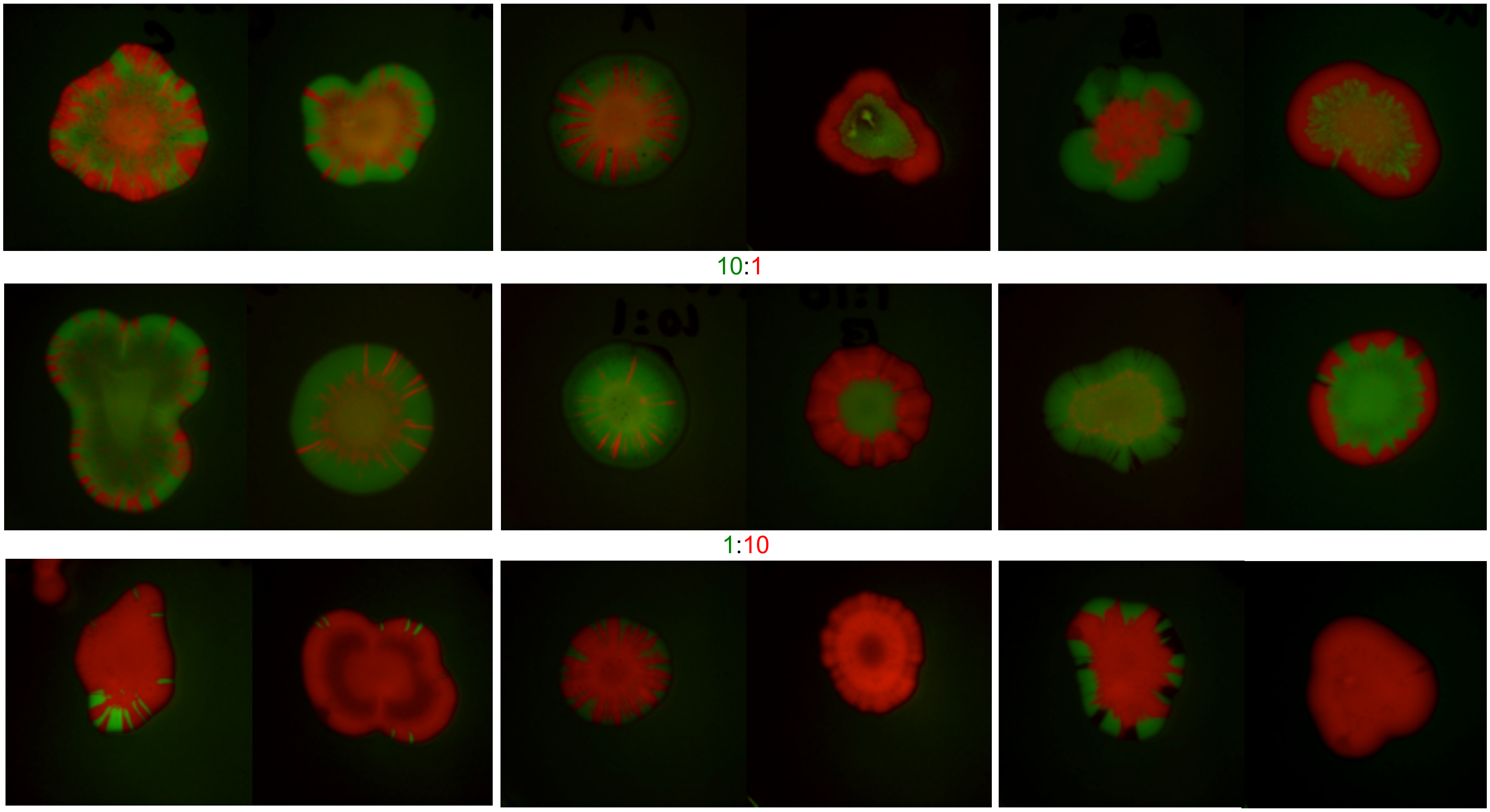

10:1

1:10

HMY439xHMY435 — HMY432xHMY436 — HMY446xHMY464 — HMY467xHMY444 — HMY442xHMY468 — HMY462xHMY441

Sample	HMY	Strain	Parental isolate/ isogenic with	Isolate source or other name	Geographic origin	Origin	Population	Upstream	Domain A	Domain C	Downstream	B Length	Reference
1	2		YJM224		Unknown	Distillery		✔	✔	✔	✔	✔	
2	3		YJM311	91-212	CA, USA	Clinical	Mosaic	✔	✔	✔	✔	✔	
4	25	YJM244	YJM210	CBS 1227	Romania	Clinical	Wine/Eur	✔	✔	✔	✔	✔	1
3	26	YJM248	YJM218	CBS 2910	Unknown	Clinical (feces)	Wine/Eur	✔	✔	✔	✔	✔	1
5	27	YJM270	YJM215	CBS 2807	Slovenia	Wine	Wine/Eur	X	X	X	X	X	1
6	30	YJM326	YJM310	CBS 7838	CA, USA	Clinical	Mosaic	✔	✔	✔	✔	X	1
7	31	YJM428	YJM308	CBS 7836	CA, USA	Clinical	Mosaic	✔	✔	✔	✔	✔	1
8	32	YJM450	YJM440	92-123	CA, USA	Clinical	Mosaic	✔	✔	✔	✔	✔	1
9	33	YJM451	YJM436	B70302(b)	Unknown	Clinical	Mosaic	✔	✔	✔	✔	✔	1
10	35	YJM456	YJM454	89-156	CA, USA	Clinical	Mosaic	✔	✔	✔	✔	X	1
11	36	YJM470	YJM455	SUH	CA, USA	Clinical	Mosaic	✔	✔	✔	✔	✔	1
12	37	YJM541	YJM522	SUH	CA, USA	Clinical	Mosaic	✔	✔	✔	✔	✔	1
13	38	YJM554	YJM521	SUH	CA, USA	Clinical	Mosaic	✔	✔	✔	✔	X	1
14	39	YJM555	YJM523	SUH	CA, USA	Clinical	Mosaic	✔	✔	✔	✔	X	1
15	43	YJM693	YJM669	R91-48	TX, USA	Clinical	Mosaic	✔	✔	✔	✔	✔	1
16	45	YJM969	YJM967	96-98	Italy	Clinical	Wine/Eur	✔	✔	✔	✔	✔	1
17	46	YJM689	YJM675	R93-1017	TX, USA	Clinical	Mosaic	✔	✔	✔	✔	✔	1
18	47	YJM972	YJM947	96-100	Italy	Clinical	Wine/Eur	✔	X	X	✔	X	1
19	57	YJM1078	YJM1075	NRRL YB-4348	Portugal	Clinical	Wine/Eur	✔	✔	✔	✔	✔	1
20	58	YJM1083	YJM1073	NRRL Y-10,988	NC, USA	Clinical	Mosaic	✔	✔	✔	✔	✔	1
21	59	YJM1129	YJM1123	NCMH 125	OH, USA	Distillery	Wine/Eur	✔	✔	✔	✔	✔	1
22	60	YJM1133	YJM1125	MMRL 125	NC, USA	Clinical	Mosaic	✔	✔	✔	✔	✔	1
23	62	YJM1199	YJM1115		MI, USA	Clinical	Mosaic	✔	✔	✔	✔	X	1
24	64	YJM1208	YJM1119	1882	MI, USA	Clinical	Mosaic	✔	✔	✔	✔	✔	1

25	67	YJM1248	YJM1219	NRRL Y-1546	West Africa	Wine	West African	✓	✓	✓	✓	✓	1
26	69	YJM1252	YJM1224	NRRL Y-6679	Spain	Alpechin	Wine/Eur	✓	✓	✓	✓	✓	1
27	72	YJM1290		Sigma1278b	Unknown	Laboratory	Mosaic	✓	✓	✓	✓	X	1
28	75	YJM1307	YJM1071	NRRL Y-961	DC, USA	Clinical	Mosaic	✓	✓	✓	✓	✓	1
29	76	YJM1311	YJM1101	C. Kaufman	MI, USA	Clinical	Mosaic	✓	✓	✓	✓	✓	1
30	80	YJM1338	YJM1315	NRRL Y-963	MD, USA	Sour fig	Mosaic	✓	✓	✓	✓	✓	1
31	82	YJM1342	YJM1324	NRRL Y-12638	South Africa	Soil	Mosaic	✓	✓	✓	✓	✓	1
32	85	YJM1381	YJM1357	NRRL YB-427	Trinidad	Rum fermentation	Mosaic	✓	✓	✓	✓	✓	1
33	88	YJM1386	YJM1365	NRRL Y-11878	Jamaica	Sugar cane	Mosaic	✓	✓	X	✓	✓	1
34	91	YJM1389	YJM1368	NRRL Y-17447	Thailand	Sewage	Sake	✓	✓	✓	✓	✓	1
35	92	YJM1399	YJM1393	NRRL YB-908	Unknown	Wild cherry tree gum	Mosaic	✓	✓	✓	✓	✓	1
36	93	YJM1400	YJM1394	NRRL YB-4081	Philippines	Guava	Mosaic	✓	✓	✓	✓	✓	1
37	96	YJM1415	YJM1407	NRRL Y-268	France	Wine	Wine/Eur	✓	✓	✓	✓	✓	1
38	97	YJM1417	YJM1411	NRRL YB-2541	HI, USA	ND	Wine/Eur	✓	✓	✓	✓	✓	1
39	98	YJM1418	YJM1413	NRRL YB-4506	Japan	Oak tree	Mosaic	✓	✓	✓	✓	✓	1
40	99	YJM1419	YJM1412	NRRL YB-2625	Unknown	Bagasse	Mosaic	✓	✓	✓	✓	✓	1
41	100	YJM1433		YIIc17_E5	France	Wine	Wine/Eur	✓	X	X	✓	X	1,2
42	103	YJM1443		UWOPS83-787.3	Bahamas	Fruit	Mosaic	✓	✓	✓	✓	✓	1,2
43	111	YJM1479	YJM1474	NRRL Y-6297	Phillipines	Coconut tuba	Mosaic	✓	✓	✓	✓	✓	1
44	117	YJM1615	YJM312	91-213	CA, USA	Clinical	Mosaic	✓	✓	✓	✓	✓	1
45	121	YJM1573	YJM1566	NRRL Y-12603	India	Fermented food	Mosaic	✓	✓	✓	✓	✓	1
46	122	YJM1552	S1	S288c	CA, USA	Rotten fig	Mosaic	✓	✓	✓	✓	✓	1
47	124	YJM1529		M22	Unknown	Wine	Wine/Eur	✓	✓	✓	✓	✓	1
48	158			YPS128	PA, USA	Oak	North American	✓	✓	✓	✓	✓	3
50	160			L-1528	Chile	Wine	Wine/Eur	✓	✓	✓	✓	X	2
49	161	YJM975	YJM948	96-101	Italy	Clinical	Wine/Eur	✓	X	X	✓	✓	1
51	169			SK1	USA	Soil, lab strain	West African	X	✓	✓	✓	✓	2
52	170			K11	Japan	Sake strain	Sake	✓	✓	✓	✓	✓	2
53	171			UWOPS05-217.3	Malaysia	Bertam Palm	Malaysian	✓	X	X	✓	✓	2
54	172			NCYC361	Ireland- *S.*	Beer spoilage	Mosaic	✓	✓	✓	✓	✓	2

					diastaticus								
55	173			DBVPG6765	unknown- *S. boulardi*	Unknown	Wine/Eur	✓	X	X	✓	X	2
56	174	YJM1549		DBVPG6040	Netherlands- *S. fructum*	Fermenting fruit juice	Mosaic	✓	✓	✓	✓	✓	1,2
57	175	YJM1463		DBVPG1853	Ethiopia	White tecc	Wine/Eur	✓	X	X	✓	✓	1,2
58	217	YJM145	YJM128	CBS 7833	MO, USA	Clinical		✓	✓	✓	✓	X	1
59	261	YJM978	YJM954	96-109	Italy	Clinical	Wine/Eur	✓	✓	✓	✓	✓	1
60	262	YJM1434		YPS606	PA, USA	Oak	North American	✓	✓	✓	✓	✓	3
61	263			L-1374	Chile	Wine	Wine/Eur	✓	X	X	✓	✓	2
62	264	YJM1460		Y12	Africa	Palm Wine	Sake	✓	✓	✓	✓	✓	1,2
63	265			YS2	Australia	Baker strain	Mosaic	✓	✓	✓	✓	✓	2
64	266	YJM1439		NCYC110	West Africa	Ginger beer	West African	✓	✓	✓	✓	X	1,2
65	267			DBVPG1106	Australia	Grape	Wine/Eur	✓	✓	✓	✓	✓	2
66	268			378604X	Newcastle, UK	Clinical	Mosaic	✓	✓	✓	✓	✓	2
67	270			YPS681	PA, USA	Oak	North American	✓	✓	✓	✓	✓	4
68	271			YPS615	PA, USA	Oak	North American	✓	✓	✓	✓	✓	4
69	272			YPS617	PA, USA	Oak	North American	✓	✓	✓	✓	✓	4
70	273			YPS661	PA, USA	Oak	North American	✓	✓	✓	✓	✓	4
71	274			YPS670	PA, USA	Oak	North American	✓	✓	✓	✓	✓	4
72	275			YPS630	PA, USA	Oak	North American	✓	✓	✓	✓	✓	4
73	276			UWOPS3-461.4	Malaysia	Bertam Palm, nectar	Malaysian	✓	✓	✓	X	✓	2
74	277			Y55	France	Wine, lab strain		X	✓	✓	✓	✓	2
75	278			YS4	Netherlands	Baker strain	Mosaic	✓	✓	✓	✓	✓	2
76	279			DBVPG1788	Finland	Soil	Wine/Eur	✓	✓	✓	✓	✓	2
77	280			DBVPG6044	West Africa	Bili wine	West African	✓	✓	✓	✓	✓	2
78	281			Y9; NRRL-Y5997	Japan	Ragi wine	Sake	✓	✓	✓	✓	X	2

Table S1. List of the 78 strains, along with their parental isolate name, geographic and environmental origin, as well as population membership (according to ref 1.). The data obtained for each strain is indicated with a check. (1) Strope, P.K., et al. (2015), *Genome Research*, 25(5):762-774. (2) Liti, G., et al. (2009), *Nature*, 458: 337-341. (3) Sniegowski, P.D., et al. (2002), *FEMS Yeast Research*, 1: 299-306. (4) Murphy, H.A. and C.W. Zeyl (2012), *Evolution*, 66(4): 1196-1209.

Systematic Name	Standard Name	cont'd	cont'd	cont'd	cont'd
YBR067C	TIP1	YIL011W	TIR3	YLR390W-A	CCW14
YBR162C	TOS1	YJL158C	CIS3	YMR238W	DFG5
YDR055W	PST1	YJL159W	HSP150	YMR305C	SCW10
YDR077W	SED1	YJL171C	TOH1	YMR307W	GAS1
YDR261C	EXG1	YJR004C	SAG1	YNL190W	n/a
YDR349C	YPS7	YKL046C	DCW1	YNL300W	TOS6
YEL040W	UTR2	YKL096W	CWP1	YNL322C	KRE1
YER150W	SPI1	YKL096W-A	CWP2	YNL327W	EGT2
YGL028C	SCW11	YLR040C	AFB1	YNR044W	AGA1
YGL228W	SHE10	YLR042C	n/a	YOL132W	GAS4
YGR279C	SCW4	YLR110C	CCW12	YOR009W	TIR4
YHL009C	YAP3	YLR194C	NCW2	YOR010C	TIR2
YHR126C	ANS1	YLR342W	FKS1	YOR214C	SPR2
YHR143W	DSE2	YLR343W	GAS2	YPR159W	KRE6

Table S2: Genes recovered from literature search for GPI-anchored and cell wall associated proteins.

Sample	HMY	Strain	cont'd	cont'd	cont'd	cont'd	cont'd	cont'd
3	HMY26	CBS2910[1]	22	HMY60	MMRL 125[1]	44	HMY117	91-213[1]
4	HMY25	CBS1227[1]	23	HMY62	YJM1115[1]	45	HMY121	NRRL Y-12603[1]
5	HMY27	CBS2807[1]	24	HMY64	YJM1119[1]	46	HMY122	S288c[2]
6	HMY30	CBS7838[1]	25	HMY67	NRRL Y-1546[1]	48	HMY158	YPS128[2]
7	HMY31	CBS7836[1]	26	HMY69	NRRL Y-6679[1]	49	HMY161	96-101[1]
8	HMY32	92-123[1]	28	HMY75	NRRL Y-961[1]	51	HMY169	SK1[2]
9	HMY33	YJM436[1]	29	HMY76	YJM1101[1]	55	HMY173	DBVPG6765[2]
10	HMY35	89-156[1]	30	HMY80	NRRL Y-963[1]	56	HMY174	DBVPG6040[1]
11	HMY36	YJM455[1]	32	HMY85	NRRL YB-427[1]	57	HMY175	DBVPG1853[1]
12	HMY37	YJM522[1]	34	HMY91	NRRL Y-17447[1]	57	HMY175	DBVPG1853
13	HMY38	YJM521[1]	35	HMY92	NRRL YB-908[1]	59	HMY261	96-109[1]
14	HMY39	YJM523[1]	36	HMY93	NRRL YB-4081[1]	60	HMY262	YPS606
15	HMY43	R91-48[1]	37	HMY96	NRRL Y-268[1]	62	HMY264	Y12[1]
16	HMY45	96-98[1]	38	HMY97	NRRL YB-2541[1]	62	HMY264	Y12[2]
17	HMY46	R93-1017[1]	39	HMY98	NRRL YB-4506[1]	64	HMY266	NCYC110[1]
18	HMY47	96-100[1]	40	HMY99	NRRL YB-2625[1]	73	HMY276	UWOPS3-461.4[2]
20	HMY58	NRRL Y-10988[1]	41	HMY100	YJM1433[1]	77	HMY280	DBVPG6044[2]
21	HMY59	NCMH 125[1]	43	HMY111	NRRL Y-6297[1]	-	HMY269	UWOPS83-787.3[1]

Table S3: Strains with available genomes used for gene comparison to *FLO11*.
[1] Strope, P.K., et al. (2015), *Genome Research*, 25(5):762-774; [2] Yue, J.-X., et al. (2017), *Nature Genetics*, 49(6):913-924.

	HMY	Genetic Background	*PGK1*-Fluorescence	*FLO11*
Pure Background	390	YPS681	GFP-KanMX	*FLO11*/*FLO11*
	388	YPS681	GFP-KanMX	*FLO11*/*Δflo11*
	391	YPS681	GFP-KanMX	*Δflo11*/*Δflo11*
	414	YPS681	mCherry-KanMX	*FLO11*/*FLO11*
	402	YPS681	mCherry-KanMX	*FLO11*/*Δflo11*
	403	YPS681	mCherry-KanMX	*Δflo11*/*Δflo11*
	411	YJM311	mCherry-HphMX	*FLO11*/*FLO11*
	409	YJM311	mCherry-HphMX	*FLO11*/*Δflo11*
	412	YJM311	mCherry-HphMX	*Δflo11*/*Δflo11*
	401	NRRL Y-10988	GFP-KanMX	*FLO11*/*FLO11*
	450	NRRL Y-10988	GFP-KanMX	*FLO11*/*Δflo11*
	458	NRRL Y-10988	GFP-KanMX	*Δflo11*/*Δflo11*
Hybrid Background	435	YPS681 x YJM311	mCherry-HphMX	$FLO11_{681}$/*Δflo11*
	436	YPS681 x YJM311	mCherry-HphMX	$FLO11_{311}$/*Δflo11*
	434	YPS681 x YJM311	mCherry-HphMX	*Δflo11*/*Δflo11*
	432	YPS681 x YJM311	GFP-KanMX	$FLO11_{681}$/*Δflo11*
	439	YPS681 x YJM311	GFP-KanMX	$FLO11_{311}$/*Δflo11*
	433	YPS681 x YJM311	GFP-KanMX	*Δflo11*/*Δflo11*
	464	YPS681 x NRRL Y-10988	mCherry-KanMX	$FLO11_{681}$/*Δflo11*
	444	YPS681 x NRRL Y-10988	mCherry-KanMX	$FLO11_{10988}$/*Δflo11*
	463	YPS681 x NRRL Y-10988	mCherry-KanMX	*Δflo11*/*Δflo11*
	467	YPS681 x NRRL Y-10988	GFP-KanMX	$FLO11_{681}$/*Δflo11*
	446	YPS681 x NRRL Y-10988	GFP-KanMX	$FLO11_{10988}$/*Δflo11*
	465	YPS681 x NRRL Y-10988	GFP-KanMX	*Δflo11*/*Δflo11*
	468	YJM311 x NRRL Y-10988	mCherry-HphMX	$FLO11_{311}$/*Δflo11*
	441	YJM311 x NRRL Y-10988	mCherry-HphMX	$FLO11_{10988}$/*Δflo11*
	475	YJM311 x NRRL Y-10988	mCherry-HphMX	*Δflo11*/*Δflo11*
	462	YJM311 x NRRL Y-10988	GFP-KanMX	$FLO11_{311}$/*Δflo11*
	442	YJM311 x NRRL Y-10988	GFP-KanMX	$FLO11_{10988}$/*Δflo11*
	466	YJM311 x NRRL Y-10988	GFP-KanMX	*Δflo11*/*Δflo11*

HMY	Genetic Background	Fluorescence	*FLO11*
483	NRRL Y-10988	*prFLO11-GFP*	$FLO11_{10988}$/ *Δflo11::GFP-KanMX*
484	YJM311 x NRRL Y-10988	$prFLO11_{311}$*-GFP*	$FLO11_{10988}$/ *Δflo11::GFP-KanMX*
485	YJM311	*prFLO11-GFP*	$FLO11_{311}$/ *Δflo11::GFP-KanMX*
486	YJM311 x NRRL Y-10988	$prFLO11_{10988}$*-GFP*	$FLO11_{311}$/ *Δflo11::GFP-KanMX*

Table S4: Strains engineered for functional analysis of *FLO11*.

Primer	Sequence	Design/Purpose
FLO11A-for<br>FLO11A-rev	TTGGTCAATCAGAACAGGCAAC<br>GAGACATCTTTAGAGTAACCACAGATATTC	To amplify *FLO11* and its associated regulatory regions
PGK1-YRC-for<br>PGK1-YRC-rev	GGTAAGGAATTGCCAGGTGTTGCTTTCTTATCCGAAAAGA AA GGTCGACGGATCCCCGGG<br>GAAAAGAAAAAAATTGATCTATCGATTTCAATTCAATTCAAT ATCGATGAATTCGAGCTCG	To generate a cassette to produce fluorescence. 40 bp upstream and downstream of the *PGK1* stop codon plus homology to the pFA6a plasmid (GFP-KanMX cassette) and pBS34 (mCherry-KanMX)
FLO11-MX-for<br>FLO11-MX-rev	GACCCCTTTTCGTACAGAAGCCTTGGTCAATCAGAACAGG CAACG CGTACGCTGCAGGTC<br>ACAGT GGCTTCAAAG AACTGCTGATTGCTCAAGGC AATCAGTCCG ATCGATGAATTCGAG	To generate a cassette to delete *FLO11* and replace with antibiotic resistance gene. 45 bp homology to upstream and downstream of *FLO11* plus homology to antibiotic MX cassettes.
FLO11-end-Kan-for<br>FLO11-pr-GFP-rev	AACATCGTAATGAAGAAACGAACATGTTGGAATTGTATCA ATCGATGAATTCGAGCTCG<br>TACTTTTGTAGGCCTCAAAAATCCATATACGCACACT ATG AGTAAAGGAGAAGAACTTTT	To generate a cassette to replace *FLO11* with GFP. Homology to promoter of *FLO11*, ATG, and the 2nd codon on in GFP; homology to downstream of *FLO11* and the end of the KanMX cassette.
FLO11-Repeat-for<br>FLO11-Repeat-rev	GGTTTCGCTTGGACTGGTTGAACATGGAAC<br>GATTTCCCAGGCTTCTATTGGAACATAGAT	To amplify the B-domain for length analysis.

Table S5. Primers used in this study

Tree	Model	Parameter estimates	Log Likelihood	No. Param.	AIC	Comparison	Chi-squared	P-value
A-domain	Model1	p_0=0.70, p_1= 0.30, ω_0=0.016, κ=2.23	-2059.398	103	4324.8	2 vs. 1	49.106	<0.0001
	Model2	p_0=0.70, p_1= 0.21, p_2=0.09, ω_0=0.045, ω_1=1, ω_2=5.24, κ=3.04	-2034.845	105	4279.7			
	Model7	p=0.008, q= 0.016, κ=2.24	-2059.504	104	4327.0	8 vs. 7	48.966	<0.0001
	Model8	p=0.06, q= 0.18, p_0=0.91, p_1= 0.09 ω=5.25, κ=3.05	-2035.021	106	4282.0			
	ModelC	ω=0.60,π=3.32, κ=2.79	-2100.346	113	4426.7	E vs. C	39.858	<0.0001
	ModelE	ω_1=0.43, ω_2=4.51, π=3.09, κ_1=3.71, κ_2=1.30	-2080.417	115	4390.8			
A-C Concatenate	Model1	p_0=0.71, p_1=0.29 , ω_0=0, κ=3.82	-4485.937	111	9193.9	2 vs. 1	222.014	<0.0001
	Model2	p_0=0.69, p_1=0.27, p_2=0.04, ω_0=0.02, ω_1=1, ω_2=15.98, κ=5.04	-4374.93	113	8975.9			
	Model7	p=0.005, q=.012 , κ=3.76	-4484.238	112	9192.5	8 vs. 7	218.568	<0.0001
	Model8	p=0.005, q=0.01, p_0=0.96, p_1= 0.04, ω=16.25, κ=5.05	-4374.954	114	8977.9			
	ModelC	ω=0.62,π=5.69, κ=3.83	-4506.825	121	9255.7	E vs. C	47.642	<0.0001
	ModelE	ω_1=0.50, ω_2=4.60, π=5.24, κ_1=5.08, κ_2=1.30	-4483.004	123	9212.0			
A-B-C Concatenate	Model1	p_0=0.70, p_1= 0.30, ω_0=0.007, κ=2.69	-6626.686	111	13475.4	2 vs. 1	250.794	<0.0001
	Model2	p_0=0.67, p_1= 0.31, p_2=0.02, ω_0=0, ω_1=1, ω_2=15.63, κ=3.18	-6501.289	113	13228.6			
	Model7	p=0.01, q= 0.02, κ=2.70	-6626.727	112	13477.5	8 vs. 7	284.204	<0.0001
	Model8	p=0.007, q= 0.01, p_0=0.97, p_1= 0.03, ω=13.7, κ=3.25	-6484.625	114	13197.3			
	ModelC	ω=0.53, π=3.46, κ=2.63	-6724.781	121	13691.6	E vs. C	41.526	<0.0001
	ModelE	ω_1=0.46, ω_2=5.32, π=3.22, κ_1=2.69, κ_2=1.45	-6704.018	123	13654.0			
GPI-Anchored Proteins	Model1	p_0=0.83, p_1= 0.17, ω_0=0.003, κ=3.05	-7310.787	77	14775.6	2 vs. 1	888.794	<0.0001
	Model2	p_0=0.81, p_1= 0.13, p_2=0.06, ω_0=0.009, ω_1=1, ω_2=16.2, κ=4.28	-6866.390	79	13890.8			
	Model7	p=0.009, q= 0.055, κ=3.03	-7320.583	78	14797.2	8 vs. 7	906.836	<0.0001
	Model8	p=0.009, q= 0.051, p_0=0.94, p_1= 0.06, ω=15.9, κ=4.27	-6867.165	80	13894.3			
	ModelC	ω=0.72,π=6.16, κ=3.15	-7527.352	87	15228.7	E vs. C	103.500	<0.0001
	ModelE	ω_1=0.57, ω_2=7.82, π=5.81, κ_1=4.02, κ_2=1.35	-7475.602	89	15129.2			

Table S6: Results of the evolutionary analysis conducted in PAML using three different phylogenetic trees with their associated alignment, as well as a "strain" tree with an alignment of the fullest data set (i.e., including the part of the B domain that could be resolved and aligned). Parameter estimates associated with each model are listed: ω= dN/dS rate; π= synonymous transversion rate; κ= transition/transversion rate; parameters with subscripts refer to estimates for a given site class; p_i= proportion of codons in a given site class; p and q are parameters describing a β distribution. For each data set, the random sites models that allow for the possibility of positive selection (2 and 8) were significantly better fits than those that did not (1 and 7), as determined by a likelihood ratio test. Likewise, the fixed sites model that allowed the dN/dS value to differ between the site partitions (E) was a significantly better model of the data than the model that held dN/dS constant between the partitions (C).

A-Domain Tree

Model 2	54D, 57N, 94W, 177Q, 183Q, 190D
Model 8	17N, 54D, 57N, 94W, 113Y, 119-, 177Q, 179A, 180S, 183Q, 190D

Concatenated A- and C-Domains Tree

Model 2	17N, 54D, 57N, 94W, 113Y, 114-, 118-, 119-, 177Q, 179A, 180S, 183Q, 190D, 200N, 203H, 406V(1188)
Model 8	17N, 54D, 57N, 94W, 113Y, 114-, 118-, 119-, 134Y, 177Q, 179A, 180S, 183Q, 190D, 203H, 375K(1157), 406V(1188)

Concatenated A-, minor B- and C- Domains Tree

Model 2	54D, 94W, 113Y, 114-, 116-, 119-, 177Q, 190D, 203H, [220S(855), 228V(863), 299P(934)] 522K(1157)
Model 8	54D, 94W, 113Y, 114-, 116-, 118-, 119-, 177Q, 183Q, 190D, 203H, [220S(855), 228V(863), 299P(934)] 522K(1157)

Genes of GPI-Anchored and Cell Wall Proteins Tree

Model 2	4P, 6L, 7L, 17N, 54D, 57N, 60Q, 85K, 91K, 94W, 106G, 113Y, 114-, 118-, 119-, 129-, 131E, 133T, 172Q, 173G, 176A, 177Q, 178Y, 179A, 182W, 189F, 195C, 202G, [219V(854), 227S(862), 290T(925), 298T(933), 327T(962), 334T(969), 335T(970), 338T(973), 343S(978),] 521G(1156), 550T(1185), 552A(1187), 675S(1310), 731V(1366)
Model 8	4P, 6L, 7L, 17N, 54D, 57N, 60Q, 85K, 91K, 94W, 106G, 113Y, 114-, 118-, 119-, 129-, 131E, 133T, 172Q, 173G, 175A, 176A, 177Q, 178Y, 179A, 182W, 189F, 195C, 202G, [219V(854), 227S(862), 290T(925), 298T(933), 327T(962), 334T(969), 335T(970), 338T(973), 343S(978),] 521G(1156), 550T(1185), 552A(1187), 675S(1310), 731V(1366)

Table S7: Under random sites models that allowed for positive selection (M2 and M8), individual codons were identified as being under significant positive selection. Red and blue codons are associated with the apical regions of the A-domain and grey codons fall within the part of the B-domain that was included in the analysis (see Figure S4). Numbers refer to the amino acid within the concatenated A and C-domain alignment and numbers in parentheses refer to the amino acid within the reference strain, S288c, *FLO11* sequence (P>0.95).

Tree	Model	Parameter estimates	Log Likelihood	No. Param.	AIC	Comparison	Chi-squared	P-value
A-C strains with insert	Model1	p_0=0.58, p_1= 0.42, ω_0=0.00, κ=1.83	-2741.054	43		2 vs. 1	105.82	<0.0001
	Model2	p_0=0.96, p_1= 0.0003, p_2=0.04, ω_0=0.439, ω_1=1, ω_2=63.9, κ=2.97	-2688.146	45				
	Model7	p=2.28, q= 0.005, κ=1.93	-2747.600	44		8 vs. 7	118.91	<0.0001
	Model8	p=65.4, q= 83.7, p_0=0.96, p_1= 0.04 ω=63.9, κ=2.97	-2688.146	46				
	ModelC	ω=11.90,π=2.14, κ=2.14	-2691.529	53		E vs. C	11.52	0.00069
	ModelE	ω_1=1.30, ω_2=999, π=11.59, κ_1=3.16, κ_2=1.42	-2685.768	55				
A-C strains without insert	Model1	p_0=0.72, p_1=0.28 , ω_0=0, κ=4.61	-3774.625	76		2 vs. 1	91.292	<0.0001
	Model2	p_0=0.75, p_1=0.23, p_2=0.02, ω_0=0.058, ω_1=1, ω_2=17.29, κ=5.46	-3728.979	78				
	Model7	p=0.005, q=.012 , κ=4.63	-3774.725	77		8 vs. 7	92.664	<0.0001
	Model8	p=0.006, q=0.01, p_0=0.99, p_1= 0.01, ω=36.66, κ=5.52	-3728.393	79				
	ModelC	ω=0.42,π=3.95, κ=4.61	-3787.932	86		E vs. C	23.772	<0.0001
	ModelE	ω_1=0.37, ω_2=1.9, π=3.52, κ_1=5.62, κ_2=1.19	-3776.046	88				

Table S8: The sequence data that included the A- and C- domains concatenated was used to investigate whether separating the strains into groups with and without the insert (in the first interacting region of the protein) would affect the results. Models and parameters are as in Table S2. For both data sets, the random sites models that allow for the possibility of positive selection (2 and 8) were significantly better fits than those that did not (1 and 7), as determined by a likelihood ratio test. Likewise, the fixed sites model that allowed the dN/dS value to differ between the site partitions (E) was a significantly better model of the data than the model that held dN/dS constant between the partitions (C).

Concatenated A- and C- Domains: Strains with Insert

Model 2	4P, 17Y, 94S, 114D, 116N, 118D, 119W, 130D, 134Y, 177E, 178Y, 179A, 190Y, 196D, 200N, 203Y
Model 8	4P, 17Y, 94S, 114D, 116N, 118D, 119W, 130D, 134Y, 177E, 178Y, 179A, 190D, 196D, 200N, 203Y

Concatenated A- and C- Domains: Strains without Insert

Model 2	54D, 57N, 113Y, 164S(180)*, 167Q(183), 359K(375,1010), 390V(406,1188), 569F(585,1204)*
Model 8	54D, 57N, 113Y, 161Q (177)*, 164S(180), 167Q(183), 359K(375,1010), 390V(406,1188), 513V(529,1164)*, 569F(585,1204)

Table S9: Under random sites models that allowed for positive selection (M2 and M8), individual codons were identified as being under significant positive selection. Red and blue codons are associated with the apical regions of the A-domain (see Figure S4). Numbers refer to the amino acid within the concatenated A and C-domain alignment and numbers in parentheses refer to the amino acid when the insert is included in the sequence followed by the location in the reference sequence of S288C (P>0.95). * P>0.92

	Break Points	AICc	Δ AICc	Segment	p-value
A Domain	0	4025.05		1-648	
	1	3935.37	89.68	1-399, 400-648	0.0002
A-C Concatenated	0	8739.24		1-1755	
	1	8538.30	200.94	1-617, 618-1755	0.0004
	2	8471.75	66.55	1-440, 441-890, 891-1755	0.0004
A-B-C Concatenated	0	13263.88		1-2199	
	1	12728.38	535.50	1-698, 699-2199	0.0004
	2	12700.43	27.96	1-461, 462-727, 728-2199	0.0004

Table S10: Results from the GARD analysis for each data set. In the concatenated data sets, the locations refer to the location of the input sequence and not the reference genome sequence (i.e., the majority of the B-domain is missing). The A-domain is the first 648 nucleotides; the interacting domains are 336-393 and 513-546.

Tree	Model	Parameter estimates	Log Likelihood	No. Param.	AIC	Comparison	Chi-squared	P-value
Section 1: 1-462	Model1	p_0=0.74, p_1= 0.26, ω_0=0.025, κ=2.71	-1364.213	100	2928.4	2 vs. 1	49.778	<0.0001
	Model2	p_0=0.74, p_1= 0.19, p_2=0.07, ω_0=0.055, ω_1=1, ω_2=9.63, κ=4.00	-1339.324	102	2882.6			
	Model7	p=0.008, q=0.016, κ=2.79	-1364.425	101	2934.9	8 vs. 7	49.978	<0.0001
	Model8	p=0.089, q= 0.276, p_0=0.93, p_1= 0.07, ω=9.54, κ=4.02	-1339.436	103	2886.9			
	ModelC	ω=0.48,π=4.34, κ=3.10	-1392.434	110	3004.9	E vs. C	17.42	<0.0001
	ModelE	ω_1=0.379, ω_2=3.53, π=4.28, κ_1=3.72, κ_2=1.92	-1383.724	112	2991.5			
Section 2: 463-730	Model1	p_0=0.77, p_1=0.23 , ω_0=0.077, κ=1.49	-1617.268	97	3428.5	2 vs. 1	126.38	<0.0001
	Model2	p_0=0.63, p_1=0.30, p_2=0.07, ω_0=0.059, ω_1=1, ω_2=8.55, κ=2.23	-1554.078	99	3306.2			
	Model7	p=0.146, q= 0.35, κ=1.49	-1618.957	98	3433.9	8 vs. 7	130.89	<0.0001
	Model8	p=0.157, q= 0.316, p_0=0.93, p_1= 0.07, ω=8.06, κ=2.19	1553.513	100	3307.1			
	ModelC	ω=0.72,π=1.64, κ=2.09	-1703.781	107	3621.6	E vs. C	48.11	<0.0001
	ModelE	ω_1=0.49, ω_2=21.59, π=1.49, κ_1=2.16, κ_2=1.48	-1679.725	109	3577.5			
Section 3: 731-2199	Model1	p_0=0.66, p_1= 0.34, ω_0=0, κ=3.6	-3445.500	97	7085	2 vs. 1	68.266	<0.0001
	Model2	p_0=0.8, p_1= 0.16, p_2=0.04, ω_0=0.125, ω_1=1, ω_2=9.835, κ=3.99	-3411.367	99	7020.7			
	Model7	p=0.005, q=0.0096, κ=3.61	-3446.033	98	7088.1	8 vs. 7	61.236	<0.0001
	Model8	p=0.331, q=0.923, p_0=0.96, p_1= 0.04, ω=9.588, κ=4.01	-3415.415	100	7030.83			

Table S11: The sequence data that included the A-, minor B-, and C- domains concatenated was divided into three recombination blocks by the program GARD (nucleotides 1-462, 463-730, 731-2199); this division separated the two cell-cell interacting regions of the protein. Each block was separately analyzed by the program PAML; models and parameters as in Table S2. For all individual recombination blocks, the random sites models that allow for the possibility of positive selection (2 and 8) were significantly better fits than those that did not (1 and 7), as determined by a likelihood ratio test. Likewise, the fixed sites model that allowed the dN/dS value to differ between the site partitions (E) was a significantly better model of the data than the model that held dN/dS constant between the partitions (C).

Each Recombination Block Analyzed Separately

Model 2	54D, 57N, 94W, 113Y*, 114-**, 116-*, 118-**, 119-**, 177Q, 180S, 183Q, 190D, [220S(855), 228V(863), 256S(891), 274Q(909), 295S(930), 299P(934)], 551N(1186)
Model 8	54D, 57N, 94W, 113Y**, 114-**, 116-**, 118-**, 119-, 177Q, 180S, 183Q, 190D, [220S(855), 228V(863), 256S(891), 257F(892), 274Q(909), 295S(930), 299P(934)], 551N(1186), 732F(1367)

Table S12: Under random sites models that allowed for positive selection (M2 and M8), individual codons were identified as being under significant positive selection. Red and blue codons are associated with the apical regions of the A-domain and grey codons fall within the part of the B-domain that was included in the analysis (see Figure S4). Numbers refer to the amino acid within the concatenated A, B, and C-domain alignment and numbers in parentheses refer to the amino acid within the reference strain, S288c, *FLO11* sequence (P>0.95). **P>0.9, * P>0.86;

Source	Sum of Squares	F-ratio	p-value
Allele	0.1819	26.47 (1,82)	<0.0001
Medium	3.2845	478.09 (1,82)	<0.0001
Genetic Background	1.5437	224.70 (1,82)	<0.0001
Allele*Medium	0.0306	4.45 (1,82)	0.038
Allele*Background	0.0653	9.51(1,82)	0.0028
Medium*Background	1.1716	170.54 (1,82)	<0.0001
Allele*Medium*Background	0.0057	0.83 (1,82)	0.3657
**Genetic Background Contrast:** Hybrid *pFLO11*$_{10988}$ vs. Hybrid *pFLO11*$_{311}$		2.18 (1,82)	0.1439

Table S13. ANOVA results for *prFLO11-GFP* expression.
